# Supplementary material for: Finding the Sweet Spot: An Interactive Workshop on Diabetes Management in Older Adults
Source: MedEdPORTAL. 2019 Oct 18;15:10845. doi: 10.15766/mep_2374-8265.10845 (PMC6944249; doi:10.15766/mep_2374-8265.10845)
Supplement: Supplementary file 1 — A. Presurvey.docx B. Finding the Sweet Spot Slides.pptx C. Finding the Sweet Spot Activity.docx D. Considerations for A1c Targets.pptx E. Noninsulin Pharmacologic Options.pptx F. Insulin Pharmacologic Options.pptx G. Approach to Prescribing and Deprescribing.pptx H. Postsurvey.docx I. Pre- and Postsurvey Answer Guide.docx [file mep-15-10845-s001.zip › _Educational_Summary_Report_10845.pdf]

MedEdPORTAL® The AAMC Journal of Teaching and Learning Resources

Original Publication Open Access

Finding the Sweet Spot: An Interactive Workshop on Diabetes Management in Older Adults

Laura K. Triantafylidis, PharmD*, Sarah C. Phillips, MD, Chelsea E. Hawley, PharmD, Andrea Wershof Schwartz, MD, MPH

*Corresponding author: laura.triantafylidis@va.gov

Abstract

Introduction: Intensive glucose lowering in older adults with diabetes leads to increased risks with minimal benefits. Surveys indicate that clinician confidence for individualizing glycemic goals and regimens remains low. We created an interactive workshop and clinical tool kit to improve clinician knowledge of safe diabetes management in older adults. **Methods:** Finding the Sweet Spot was a 1-hour workshop taught by pharmacists to medical and pharmacy learners that introduced a five-step framework for diabetes management in older adults. The interactive presentation included cases and a clinical tool kit based on current recommendations from the American Diabetes Association and American Geriatrics Society. Pilot workshops were held for 6 months, allowing for real-time revisions based on feedback; final implementation occurred for 6 months thereafter. We evaluated learner self-efficacy (via a 5-point Likert scale) and knowledge (via multiple-choice questions) of diabetes management in older adults before and after the workshop. **Results:** Thirty learners participated in Finding the Sweet Spot (70% medicine, 30% pharmacy). The percentage of confident learners increased from 55% to 97% ($p < .05$) after the workshop. All learners demonstrated improvements in knowledge, with the mean score on the knowledge assessment increasing from 61% to 80% ($p < .05$). Via open-ended feedback, learners expressed satisfaction and found the clinical tool kit especially helpful. **Discussion:** Our Finding the Sweet Spot workshop demonstrated statistically significant changes in self-efficacy and knowledge among learners, indicating that this interactive workshop improves medical and pharmacy provider confidence and skills in caring for older adults with diabetes.

Keywords
Older Adults, Diabetes, Geriatric Diabetes, Deprescribing, Deprescriptions, Safe Prescribing, Editor's Choice

Educational Objectives

By the end of this workshop, learners will be able to:

1. List safety and efficacy characteristics of antihyperglycemic agents.
2. Develop individualized glycemic goals for treatment of older adults with diabetes.
3. Implement geriatric prescribing principles for antihyperglycemic agents.

Introduction

Clinician proficiency in the management of older adults with diabetes is essential, given that approximately 25% of older adults have diabetes and 50% have prediabetes.[1] Older adults with diabetes are medically complex: They have high rates of premature death, functional disability, and accelerated muscle loss, as well as multimorbidity related to hypertension, coronary artery disease, and/or stroke.[2] For older adults, the American Diabetes Association (ADA) Standards of Medical Care in Diabetes and the American Geriatrics Society (AGS) Guidelines for Improving the Care of Older Adults With Diabetes Mellitus recommend less stringent glycemic targets,[3,4] as evidence shows that the harms of intensive glucose control outweigh the benefits in this population.[3-7] However, studies indicate that most older adults with diabetes are managed with agents with a high hypoglycemic risk to achieve tight glycemic targets.[8-10] This may be explained by the nuances in managing older adults with diabetes compared to the general population: In a survey of primary care clinicians, providers were more likely to intensify diabetes treatment despite individual patient factors warranting less stringent glycemic targets.[11] In another clinician survey, 20% of respondents reported using concrete hemoglobin

Citation:
Triantafylidis LK, Phillips SC, Hawley CE, Schwartz AW. Finding the Sweet Spot: an interactive workshop on diabetes management in older adults. *MedEdPORTAL*. 2019;15:10845.
https://doi.org/10.15766/mep_2374-8265.10845

Copyright © 2019 Triantafylidis et al. This is an open-access publication distributed under the terms of the Creative Commons Attribution license.

A1c (HbA1c) thresholds rather than individualized glycemic goals, despite recommendations from the ADA and AGS to individualize.[12]

Several modules in *MedEdPORTAL* address aspects of diabetes care, including a primer for first-year medical residents; pharmacotherapy considerations using team-based learning; and a module related to the diagnosis, prevention, and goals of therapy.[13-15] There are also modules addressing aspects of caring for older adults to help support clinical decision-making.[16-17] Our goal was to develop a workshop that linked these two very important topics to provide a framework for how to manage an older adult patient with diabetes. Although guidelines exist to support managing diabetes in older adult populations, there is a need for additional training in this area, as evidenced by the lack of prescriber confidence and inappropriate use of high-risk diabetes medications in older adults.[3-10]

To improve clinician confidence and knowledge in caring for older adults with diabetes in an individualized manner, we created Finding the Sweet Spot, a 1-hour educational workshop introducing a five-step framework for diabetes management in older adults. Sessions were conducted for learners in medicine and pharmacy, using guided case examples and a clinical tool kit to serve as a point-of-care reference for safe and individualized management of older adults with diabetes. Our target audience was any clinician providing care for older adults with diabetes.

Methods

We developed Finding the Sweet Spot for trainees in medicine and pharmacy (Table 1) to improve clinician confidence and knowledge in caring for older adults with diabetes in an individualized manner. Combining the most up-to-date ADA and AGS guidelines, we created an interactive presentation introducing a five-step framework for geriatric diabetes management. The presentation was accompanied by both guided case examples and a clinical tool kit to be used as a point-of-care reference for clinicians. Key components of the session were (1) the safety and efficacy characteristics of specific antihyperglycemic agents, (2) the development of individualized treatment goals, and (3) the implementation of geriatric prescribing principles, considering the risks and benefits of certain medication regimens for older adults with diabetes. During the pilot period from January 2018 to June 2018, the presentation and clinical tool kit were developed, provided to interprofessional groups of learners, and continuously revised based on participant feedback until the final version was established and delivered to learners in medicine and pharmacy. This project was reviewed and deemed to be quality improvement by the Boston VA Medical Center Research and Development Committee and was overseen as such.

Table 1. Workshop Attendance by Profession and Designation (*N* = 30)

Profession and Designation	No. (%)
Medicine	21 (70)
Geriatric medicine fellow	6 (20)
Internal/family medicine resident	14 (47)
Medical student	1 (3)
Pharmacy	9 (30)
Pharmacy resident	6 (20)
Pharmacy student	3 (10)

The target audience for Finding the Sweet Spot included clinicians and trainees responsible for the prescription and management of medications for older adults with diabetes. Each session was delivered to five to 10 different learners to allow for interactive discussion of patient cases. Sessions were led twice per month from July to December 2018. The full duration of a Finding the Sweet Spot session was 60 minutes: PowerPoint presentation and cases (40 minutes), session conclusions (5 minutes), and audience questions (15 minutes). A small conference room with computer and audiovisual connectivity was utilized for the sessions. Learners were assessed for self-efficacy and knowledge using pre- and postsurvey assessments (Appendices A and H). Knowledge questions were derived based on key approaches to diabetes management in older adults specified in the ADA and AGS guidelines.[3,4] The postsurvey also included an open-ended response section for formative workshop feedback. The pre- and postsurvey answer keys are included in Appendix I.

There were two roles within the workshop session: the facilitator and the learners. At our institution, facilitators were two clinical pharmacists and a PGY 2 geriatrics pharmacy resident, although any clinician responsible for the prescription and management of medications for older adults with diabetes can serve as a facilitator. Facilitators were asked to review all materials (Appendices A-I) prior to the session. No formal facilitator training was required; the facilitator guide included as notes to the slides in Appendix B was intended to be used without the need for additional training. Facilitators were instructed to review the notes section of the PowerPoint presentation (Appendix B), which included a script for each

Copyright © 2019 Triantafylidis et al. This is an open-access publication distributed under the terms of the Creative Commons Attribution license.

slide. Facilitators were provided with the opportunity to ask creators of Finding the Sweet Spot questions prior to their facilitation sessions. Facilitators were instructed to print one color copy per learner of Appendices A and C-H. Appendices A and H were voluntary learner pre- and postsurveys, and Appendices C-G constituted the learner clinical tool kit for the session.

There was no prerequisite knowledge required by learners for this educational workshop, attendance was voluntary, and groups of learners were not intentionally coordinated based on profession. Prior to the start of the workshop, the facilitator distributed Appendices A and C-H to the learners. All learners were asked to complete the presurvey (Appendix A, optional). After the learners completed the presurvey, the facilitator oriented learners to the Finding the Sweet Spot learner clinical tool kit (Appendices C-G). The facilitator called attention to Appendix C, which contained the clinical cases used in the workshop, and Appendices D-G, which included guidance for individualized glycemic goals (Appendix D), considerations for the use of oral and injectable pharmacologic agents (Appendices E and F), and a deprescribing framework (Appendix G). After the orientation to the learner clinical tool kit, the facilitator began the PowerPoint presentation (Appendix B). Active learner participation was encouraged throughout the presentation and was required during the clinical cases. After completion of the PowerPoint presentation, the facilitator opened the floor to audience questions and discussion. At the close of the session, the learners were asked to complete a postsurvey (Appendix H, optional).

Results

A total of 30 learners participated in the Finding the Sweet Spot workshop, including medical students, residents, and geriatric medicine fellows (70%) and pharmacy students and residents (30%). Workshop sessions were held over a 6-month period, and there were six sessions in total. The profession and designation of each learner are specified above in Table 1. Groups of trainees rotating through the outpatient geriatrics and primary care clinics, which included students, residents, fellows, and practicing clinicians, were offered the opportunity to participate. At our institution, facilitators were two pharmacists and a geriatrics-trained PGY 2 pharmacy resident.

We assessed the change in learner self-efficacy using pre/post self-assessment surveys (Appendices A and H). Results of the pre- and postsurvey self-efficacy measures are described in Table 2. Self-efficacy was assessed via a 5-point Likert scale (1 = *not at all confident*, 5 = *extremely confident*). In our analysis, learners were classified as confident if they selected 4 (*somewhat confident*) or 5 (*extremely confident*); all other responses were classified as not confident. The percentage of confident learners increased from an average of 55% to 97% ($p < .05$) after the workshop. Learners demonstrated a statistically significant improvement in self-efficacy in all four domains of geriatric diabetes management assessed ($p < .05$).

Learner knowledge was assessed in the pre- and postsurveys via multiple-choice questions related to the three learning objectives. The presurvey contained five knowledge-based questions that were repeated on the postsurvey for comparison purposes. Results of the pre- and postsurvey knowledge-based questions are described in Table 3. The overall average score for knowledge questions showed a significant improvement; on average, learners showed an increase in knowledge of 19% ($p < .05$).

Of the 30 learners participating in the workshop, 20 completed the postsurvey open-ended response section. This section included the following items: (1) List one thing you liked, (2) list one thing you would suggest to improve, (3) list one thing you learned, and (4) other comments/questions. Learner responses were reviewed collectively to assess for trends, with examples of positive feedback presented below, arranged by theme:

Table 2. Self-Efficacy Results, Pre- Versus Postintervention (N = 30)

Self-Efficacy Evaluation[a]	Presurvey: No. (%) of Confident Learners	Postsurvey: No. (%) of Confident Learners	Delta	*p*
Identify patients who are at an increased risk for hypoglycemia	20 (67)	30 (100)	33%	<.05
Choose an appropriate hemoglobin A1c and blood glucose goal for an older adult	24 (80)	29 (97)	17%	<.05
Determine which diabetic medication is least likely to cause hypoglycemia	16 (53)	29 (97)	44%	<.05
Deprescribe a diabetic regimen to minimize the risk of adverse drug side effects	6 (20)	28 (93)	73%	<.05
Overall average	55%	97%	42%	<.05

[a]Self-efficacy was assessed via a 5-point Likert scale (1 = *not at all confident*, 2 = *not confident*, 3 = *neutral*, 4 = *somewhat confident*, 5 = *extremely confident*); learners were qualified as confident if their response was 4 or 5.

Copyright © 2019 Triantafylidis et al. This is an open-access publication distributed under the terms of the Creative Commons Attribution license.

Table 3. Knowledge Results, Pre- Versus Postintervention ($N = 30$)

Knowledge Evaluation[a]	Presurvey: No. (%) of Correct Answers	Postsurvey: No. (%) of Correct Answers	Delta	p
Objective 1: List safety and efficacy characteristics of antihyperglycemic agents				
Question: hypoglycemic risk of oral agents	15 (50)	27 (90)	40%	<.05
Question: hypoglycemic risk of insulins	23 (77)	25 (83)	6%	.52
Objective 2: Develop individualized glycemic goals for older adults with diabetes				
Question: case example to individualize A1c goal	16 (53)	24 (80)	24%	<.05
Objective 3: Implement geriatric prescribing principles for antihyperglycemic agents				
Question: case example to prescribe diabetes medications	19 (63)	24 (80)	17%	.15
Question: case example to deprescribe diabetes medications	19 (63)	21 (70)	7%	.58
Overall average	61%	81%	19%	<.05

[a]Knowledge was assessed via multiple-choice questions with four answer choice options and one correct answer.

- Tool kit (12 of 20 learners [60%] responded):
  - "I liked the presentation and found the handouts [tool kit] very useful, I will use these during clinical practice"—pharmacy resident.
  - "I liked the colorful, organized handouts [tool kit], packed with useful information"—medical resident.
  - "Great visuals which brought together this stepwise approach"—geriatric medicine fellow.
- Guidelines (seven of 20 learners [35%] responded):
  - "Use of a systematic, stepwise approach to geriatric diabetes management"—medical resident.
  - "Guidance for how to choose an individualized A1C goal for a patient"—medical resident.
  - "Choosing an antidiabetic agent that is least likely to cause hypoglycemia"—geriatric medicine fellow.
- Interactive cases (six of 20 learners [30%] responded):
  - "Very practical cases that demonstrated strategies I can use immediately in my practice"—medical resident.
  - "Very interactive cases that covered a high yield topic"—geriatric medicine fellow.

Only four learners offered suggestions for improvement, indicating that more cases would have been helpful (noted four times) and that the learner clinical tool kit could be better coordinated with the slides (noted one time), which we address in the Discussion.

Discussion

Our Finding the Sweet Spot workshop improved clinician self-efficacy and addressed previously published gaps in clinician knowledge of diabetes management for older adults. The interactive, case-based nature of this presentation allowed for a hands-on approach to improve learner confidence and knowledge in adhering to current guidelines for geriatric diabetes management. The small-group sessions of five to 10 learners allowed for interactive discussion. The shorter duration of the workshop (1 hour) allowed for ease of administration and coordination. Facilitation required little preparation time, and facilitators were able to effectively present the educational materials using the facilitator guide included in the PowerPoint presentation.

We expected that presurvey self-efficacy ratings would be relatively low related to choosing individualized HbA1c goals, identifying medications with a high risk for hypoglycemia, and deprescribing medications in older adults with diabetes. Current literature shows that clinicians treating older adults with diabetes are not confident and often are not adherent to current guidelines, which recommend individualized glycemic targets and medications with low hypoglycemia risk.[3,4,8-12] In Finding the Sweet Spot, we provided learners with a clinical tool kit that summarized safe prescribing practices, HbA1c goal setting, and deprescribing according to current guidelines.[3,4,18,19] The interactive teaching method and associated tool kit led to a statistically significant increase in learner knowledge and self-efficacy. It is our hope that beyond this session, learners will use the clinical tool kit to align their future prescribing and management practices with current guidelines for safely managing their older patients with diabetes. Although previous educational modules have been developed to promote education surrounding diabetes management,[13-17] education promoting individualized diabetes management in older adults is lacking, and guidelines rely heavily on expert opinion.[20]

Positive learner feedback indicated that this workshop was well received, appreciated, and useful for attendees. The majority of learners valued the clinical tool kit, guidance for how to manage older adults with diabetes, and clinical cases to apply knowledge learned. Few participants responded with suggestions for how to improve the workshop or negative feedback. One learner expressed a need for clarification about which parts of the

Copyright © 2019 Triantafylidis et al. This is an open-access publication distributed under the terms of the Creative Commons Attribution license.

clinical tool kit to refer to during the PowerPoint presentation. We subsequently added symbols to the slides that coordinated with the tool kit to better allow facilitators and learners to refer to the tool kit while actively participating. Other learners suggested additional clinical cases; to accommodate this, the duration of the workshop can be extended to allow for additional clinical cases and more application-based learning.

The main limitation to our activity is that it was developed based on current ADA and AGS guidelines. There is potential for newer antidiabetic agents and diabetes guidelines to be developed, prompting a need for the clinical tool kit to be updated. However, the general content of this workshop related to individualizing HbA1c goals and prescribing principles to consider in older adults with diabetes will remain relevant, and this information is unlikely to change over time with guideline updates, as the AGS Guidelines for Improving the Care of Older Adults With Diabetes Mellitus have not been updated since 2013 and the most recent ADA updates have continued to align with the AGS.

An additional limitation is that neither delayed recall nor practice change related to this workshop was assessed. The tool kit was developed as a point-of-care reference for clinicians with the hope that its use would persist past the duration of the workshop and within clinical practice. However, it is unknown how this clinical tool kit was used following the workshop or if the improvement in self-efficacy and knowledge was sustained. Anecdotally, several participants have reported that they continue to refer to the tool kit resources months after the workshop in various settings, including inpatient and outpatient practice. A final limitation is that facilitator perspectives were not assessed during the final implementation period. During the 6-month pilot period, we received informal feedback from both learners and facilitators to help guide real-time revisions. It would have been useful to have performed formal surveys of the facilitators to evaluate their general perceptions of how the workshop went, the burden of time spent, and/or the value of benefit perceived.

Future opportunities to strengthen this workshop include widening the target audience to include nurse practitioners, physician assistants, nurses, and other health care professionals responsible for the prescription and management of medications for diabetes in older adults. Sessions could be performed with larger groups of interprofessional learners and include segments breaking out into smaller groups for case-based discussions to encourage interprofessional collaboration. Additionally, there could be considerations for expanding the duration of the workshop and including other information related to diabetes management, such as cardiovascular and renal benefits of newer diabetes agents and compelling indications for use. However, due to the time constraints and the overall goal of the workshop, we chose not to include this information.

Conclusion

Our Finding the Sweet Spot workshop was an effective tool to educate learners in medicine and pharmacy on safe, individualized management of diabetes in older adults. Statistically significant changes in self-efficacy and knowledge among learners indicate that this interactive, case-based workshop improves confidence and skills related to medication management in older adults with diabetes.

Appendices

A. Presurvey.docx
B. Finding the Sweet Spot Slides.pptx
C. Finding the Sweet Spot Activity.docx
D. Considerations for A1c Targets.pptx
E. Noninsulin Pharmacologic Options.pptx
F. Insulin Pharmacologic Options.pptx
G. Approach to Prescribing and Deprescribing.pptx
H. Postsurvey.docx
I. Pre- and Postsurvey Answer Guide.docx

All appendices are peer reviewed as integral parts of the Original Publication.

Laura K. Triantafylidis, PharmD: Clinical Pharmacy Specialist, Pharmacy Department, VA Boston Healthcare System

Sarah C. Phillips, MD: Instructor, Department of Family Medicine, Boston University School of Medicine; Physician, Upham's Corner Elder Service Plan; Affiliated Fellow in Geriatrics, VA Boston Healthcare System and New England Geriatric Research Education and Clinical Center

Chelsea E. Hawley, PharmD: Clinical Pharmacist, Pharmacy Department, VA Boston Healthcare System; Advanced Fellow in Geriatrics, New England Geriatric Research Education and Clinical Center

Andrea Wershof Schwartz, MD, MPH: Associate Fellowship Director of the Harvard Multicampus Geriatrics Fellowship, VA Boston Healthcare System and New England Geriatric Research Education and Clinical Center; Assistant Professor of Medicine, Department of Medicine, Harvard Medical School

Copyright © 2019 Triantafylidis et al. This is an open-access publication distributed under the terms of the Creative Commons Attribution license.

Acknowledgments
We would like to thank our interprofessional learners: the VA Boston visiting “Geriatric Scholars,” the internal medicine residents of Brigham and Women’s Hospital, the Harvard Multicampus Geriatric Fellows, and the VA Boston Healthcare System pharmacy residents and students. We also thank our facilitator, Christopher Fagbote, PharmD. Lastly, we thank the VA Boston Pharmacy Residency Program directors John Roefaro, PharmD, FASHP, BCGP, and Bryan Wood, PharmD, for program support and identification of learners.

Disclosures
None to report.

Funding/Support
This material is the result of work supported with resources and the use of facilities at the VA Boston Healthcare System.

Prior Presentations
Triantafylidis LK, Phillips S, Hawley CE, Schwartz AW. Finding the Sweet Spot: diabetes management in older adults. Poster presented at: American Geriatrics Society Annual Scientific Meeting; May 2-4, 2019; Portland, OR.

Ethical Approval
Reported as not applicable.

Disclaimer
The contents do not represent the views of the U.S. Department of Veterans Affairs or the United States Government.

References

1. Centers for Disease Control and Prevention. *National Diabetes Statistics Report, 2017*. Atlanta, GA: Centers for Disease Control and Prevention, U.S. Department of Health and Human Services; 2017.
2. Kirkman MS, Briscoe VJ, Clark N, et al. Diabetes in older adults. *Diabetes Care*. 2012;35(12):2650-2664. https://doi.org/10.2337/dc12-1801
3. American Diabetes Association. Standards of medical care in diabetes—2019. *Diabetes Care*. 2019;42(suppl 1):S1-S193.
4. American Geriatrics Society Expert Panel on the Care of Older Adults With Diabetes Mellitus. Guidelines abstracted from the American Geriatrics Society Guidelines for Improving the Care of Older Adults With Diabetes Mellitus: 2013 update. *J Am Geriatr Soc*. 2013;61(11):2020-2026. https://doi.org/10.1111/jgs.12514
5. Greenfield S, Billimek J, Pellegrini F, et al. Comorbidity affects the relationship between glycemic control and cardiovascular outcomes in diabetes: a cohort study. *Ann Intern Med*. 2009;151(12):854-860. https://doi.org/10.7326/0003-4819-151-12-200912150-00005
6. Vijan S, Sussman JB, Yudkin JS, Hayward RA. Effect of patients’ risks and preferences on health gains with plasma glucose level lowering in type 2 diabetes mellitus. *JAMA Intern Med*. 2014;174(8):1227-1234. https://doi.org/10.1001/jamainternmed.2014.2894
7. Miller ME, Bonds DE, Gerstein HC, et al; for ACCORD Investigators. The effect of baseline characteristics, glycaemia treatment approach, and glycated haemoglobin concentration on the risk of severe hypoglycaemia: post hoc epidemiological analysis of the ACCORD study. *BMJ*. 2010;340:b5444. https://doi.org/10.1136/bmj.b5444
8. Lipska KJ, Krumholz H, Soones T, Lee SJ. Polypharmacy in the aging patient: a review of glycemic control in older adults with type 2 diabetes. *JAMA*. 2016;315(10):1034-1045. https://doi.org/10.1001/jama.2016.0299
9. Lipska KJ, Ross JS, Miao Y, Shah ND, Lee SJ, Steinman MA. Potential overtreatment of diabetes mellitus in older adults with tight glycemic control. *JAMA Intern Med*. 2015;175(3):356-362. https://doi.org/10.1001/jamainternmed.2014.7345
10. Caverly TJ, Fagerlin A, Zikmund-Fisher BJ, et al. Appropriate prescribing for patients with diabetes at high risk for hypoglycemia: national survey of Veterans Affairs health care professionals. *JAMA Intern Med*. 2015;175(12):1994-1996. https://doi.org/10.1001/jamainternmed.2015.5950
11. McCreedy EM, Kane RL, Gollust SE, Shippee ND, Clark KD. Patient-centered guidelines for geriatric diabetes care: potential missed opportunities to avoid harm. *J Am Board Fam Med*. 2018;31(2):192-200. https://doi.org/10.3122/jabfm.2018.02.170141
12. Genere N, Sargis RM, Masi CM, et al. Physician perspectives on de-intensifying diabetes medications. *Medicine*. 2016;95(46):e5388. https://doi.org/10.1097/MD.0000000000005388
13. Amori RE, Simon B. A primer on diabetes mellitus: foundations for the incoming first-year resident. *MedEdPORTAL*. 2016;12:10469. https://doi.org/10.15766/mep_2374-8265.10469
14. Goedde M, Tracy P, D’Epiro G, Gilbert M. Pharmacotherapy of type 2 diabetes: team-based learning. *MedEdPORTAL*. 2016;12:10395. https://doi.org/10.15766/mep_2374-8265.10395
15. Sprabery L, George C, Brescia W. Diabetes mellitus: diagnosis, prevention and goals of therapy. *MedEdPORTAL*. 2011;7:7944. https://doi.org/10.15766/mep_2374-8265.7944
16. Phillips SC, Hawley CE, Triantafylidis LK, Schwartz AW. Geriatric 5Ms for primary care workshop. *MedEdPORTAL*. 2019;15:10814. https://doi.org/10.15766/mep_2374-8265.10814
17. Denham AC. Clinical decision-making for older patients with multiple chronic conditions: a case-based exercise for medical students. *MedEdPORTAL*. 2016;12:10477. https://doi.org/10.15766/mep_2374-8265.10477
18. Farrell B, Black C, Thompson W, et al. Deprescribing antihyperglycemic agents in older persons: evidence-based

Copyright © 2019 Triantafylidis et al. This is an open-access publication distributed under the terms of the Creative Commons Attribution license.

clinical practice guideline. *Can Fam Physician*. 2017;63(11): 832-843.

19. Scott IA, Hilmer SN, Reeve E, et al. Reducing inappropriate polypharmacy: the process of deprescribing. *JAMA Intern Med*. 2015;175(5):827-834. https://doi.org/10.1001/jamainternmed.2015.0324

20. Mooradian AD. Evidence-based management of diabetes in older adults. *Drugs Aging*. 2018;35(12):1065-1078. https://doi.org/10.1007/s40266-018-0598-3

Received: December 14, 2018
Accepted: June 24, 2019
Published: October 18, 2019

Copyright © 2019 Triantafylidis et al. This is an open-access publication distributed under the terms of the Creative Commons Attribution license.
