# Supplementary material for: Finding the Sweet Spot: An Interactive Workshop on Diabetes Management in Older Adults
Source: MedEdPORTAL. 2019 Oct 18;15:10845. doi: 10.15766/mep_2374-8265.10845 (PMC6944249; doi:10.15766/mep_2374-8265.10845)
Supplement: Supplementary file 1 — A. Presurvey.docx B. Finding the Sweet Spot Slides.pptx C. Finding the Sweet Spot Activity.docx D. Considerations for A1c Targets.pptx E. Noninsulin Pharmacologic Options.pptx F. Insulin Pharmacologic Options.pptx G. Approach to Prescribing and Deprescribing.pptx H. Postsurvey.docx I. Pre- and Postsurvey Answer Guide.docx [file mep-15-10845-s001.zip › A. Presurvey.docx]

Thank you for your participation in today’s workshop. This survey is optional. Your responses will be used to improve geriatrics teaching for trainees and professionals.

In order to anonymously link your responses to this survey with future surveys, please provide the first 2 digits of your birth month and the last four digits of your cell phone (i.e., 04-6002)

**First 2 digits of birth month:__________________ Last 4 digits of cell phone: _____________________**

**Position (circle one):** Medical resident Pharmacy resident Healthcare provider (MD,NP,PA)

Medical Student Pharmacy student Nurse

Pharmacist Other (specify):______________

**On a scale of 1 to 5, how confident are you in your ability to (1 = not at all confident; 5 = extremely confident):**

| **Identify patients who are at an increased risk for hypoglycemia** | **1** | **2** | **3** | **4** | **5** |
| --- | --- | --- | --- | --- | --- |
| **Choose an appropriate A1c and blood glucose goal for an older adult** | **1** | **2** | **3** | **4** | **5** |
| **Determine which diabetic medication is least likely to cause hypoglycemia** | **1** | **2** | **3** | **4** | **5** |
| **Deprescribe a diabetic regimen to minimize the risk for adverse drug side effects** | **1** | **2** | **3** | **4** | **5** |

**Please answer the following questions to the best of your ability (select the best answer choice):**

1. **Which of the following oral diabetes medication has the greatest risk of hypoglycemia when used alone?**
2. Glipizide
3. Glyburide
4. Metformin
5. Saxagliptin
6. **An 81 year old female with type 2 diabetes (A1C 9.7%), hypertension, hyperlipidemia, Parkinson’s disease, heart failure, acid reflux, arthritis and depression is seen in primary care. She is managed with glipizide 10 mg twice daily before meals and is unable to tolerate metformin. Recent labs show normal renal function. What is the next step in diabetes management?**
   1. Start a TZD (pioglitazone) and follow-up with weight check in 1 month
   2. Start an SGLT-2 inhibitor (empagliflozin) and follow-up with BP and BMP in 1 month
   3. Increase glipizide to 20 mg twice daily before meals
   4. Start insulin NPH 10 units twice daily
7. **Which insulin has the greatest risk for hypoglycemia when used alone?**
8. Insulin detemir
9. Insulin glargine
10. Insulin NPH
11. Insulin NPH and insulin regular 70/30
12. **An otherwise healthy 88 year old with type 2 diabetes (A1C 7%) is currently managed with metformin 1000 mg twice daily and glipizide 2.5 mg twice daily. He does not check his blood sugar and does endorse occasional dizziness. What change should you make to his regimen?**
13. Advise blood sugar checks to see if dizziness is due to hypoglycemia
14. Reduce the metformin as it is causing hypoglycemia and dizziness
15. Increase metformin to 2000 mg twice daily and stop glipizide
16. Stop glipizide and follow-up in 3 months to see if his A1C is at goal.
17. **A 75 year old nursing-home patient with type 2 diabetes is noted to have frequent hypoglycemia. She currently receives insulin NPH 20 units twice daily. Other relevant medical history includes severe cognitive impairment, oxygen-dependent chronic obstructive pulmonary disease, hypertension, and depression. What is your next step for management?**
18. Reduce insulin NPH to 20 units once daily
19. Stop insulin NPH, start a sliding scale correctional insulin
20. Change insulin NPH to a once daily long acting basal insulin (insulin glargine)
21. Reduce insulin NPH to 10 units twice daily
