# Supplementary material for: Finding the Sweet Spot: An Interactive Workshop on Diabetes Management in Older Adults
Source: MedEdPORTAL. 2019 Oct 18;15:10845. doi: 10.15766/mep_2374-8265.10845 (PMC6944249; doi:10.15766/mep_2374-8265.10845)
Supplement: Supplementary file 1 — A. Presurvey.docx B. Finding the Sweet Spot Slides.pptx C. Finding the Sweet Spot Activity.docx D. Considerations for A1c Targets.pptx E. Noninsulin Pharmacologic Options.pptx F. Insulin Pharmacologic Options.pptx G. Approach to Prescribing and Deprescribing.pptx H. Postsurvey.docx I. Pre- and Postsurvey Answer Guide.docx [file mep-15-10845-s001.zip › B. Finding the Sweet Spot Slides.pptx]

## Slide 1
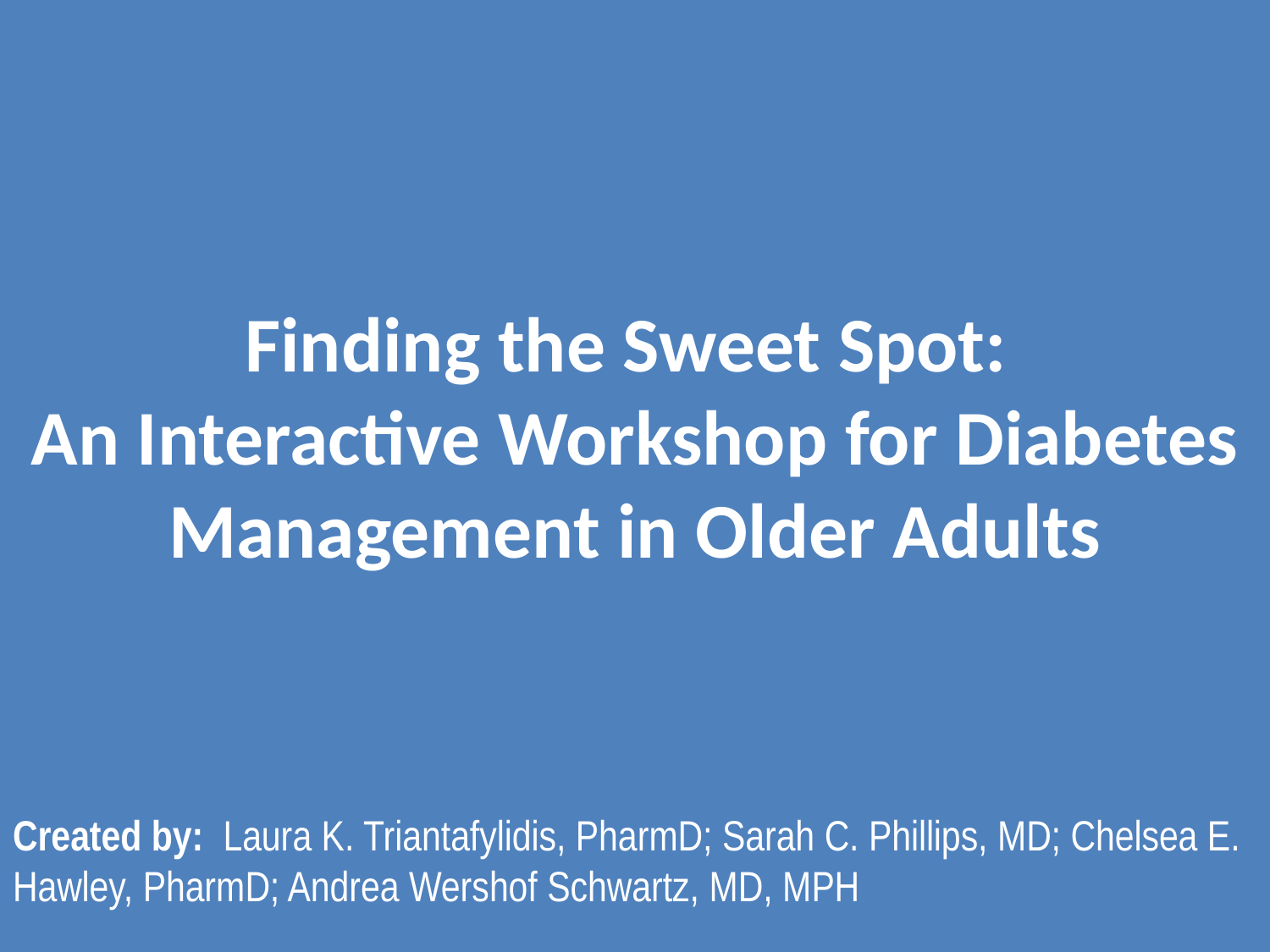

# Finding the Sweet Spot: An Interactive Workshop for Diabetes Management in Older Adults
Created by: Laura K. Triantafylidis, PharmD; Sarah C. Phillips, MD; Chelsea E. Hawley, PharmD; Andrea Wershof Schwartz, MD, MPH

## Slide 2
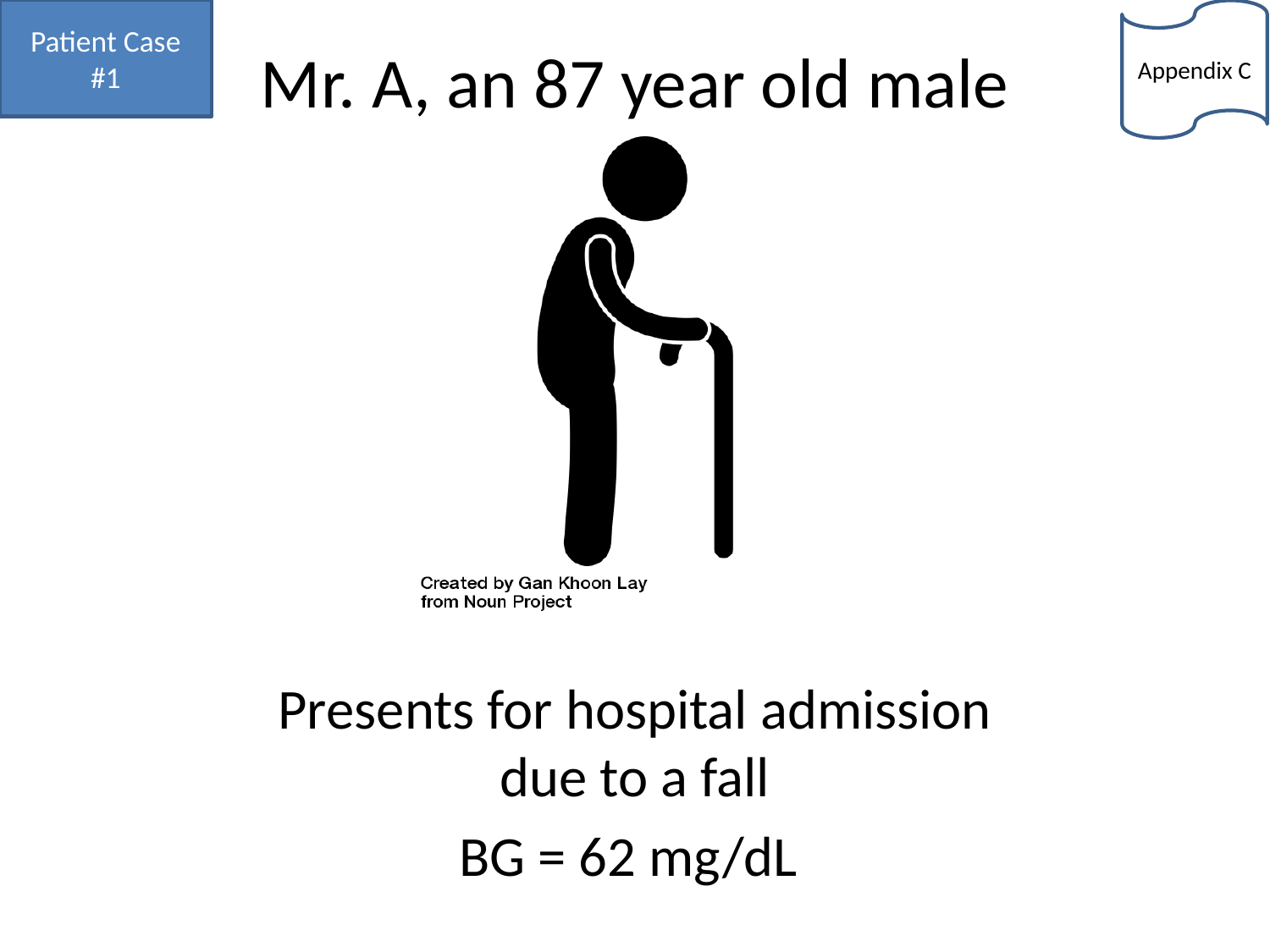

Patient Case #1
# Mr. A, an 87 year old male
Appendix C
Presents for hospital admission due to a fall
BG = 62 mg/dL

## Slide 3
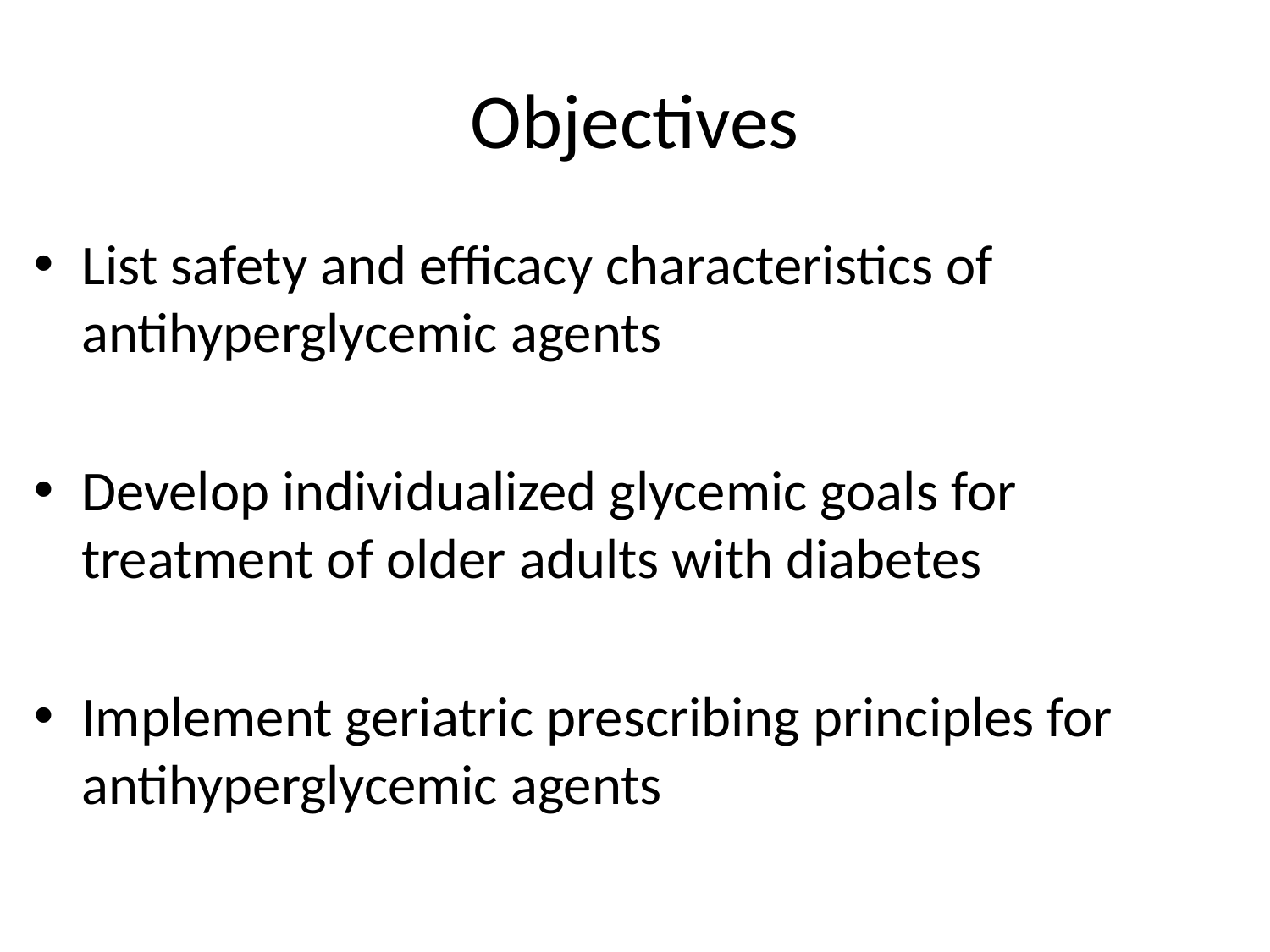

# Objectives
List safety and efficacy characteristics of antihyperglycemic agents
Develop individualized glycemic goals for treatment of older adults with diabetes
Implement geriatric prescribing principles for antihyperglycemic agents

## Slide 4
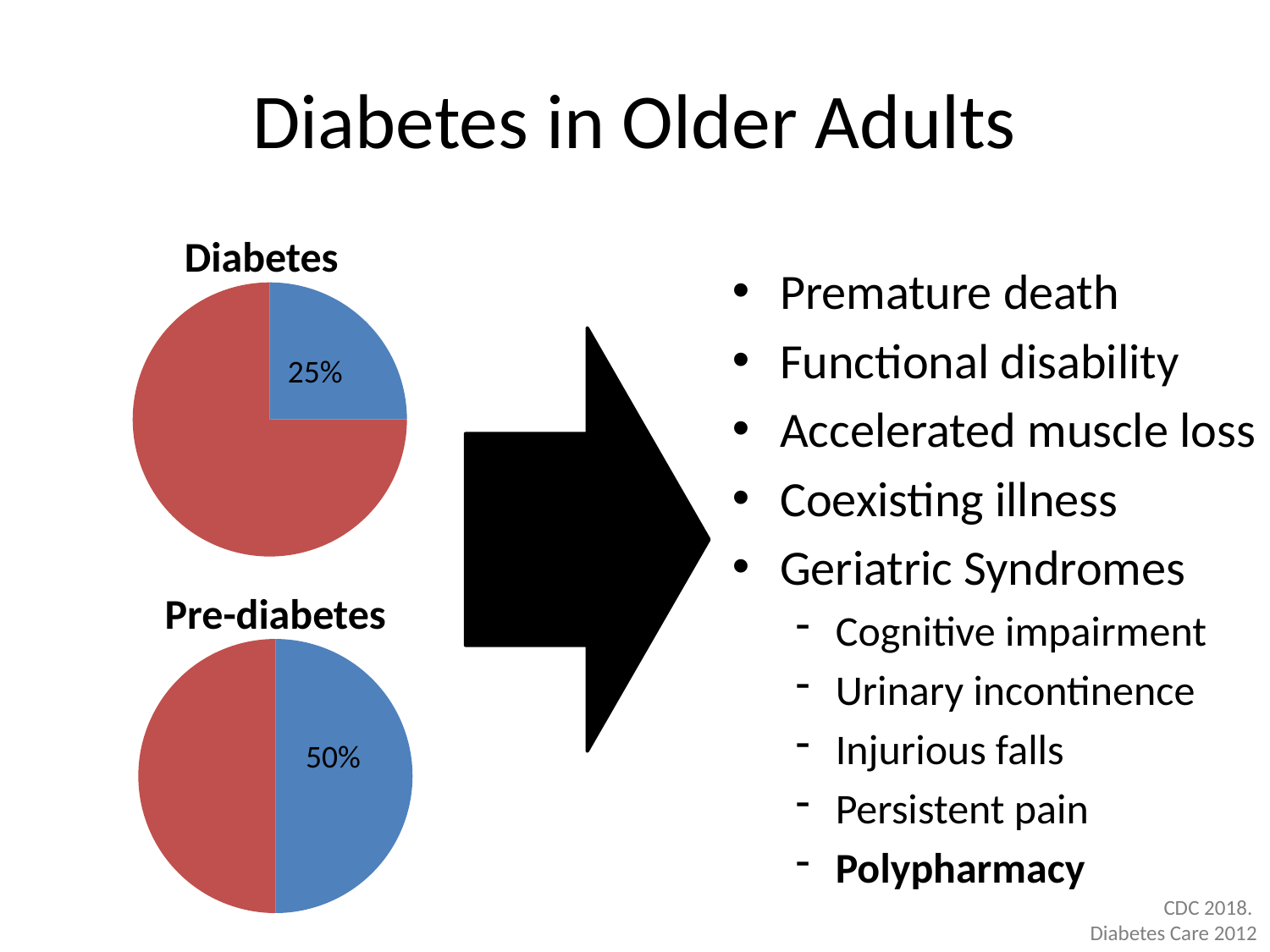

# Diabetes in Older Adults
Diabetes
Premature death
Functional disability
Accelerated muscle loss
Coexisting illness
Geriatric Syndromes
Cognitive impairment
Urinary incontinence
Injurious falls
Persistent pain
Polypharmacy
### Chart
| Category | Sales |
|---|---|
| 1st Qtr | 1.0 |
| 2nd Qtr | 3.0 |
25%
Pre-diabetes
### Chart
| Category | Sales |
|---|---|
| 1st Qtr | 2.0 |
| 2nd Qtr | 2.0 |50%
CDC 2018.
Diabetes Care 2012

## Slide 5
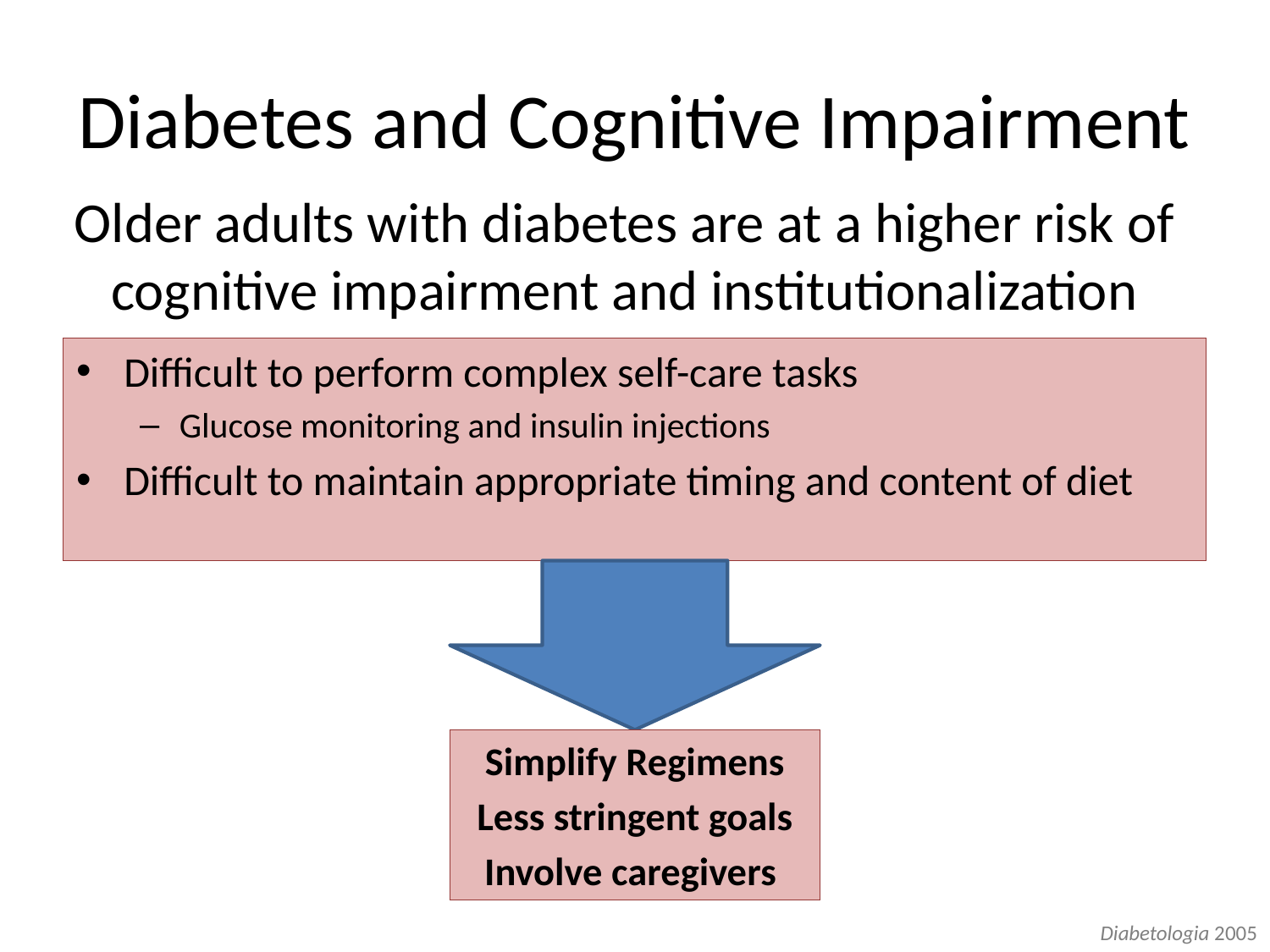

# Diabetes and Cognitive Impairment
Older adults with diabetes are at a higher risk of cognitive impairment and institutionalization
Difficult to perform complex self-care tasks
Glucose monitoring and insulin injections
Difficult to maintain appropriate timing and content of diet
Simplify Regimens
Less stringent goals
Involve caregivers
Diabetologia 2005

## Slide 6
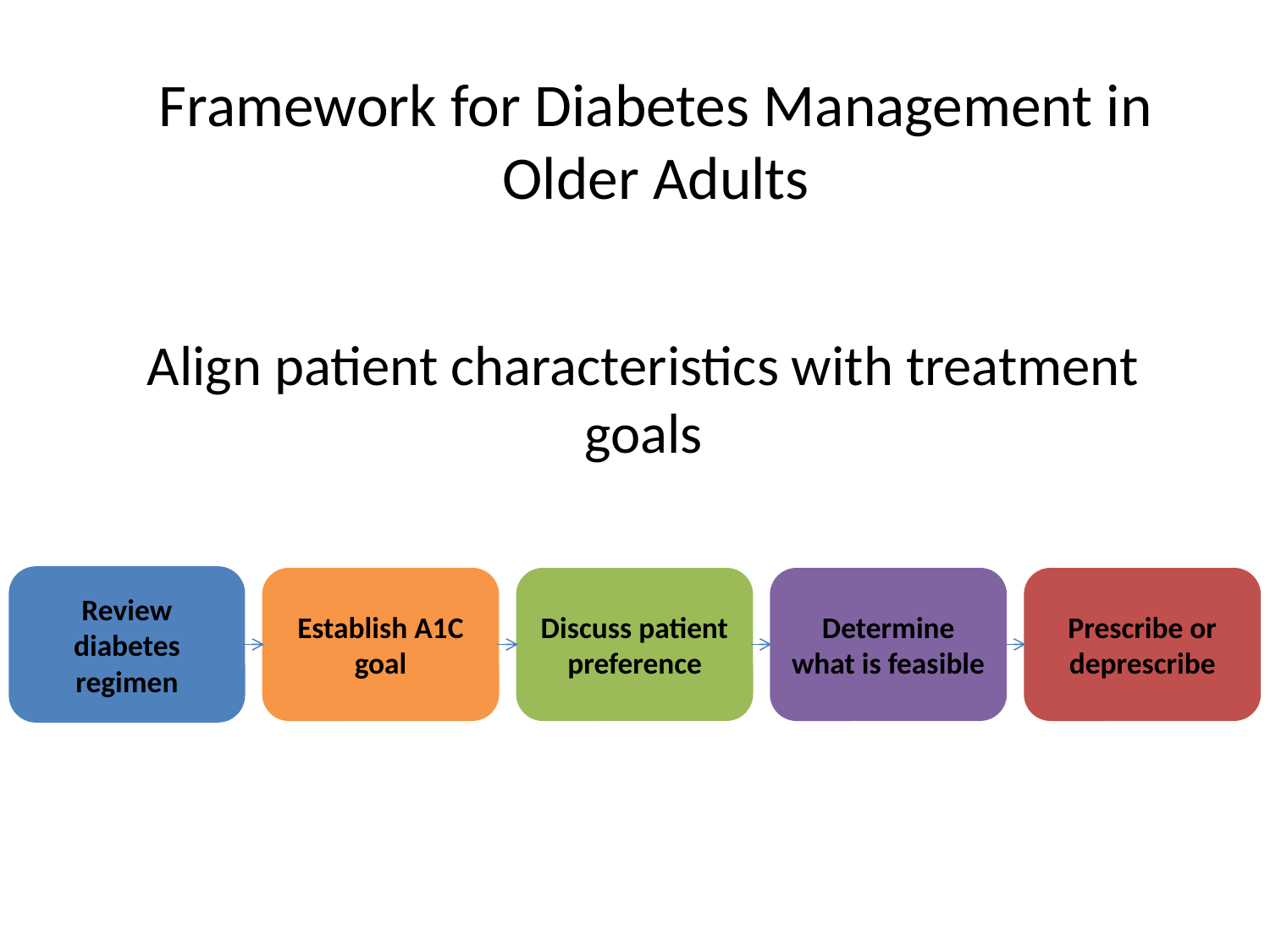

Framework for Diabetes Management in Older Adults
Align patient characteristics with treatment goals
Review diabetes regimen
Establish A1C goal
Discuss patient preference
Determine what is feasible
Prescribe or deprescribe

## Slide 7
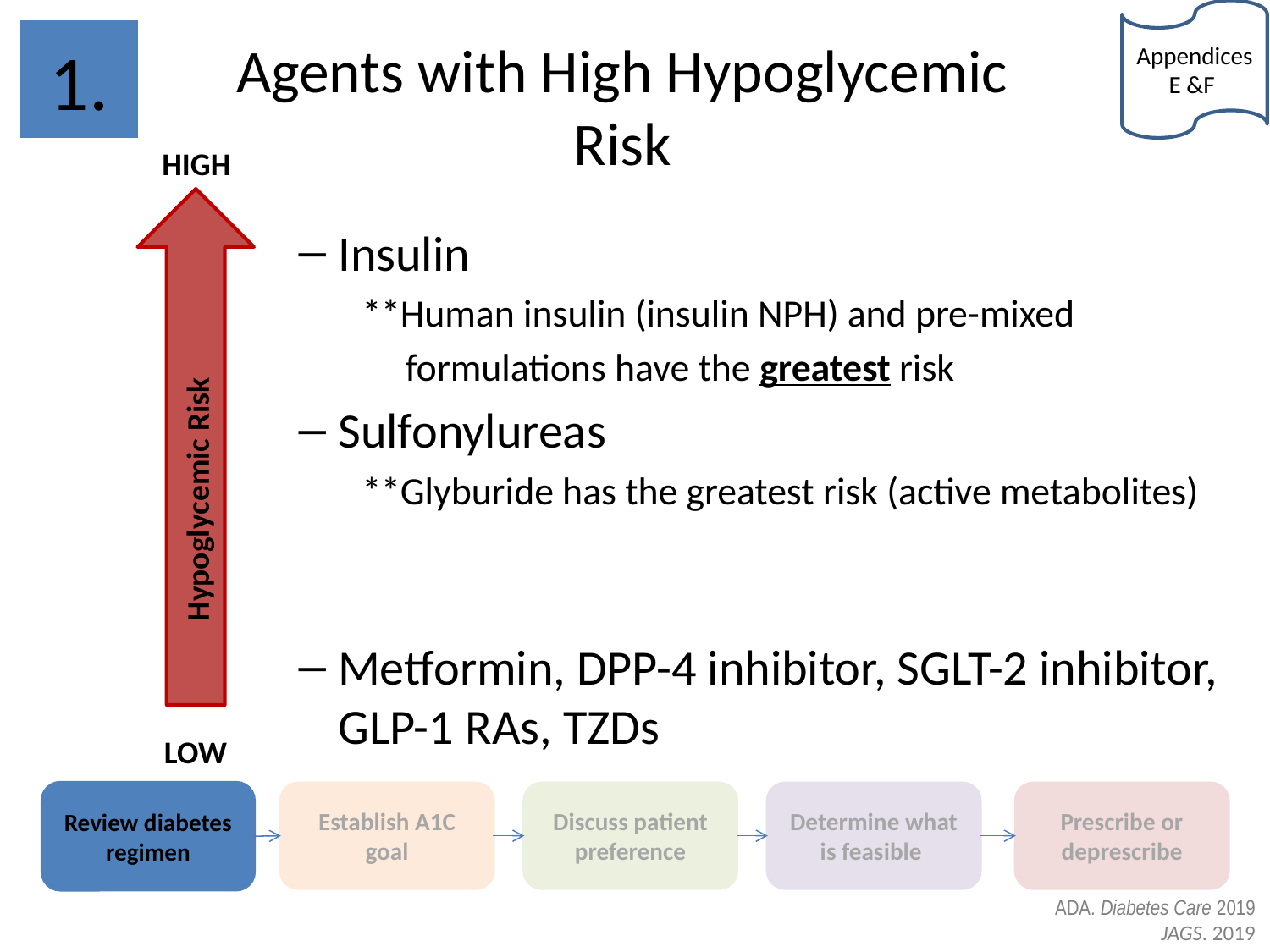

Appendices
E &F
1.
# Agents with High Hypoglycemic Risk
HIGH
Insulin
**Human insulin (insulin NPH) and pre-mixed
 formulations have the greatest risk
Sulfonylureas
**Glyburide has the greatest risk (active metabolites)
Metformin, DPP-4 inhibitor, SGLT-2 inhibitor, GLP-1 RAs, TZDs
Hypoglycemic Risk
LOW
Review diabetes regimen
Establish A1C goal
Discuss patient preference
Determine what is feasible
Prescribe or deprescribe
ADA. Diabetes Care 2019
 JAGS. 2019

## Slide 8
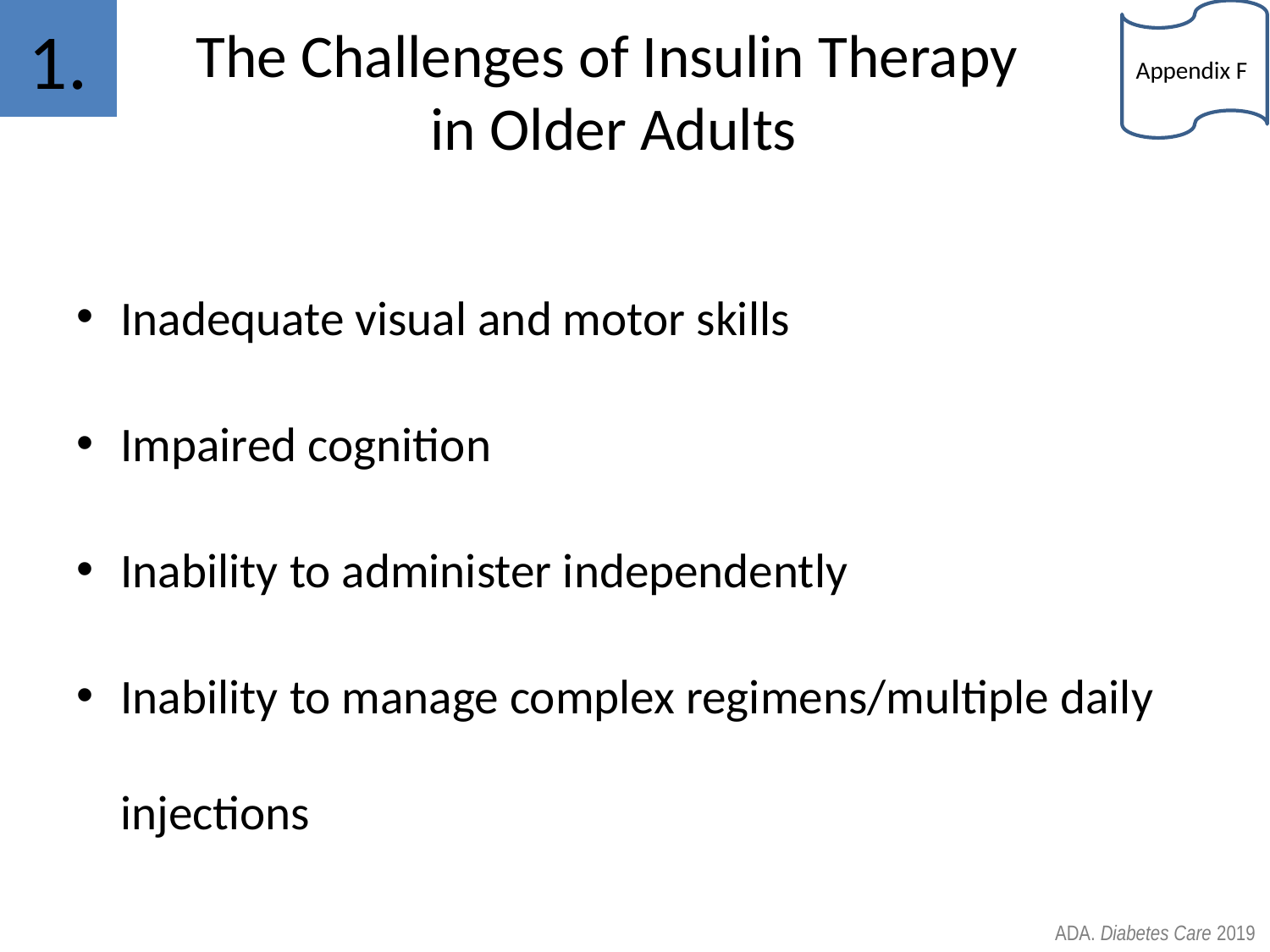

1.
Appendix F
# The Challenges of Insulin Therapy in Older Adults
Inadequate visual and motor skills
Impaired cognition
Inability to administer independently
Inability to manage complex regimens/multiple daily injections
ADA. Diabetes Care 2019

## Slide 9
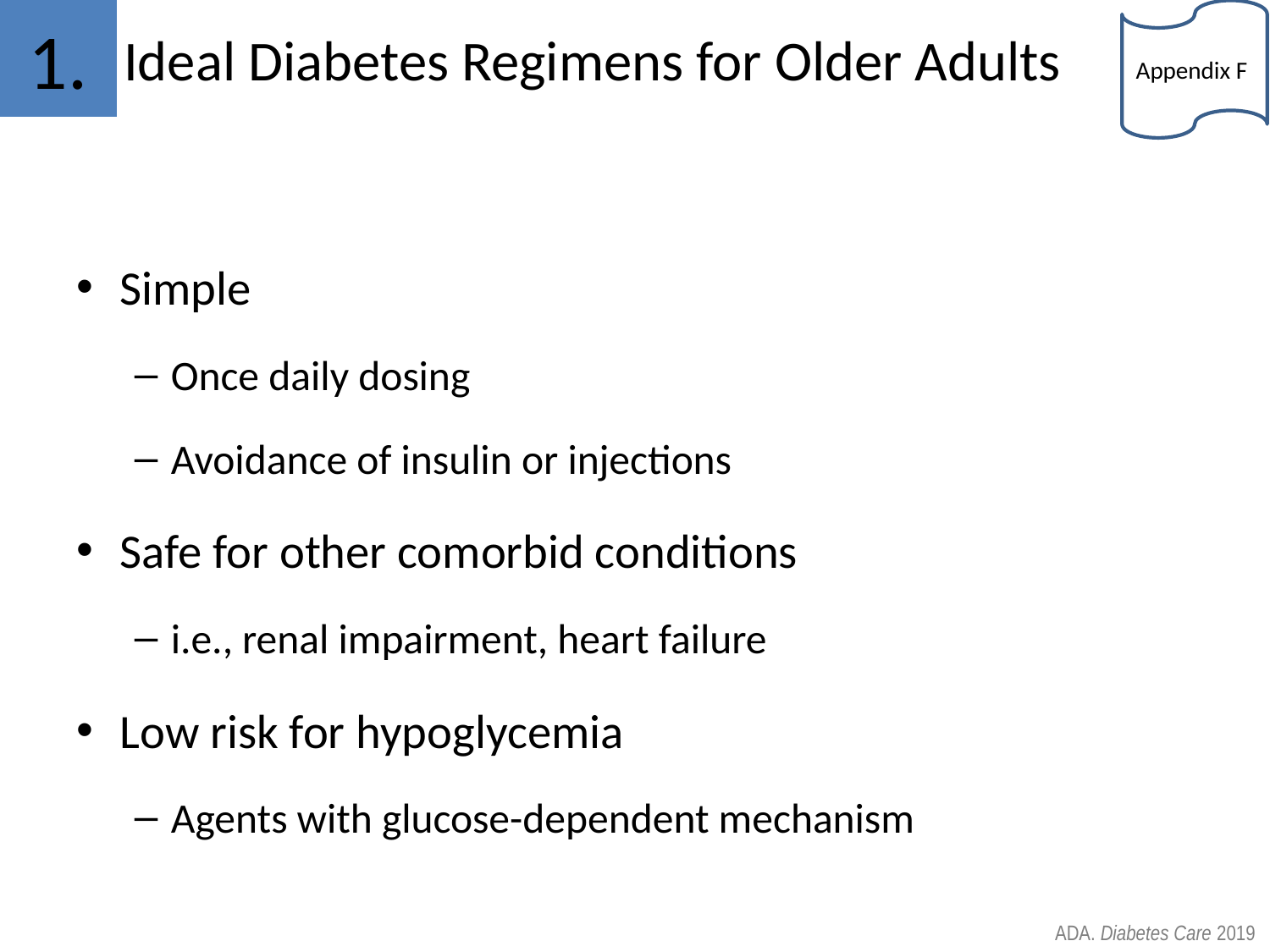

1.
# Ideal Diabetes Regimens for Older Adults
Appendix F
Simple
Once daily dosing
Avoidance of insulin or injections
Safe for other comorbid conditions
i.e., renal impairment, heart failure
Low risk for hypoglycemia
Agents with glucose-dependent mechanism
ADA. Diabetes Care 2019

## Slide 10
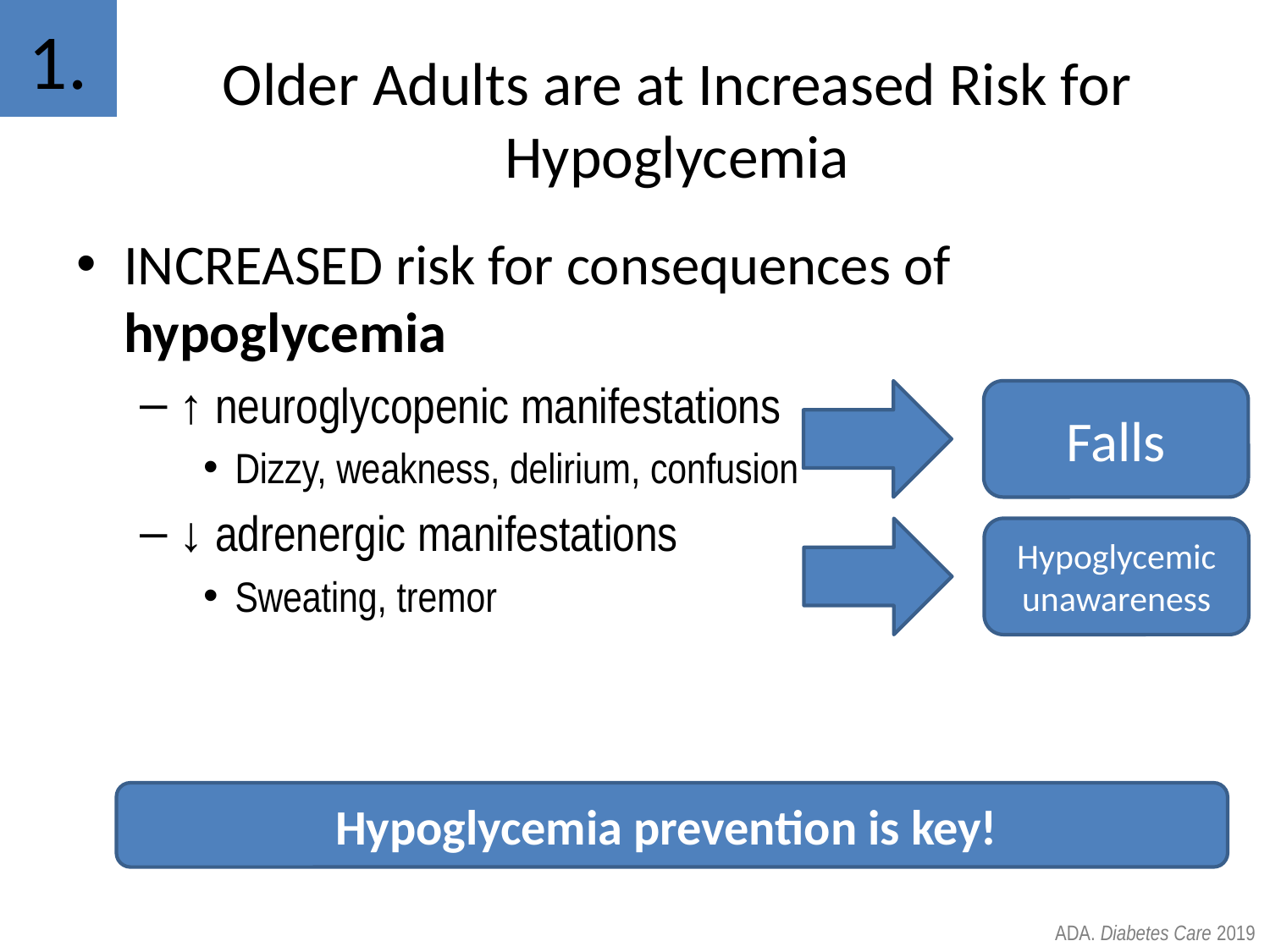

1.
# Older Adults are at Increased Risk for Hypoglycemia
INCREASED risk for consequences of hypoglycemia
↑ neuroglycopenic manifestations
Dizzy, weakness, delirium, confusion
↓ adrenergic manifestations
Sweating, tremor
Falls
Hypoglycemic unawareness
Hypoglycemia prevention is key!
ADA. Diabetes Care 2019

## Slide 11
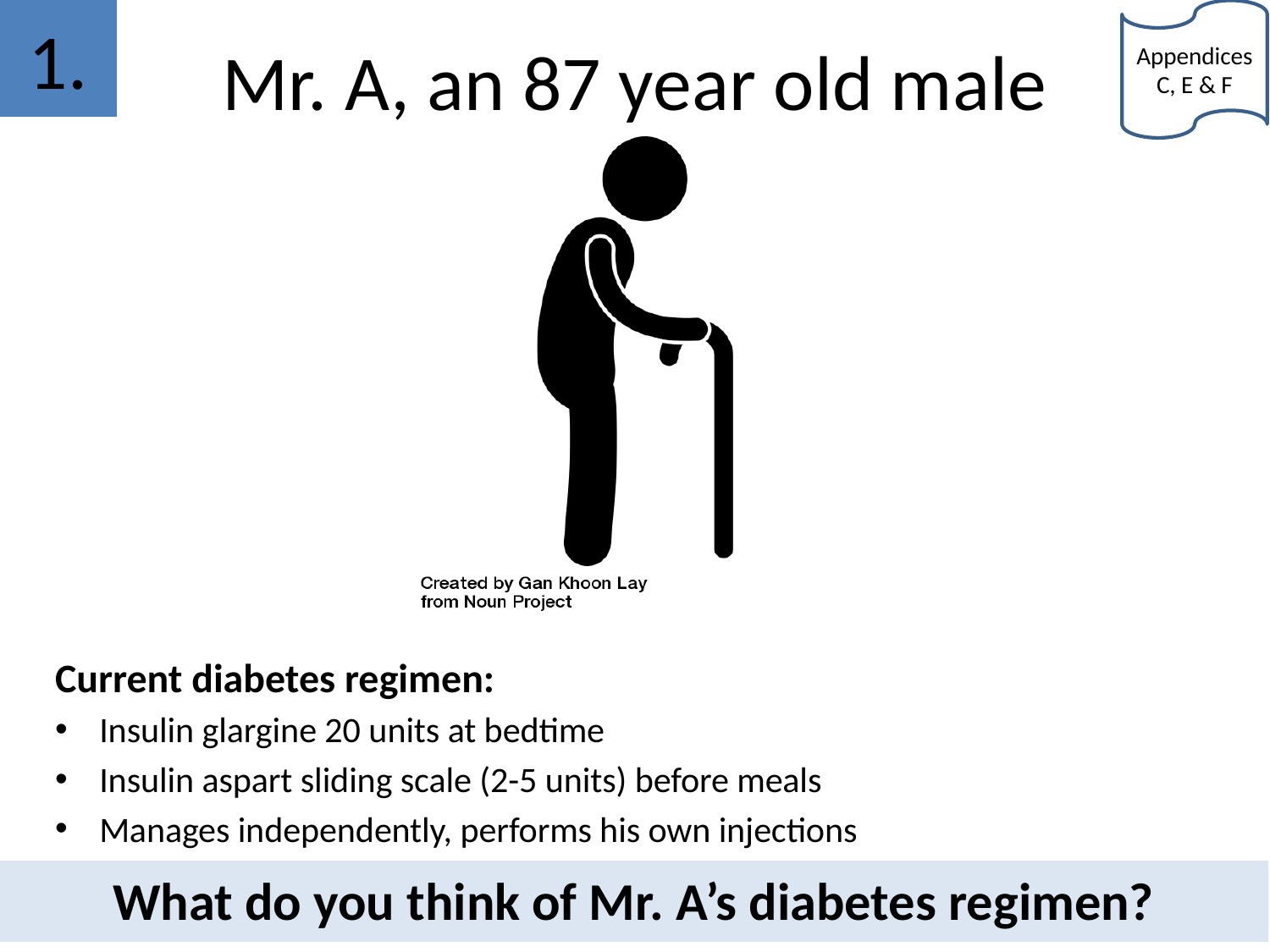

1.
# Mr. A, an 87 year old male
AppendicesC, E & F
Current diabetes regimen:
Insulin glargine 20 units at bedtime
Insulin aspart sliding scale (2-5 units) before meals
Manages independently, performs his own injections
What do you think of Mr. A’s diabetes regimen?

## Slide 12
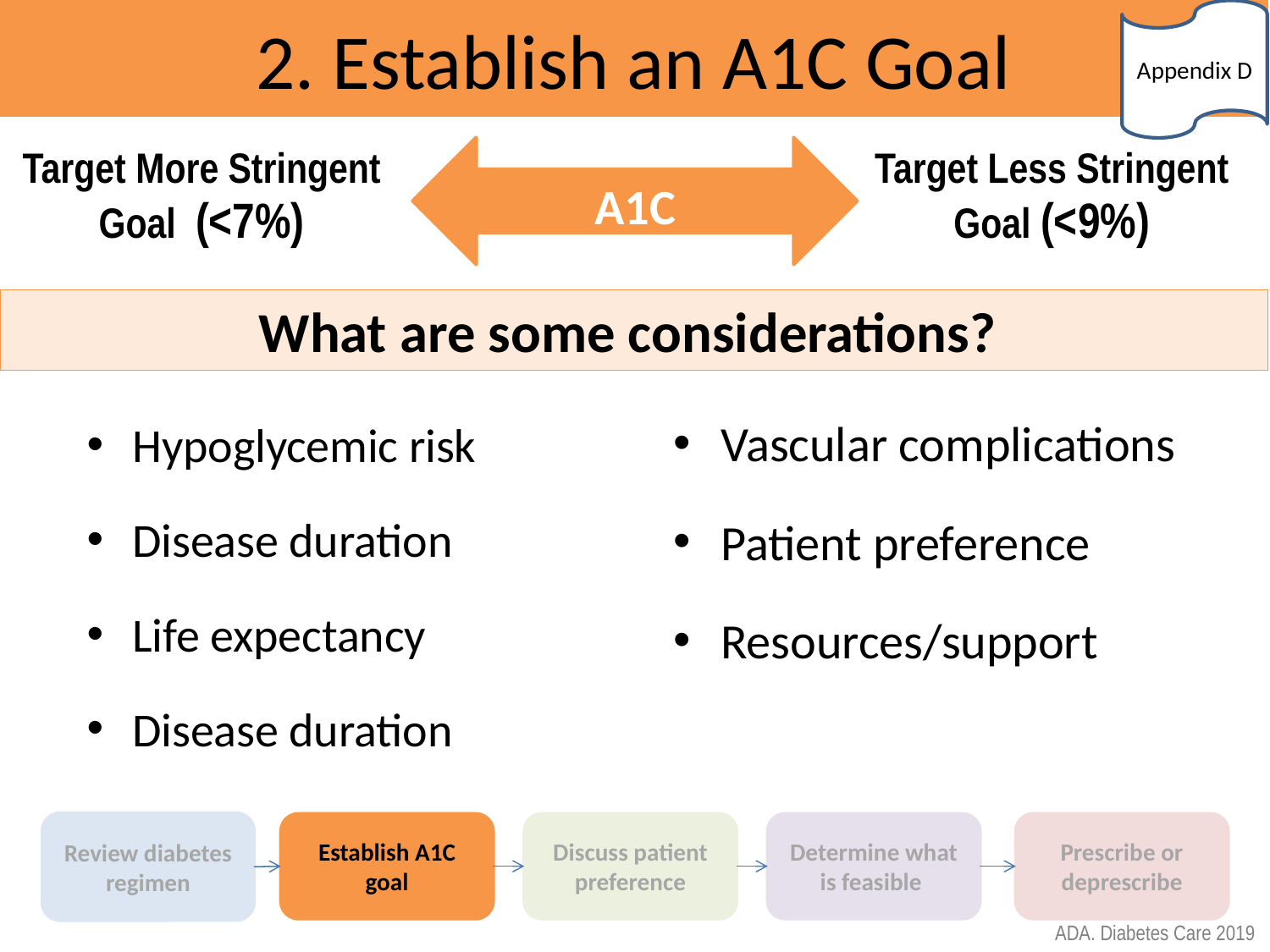

2. Establish an A1C Goal
Appendix D
| Target More Stringent Goal (<7%) | | | Target Less Stringent Goal (<9%) |
| --- | --- | --- | --- |
A1C
What are some considerations?
Vascular complications
Patient preference
Resources/support
Hypoglycemic risk
Disease duration
Life expectancy
Disease duration
Review diabetes regimen
Establish A1C goal
Discuss patient preference
Determine what is feasible
Prescribe or deprescribe
ADA. Diabetes Care 2019

## Slide 13
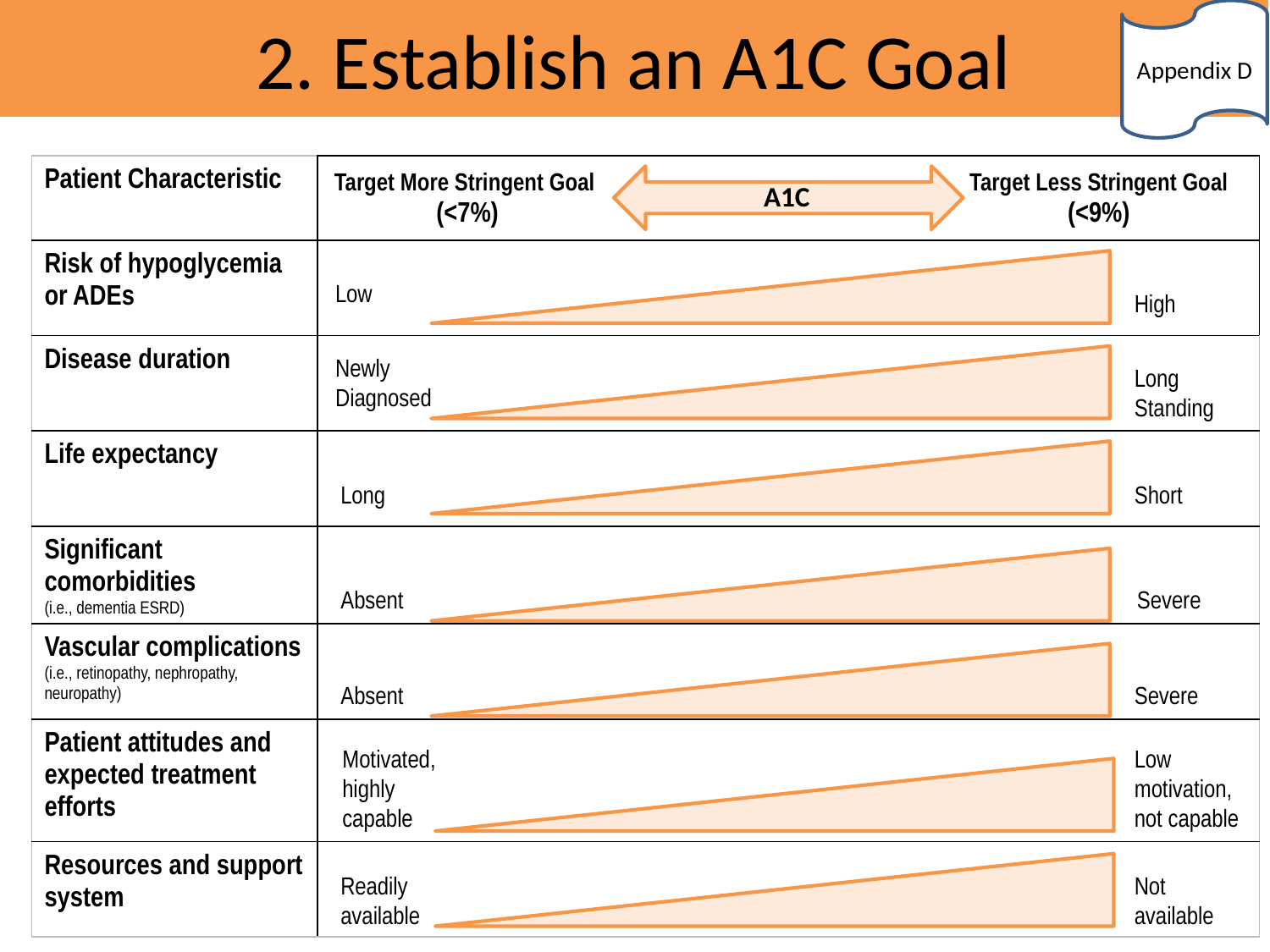

2. Establish an A1C Goal
Appendix D
| Patient Characteristic | Target More Stringent Goal (<7%) | | | Target Less Stringent Goal (<9%) |
| --- | --- | --- | --- | --- |
| Risk of hypoglycemia or ADEs | | | | |
| Disease duration | | | | |
| Life expectancy | | | | |
| Significant comorbidities (i.e., dementia ESRD) | | | | |
| Vascular complications (i.e., retinopathy, nephropathy, neuropathy) | | | | |
| Patient attitudes and expected treatment efforts | | | | |
| Resources and support system | | | | |
A1C
Low
High
Newly
Diagnosed
Long
Standing
Long
Short
Severe
Absent
Absent
Severe
Motivated,
highly
capable
Low motivation, not capable
Readily available
Not
available

## Slide 14
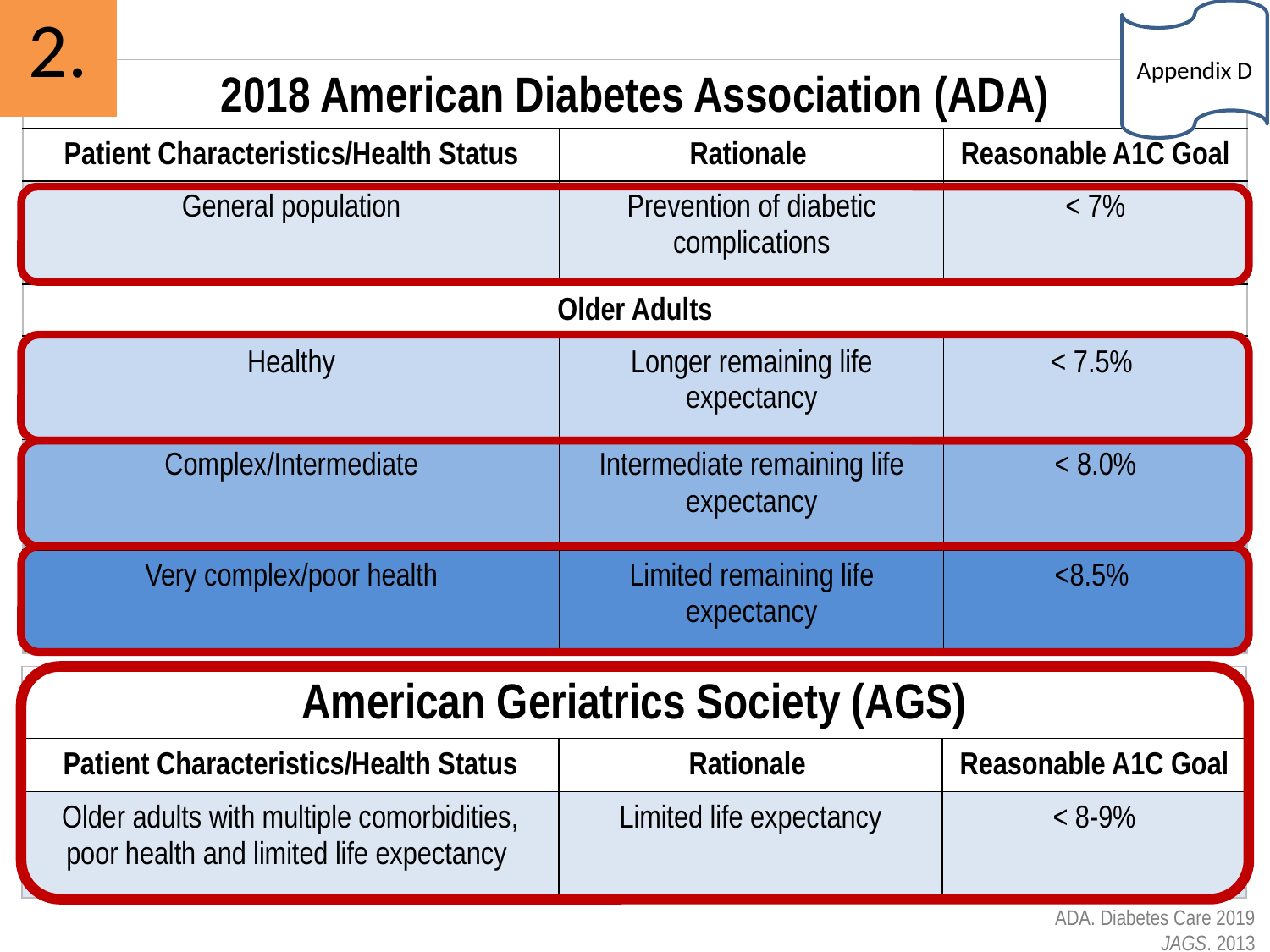

2.
Appendix D
| 2018 American Diabetes Association (ADA) | | |
| --- | --- | --- |
| Patient Characteristics/Health Status | Rationale | Reasonable A1C Goal |
| General population | Prevention of diabetic complications | < 7% |
| Older Adults | | |
| Healthy | Longer remaining life expectancy | < 7.5% |
| Complex/Intermediate | Intermediate remaining life expectancy | < 8.0% |
| Very complex/poor health | Limited remaining life expectancy | <8.5% |
| American Geriatrics Society (AGS) | | |
| --- | --- | --- |
| Patient Characteristics/Health Status | Rationale | Reasonable A1C Goal |
| Older adults with multiple comorbidities, poor health and limited life expectancy | Limited life expectancy | < 8-9% |
ADA. Diabetes Care 2019
JAGS. 2013

## Slide 15
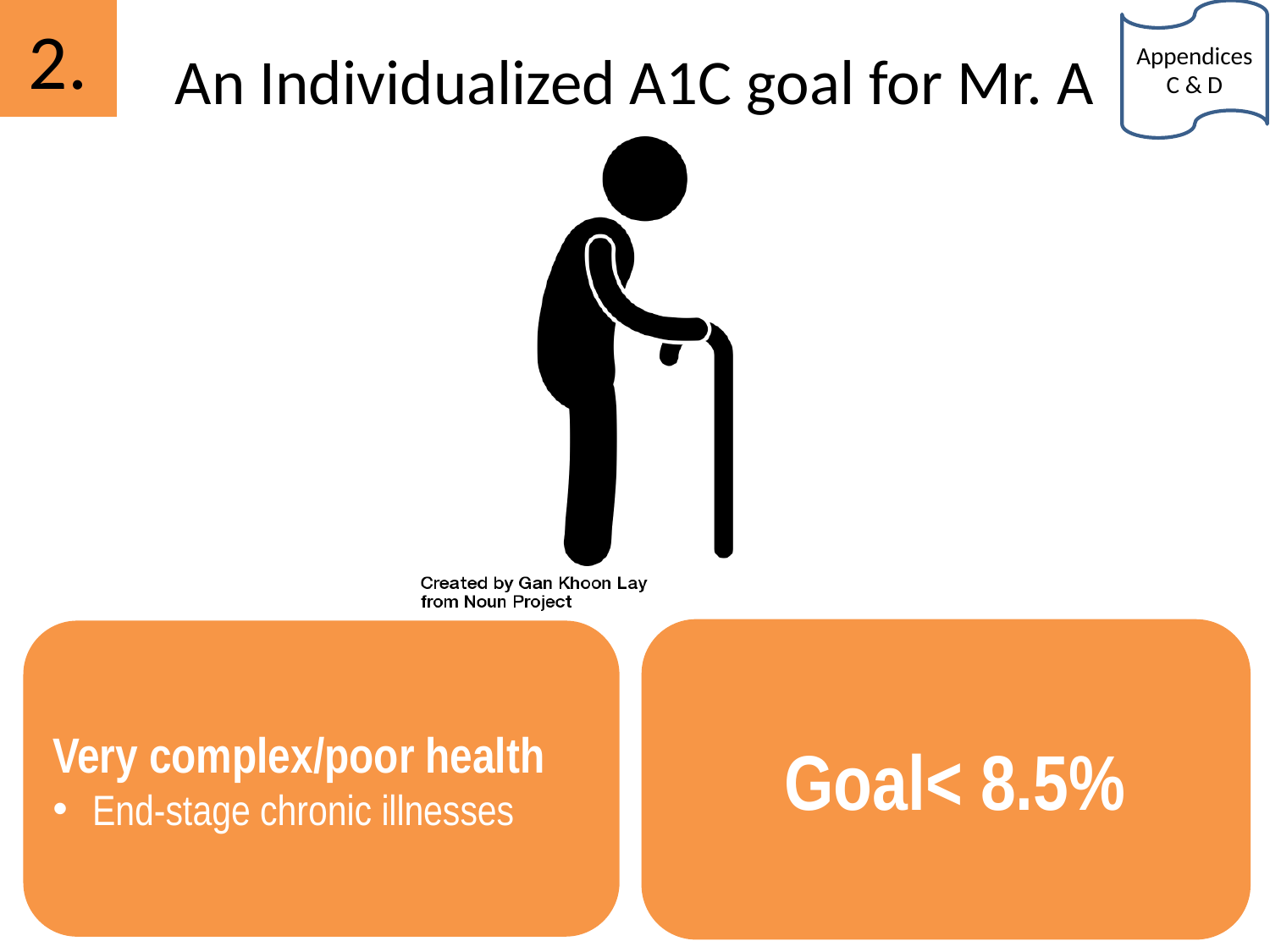

2.
# An Individualized A1C goal for Mr. A
Appendices C & D
Heart failure stage 3
Urinary incontinence
eGFR = 48 mL/min
Type 2 diabetes
 A1C 8.2%
Hypertension
Hyperlipidemia
 Goal< 8.5%
Very complex/poor health
End-stage chronic illnesses

## Slide 16
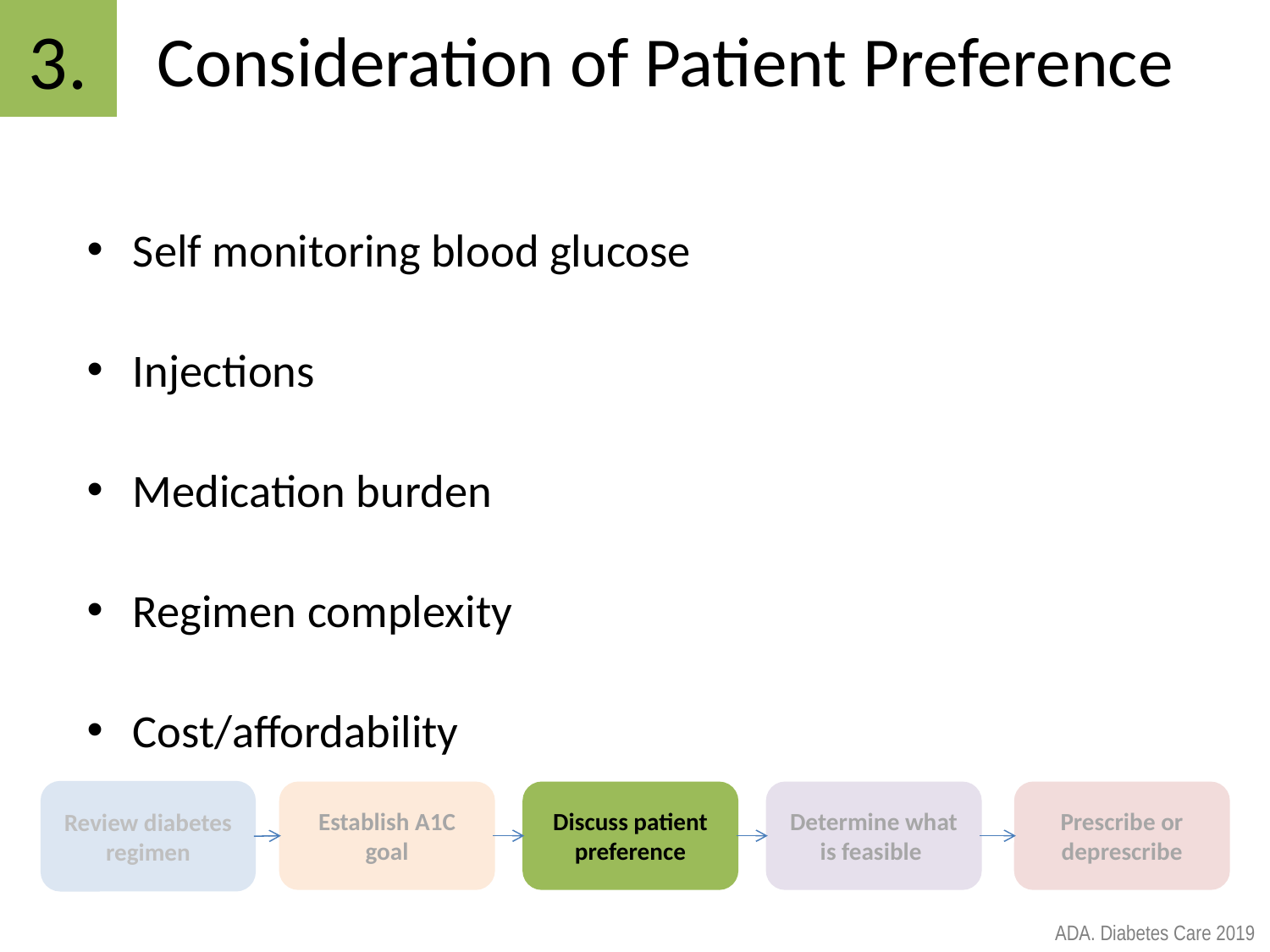

3.
# Consideration of Patient Preference
Self monitoring blood glucose
Injections
Medication burden
Regimen complexity
Cost/affordability
Review diabetes regimen
Establish A1C goal
Discuss patient preference
Determine what is feasible
Prescribe or deprescribe
ADA. Diabetes Care 2019

## Slide 17
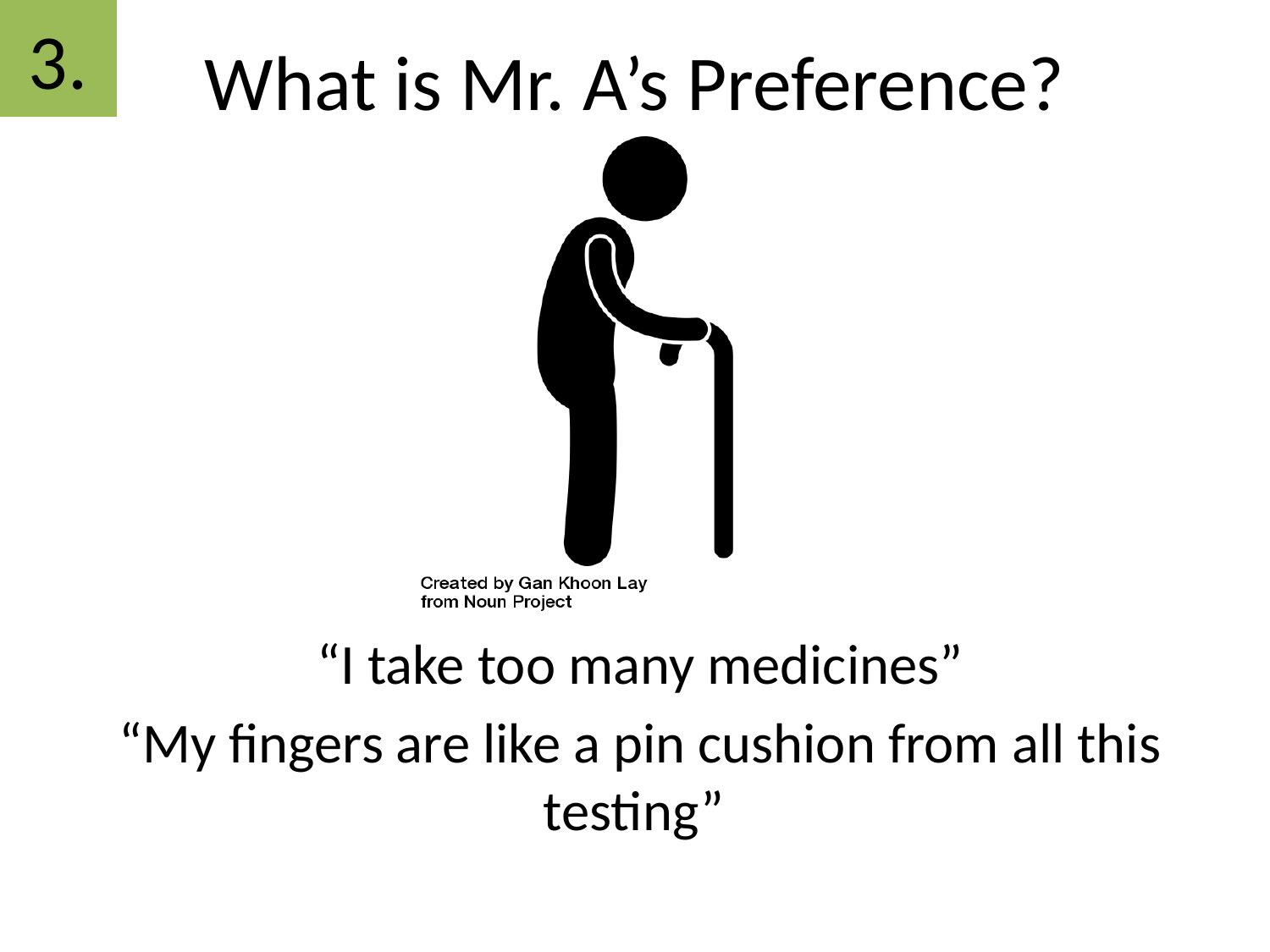

3.
# What is Mr. A’s Preference?
“I take too many medicines”
“My fingers are like a pin cushion from all this testing”

## Slide 18
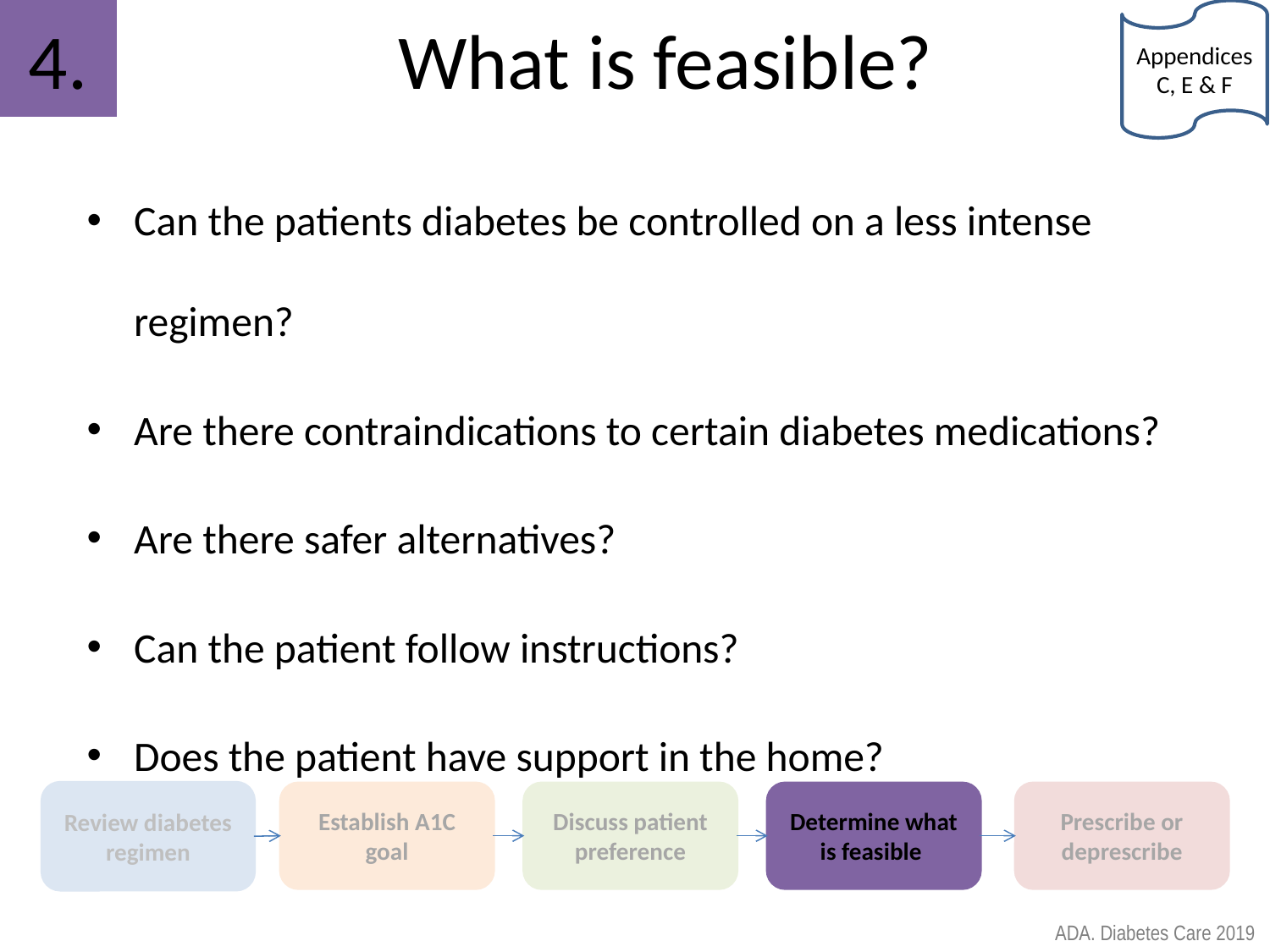

4.
# What is feasible?
Appendices C, E & F
Can the patients diabetes be controlled on a less intense regimen?
Are there contraindications to certain diabetes medications?
Are there safer alternatives?
Can the patient follow instructions?
Does the patient have support in the home?
Review diabetes regimen
Establish A1C goal
Discuss patient preference
Determine what is feasible
Prescribe or deprescribe
ADA. Diabetes Care 2019

## Slide 19
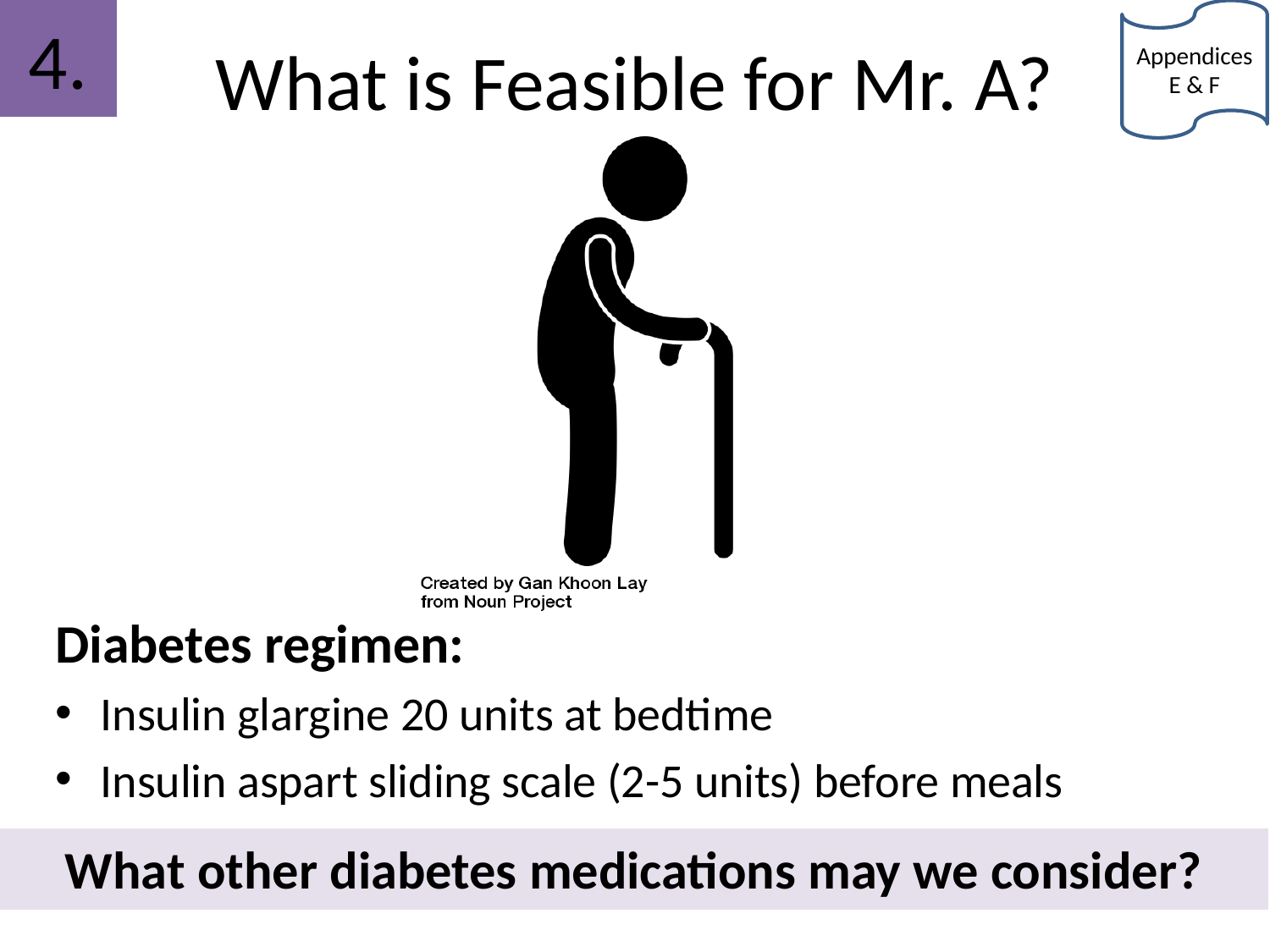

4.
# What is Feasible for Mr. A?
Appendices E & F
Diabetes regimen:
Insulin glargine 20 units at bedtime
Insulin aspart sliding scale (2-5 units) before meals
What other diabetes medications may we consider?

## Slide 20
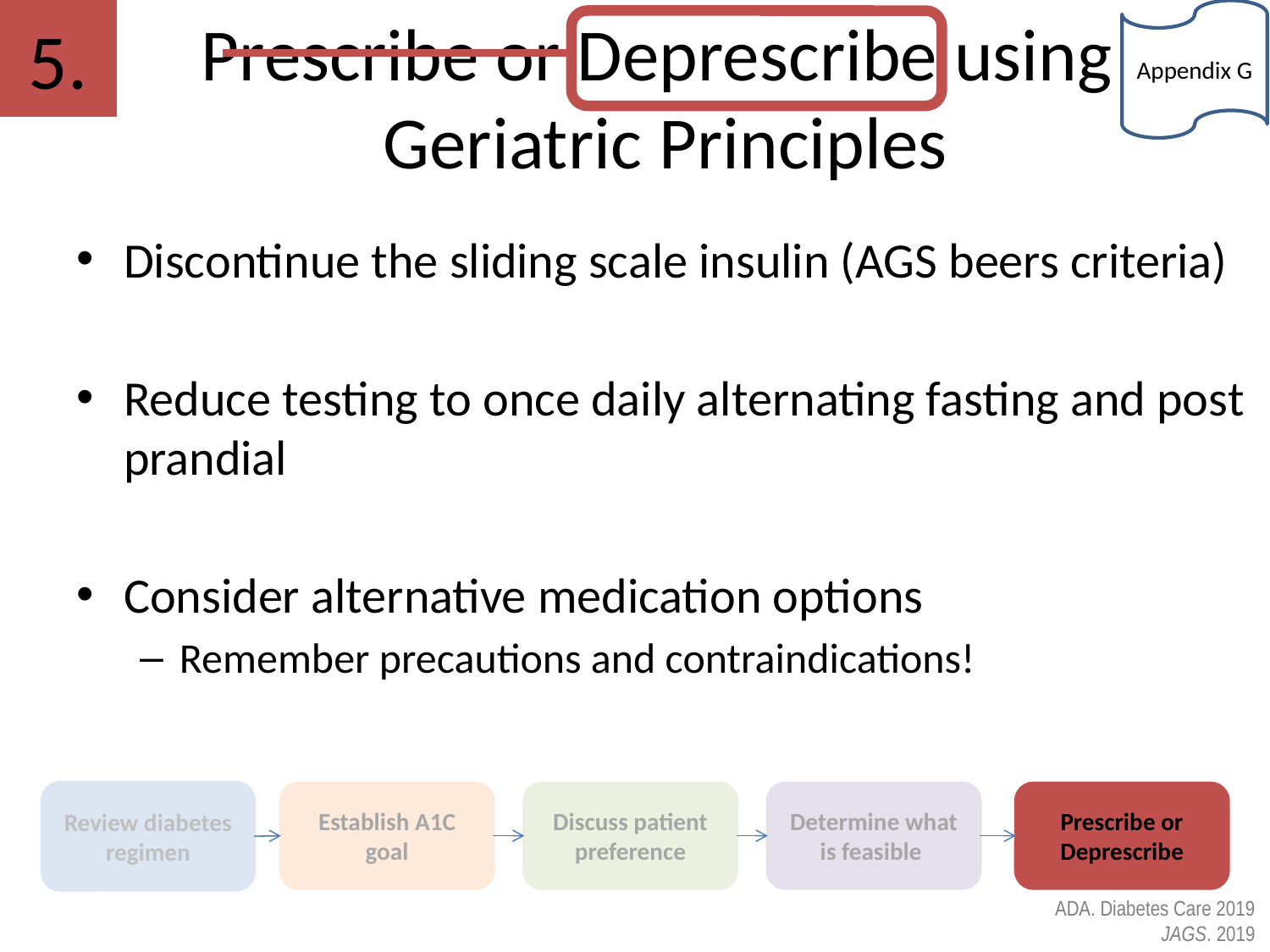

5.
# Prescribe or Deprescribe using Geriatric Principles
Appendix G
Discontinue the sliding scale insulin (AGS beers criteria)
Reduce testing to once daily alternating fasting and post prandial
Consider alternative medication options
Remember precautions and contraindications!
Review diabetes regimen
Establish A1C goal
Discuss patient preference
Determine what is feasible
Prescribe or Deprescribe
ADA. Diabetes Care 2019
JAGS. 2019

## Slide 21
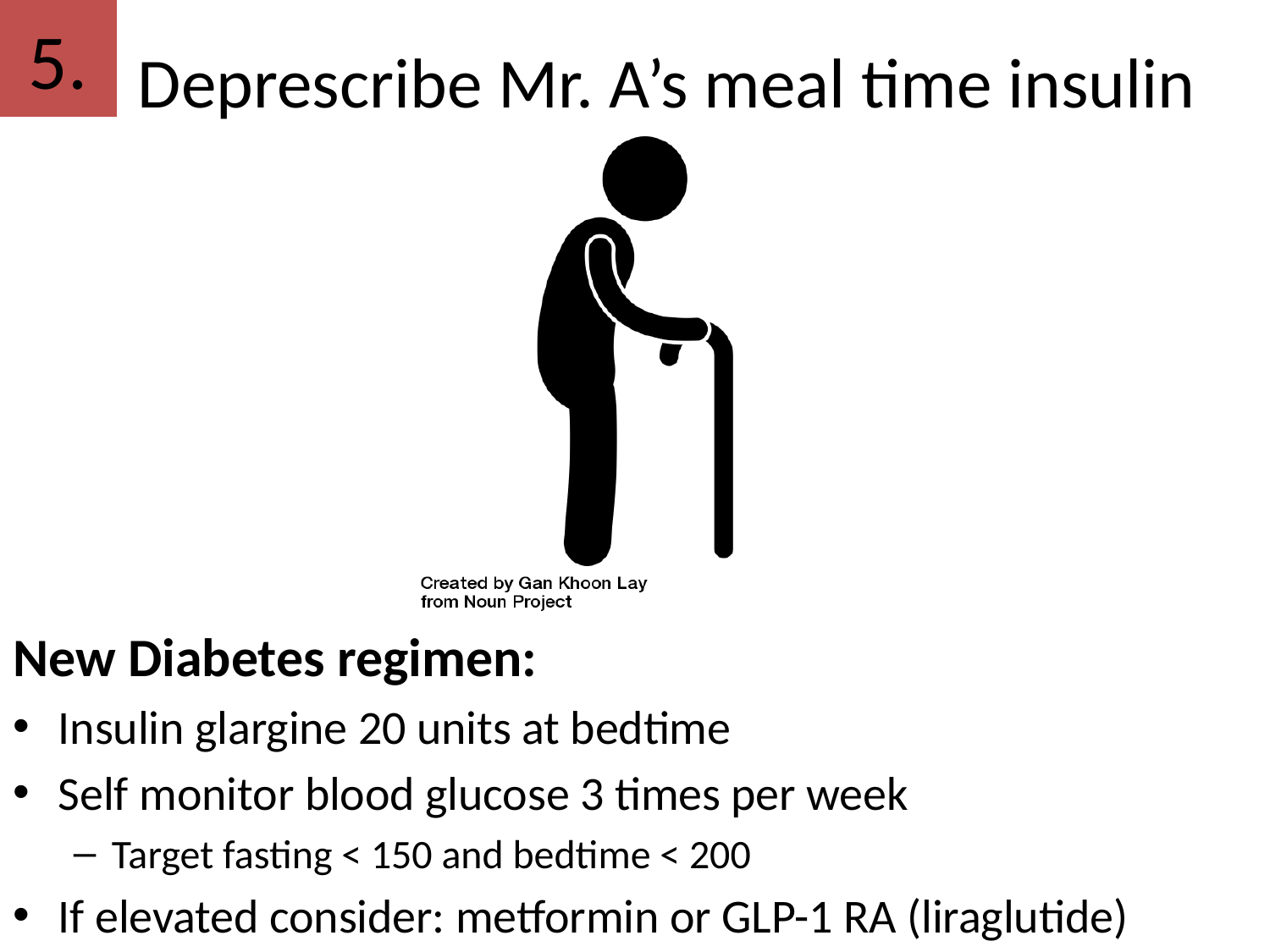

5.
Deprescribe Mr. A’s meal time insulin
New Diabetes regimen:
Insulin glargine 20 units at bedtime
Self monitor blood glucose 3 times per week
Target fasting < 150 and bedtime < 200
If elevated consider: metformin or GLP-1 RA (liraglutide)

## Slide 22
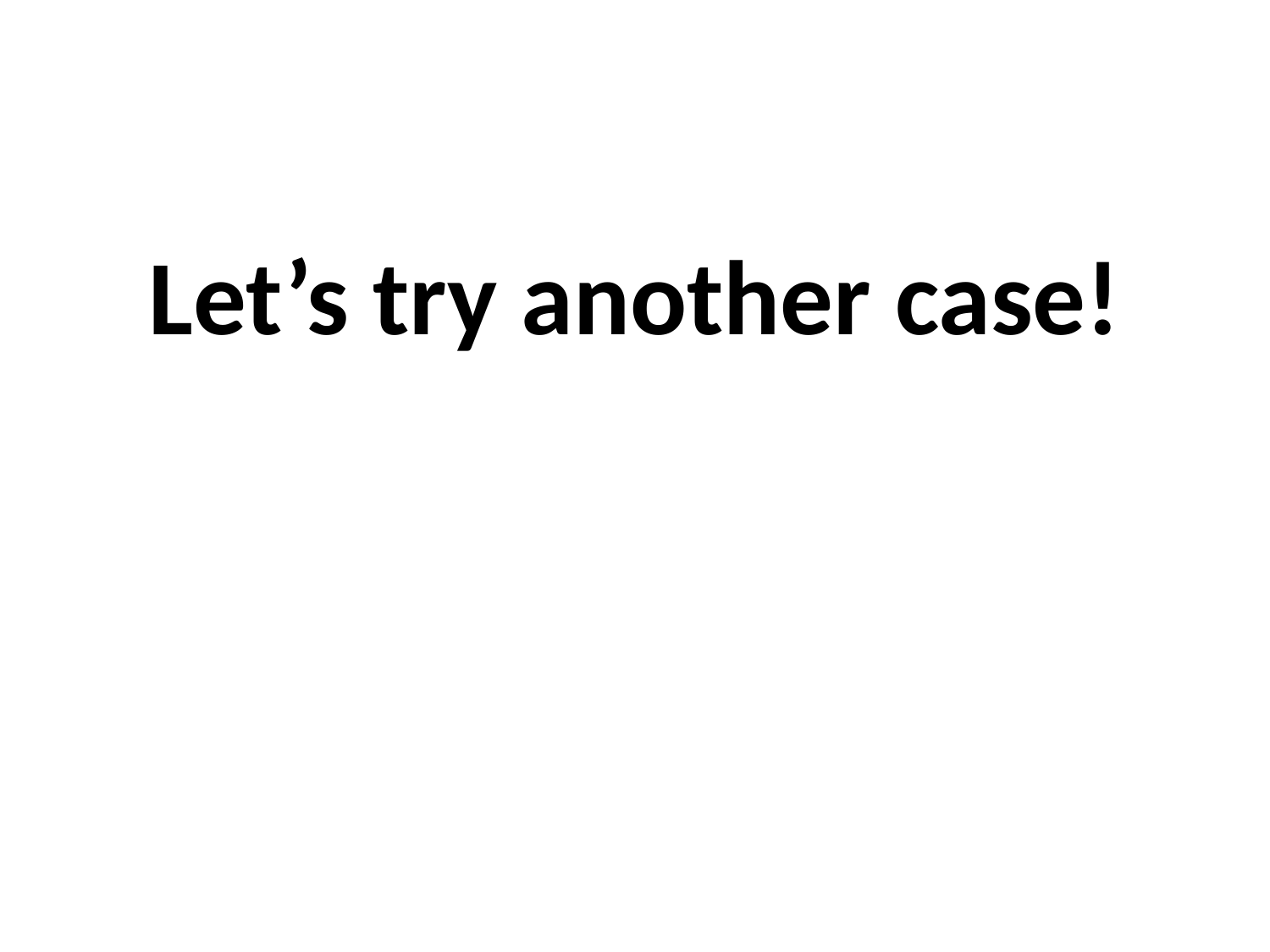

Let’s try another case!

## Slide 23
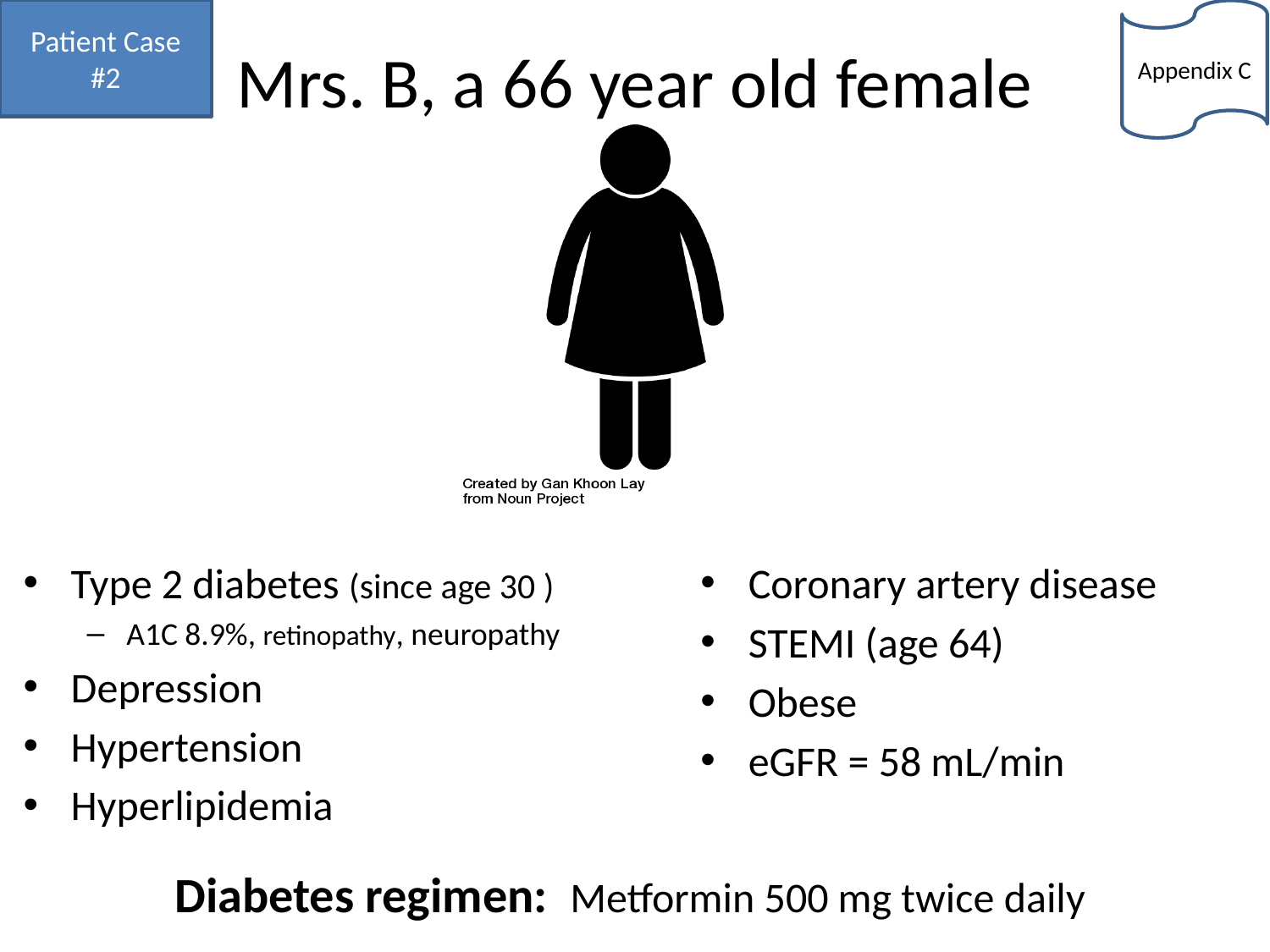

Patient Case #2
# Mrs. B, a 66 year old female
Appendix C
Type 2 diabetes (since age 30 )
A1C 8.9%, retinopathy, neuropathy
Depression
Hypertension
Hyperlipidemia
Coronary artery disease
STEMI (age 64)
Obese
eGFR = 58 mL/min
Diabetes regimen: Metformin 500 mg twice daily

## Slide 24
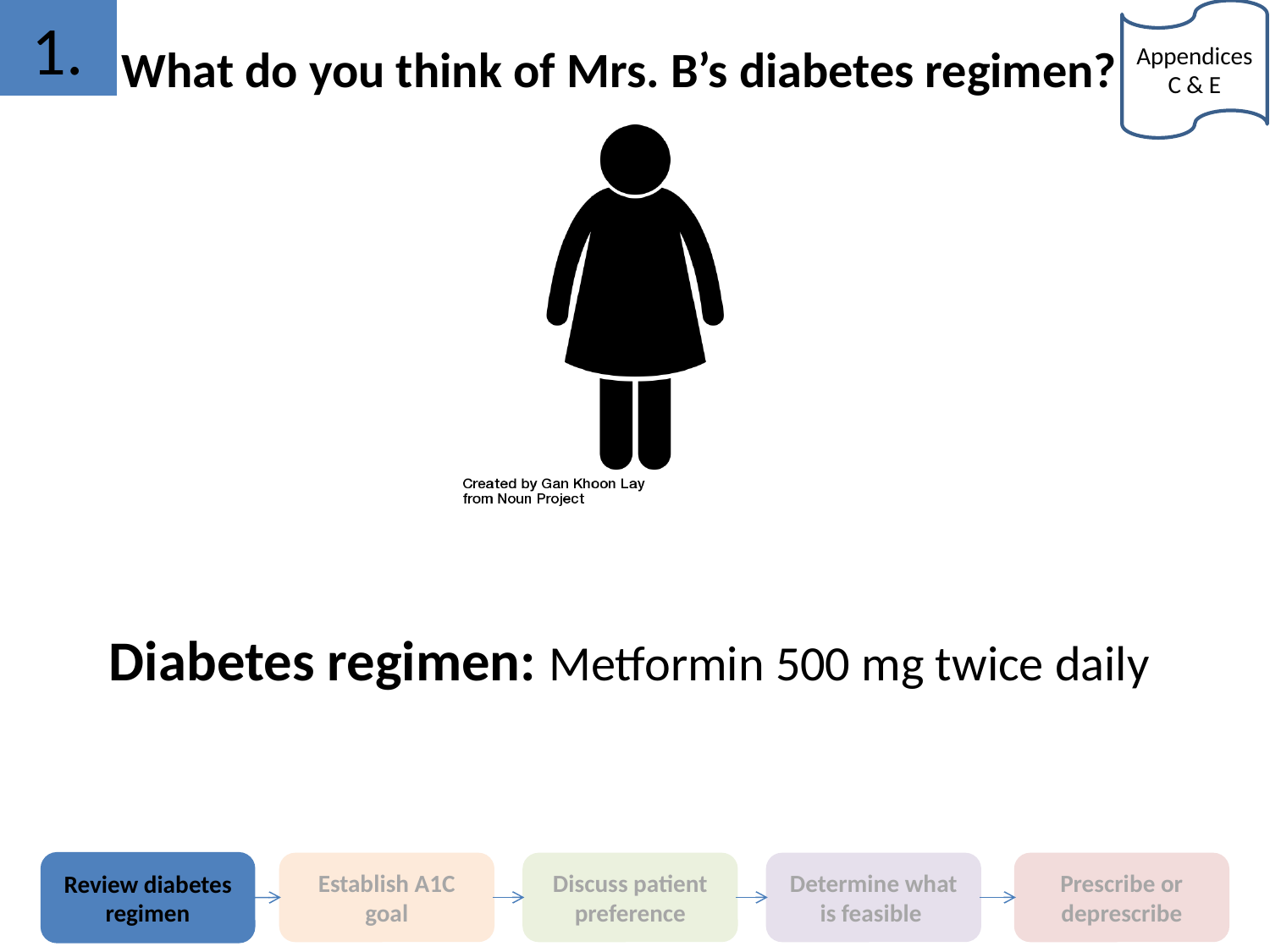

1.
Appendices C & E
What do you think of Mrs. B’s diabetes regimen?
Diabetes regimen: Metformin 500 mg twice daily
Review diabetes regimen
Establish A1C goal
Discuss patient preference
Determine what is feasible
Prescribe or deprescribe

## Slide 25
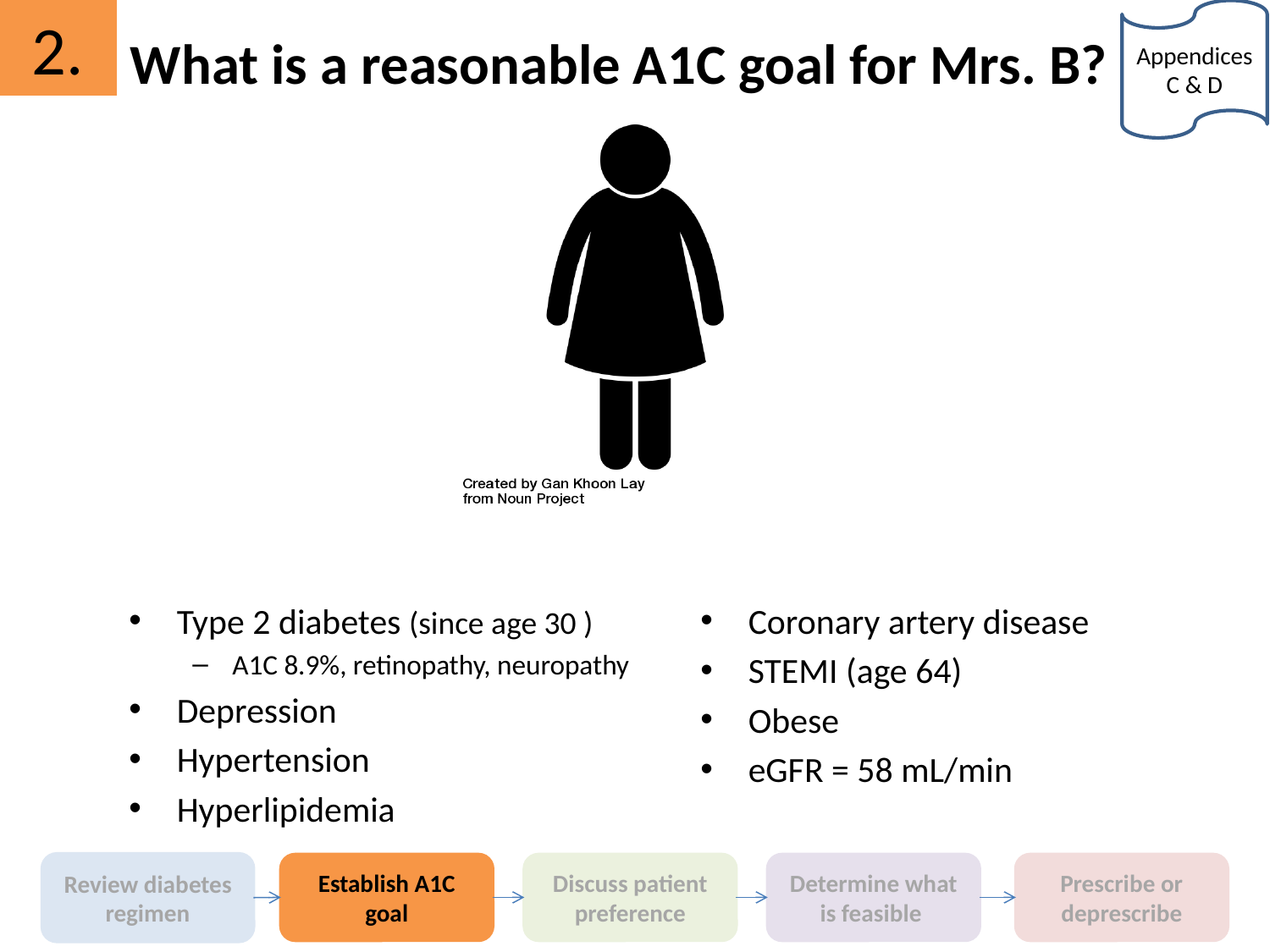

2.
Appendices C & D
What is a reasonable A1C goal for Mrs. B?
Type 2 diabetes (since age 30 )
A1C 8.9%, retinopathy, neuropathy
Depression
Hypertension
Hyperlipidemia
Coronary artery disease
STEMI (age 64)
Obese
eGFR = 58 mL/min
Review diabetes regimen
Establish A1C goal
Discuss patient preference
Determine what is feasible
Prescribe or deprescribe

## Slide 26
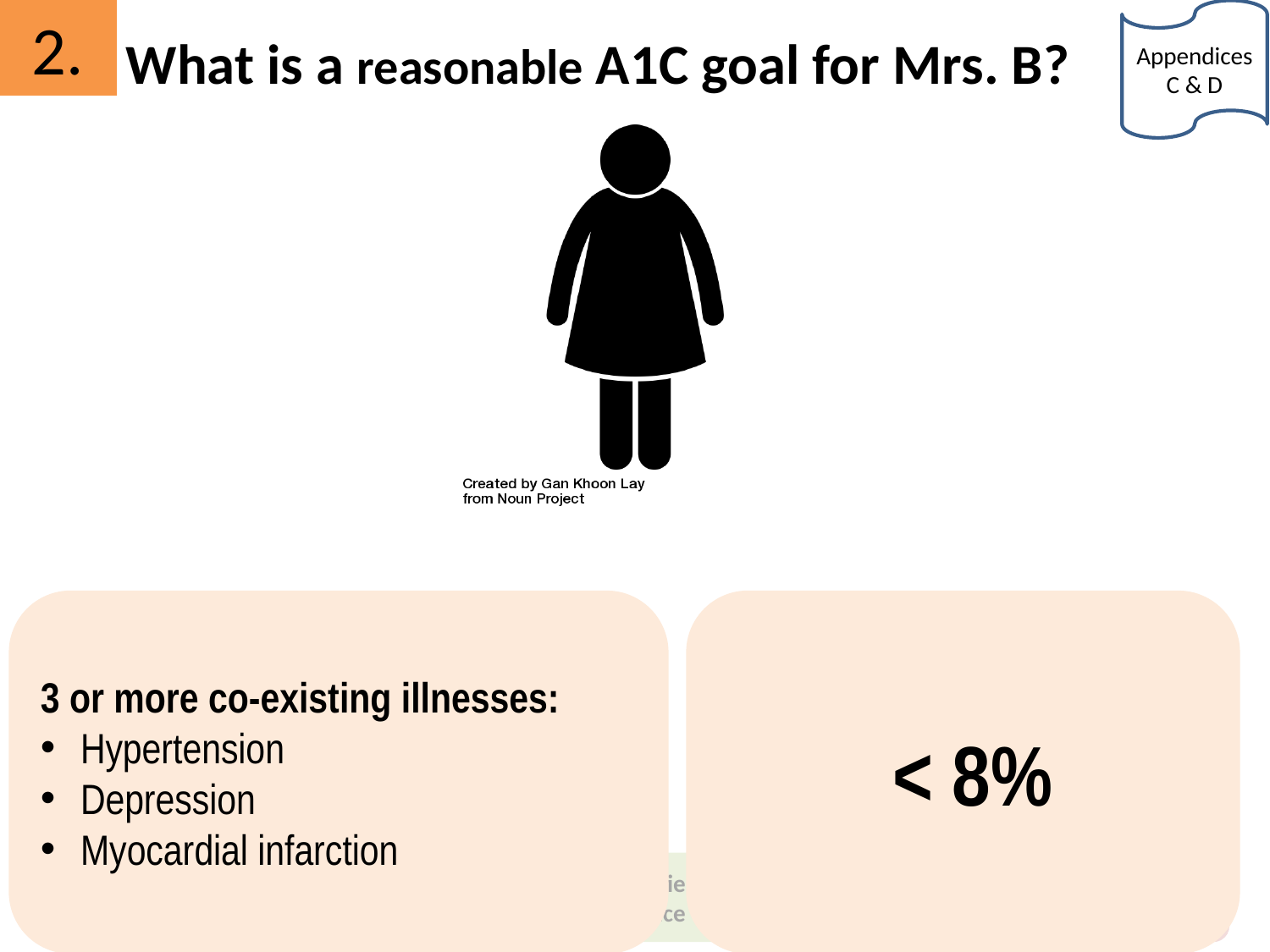

2.
Appendices C & D
What is a reasonable A1C goal for Mrs. B?
3 or more co-existing illnesses:
Hypertension
Depression
Myocardial infarction
Type 2 diabetes (since age 30 )
A1C 8.9%, retinopathy, neuropathy
Depression
Hypertension
Hyperlipidemia
Coronary artery disease
STEMI (age 64)
Obese
eGFR = 58 mL/min
 < 8%
Review diabetes regimen
Establish A1C goal
Discuss patient preference
Determine what is feasible
Prescribe or deprescribe

## Slide 27
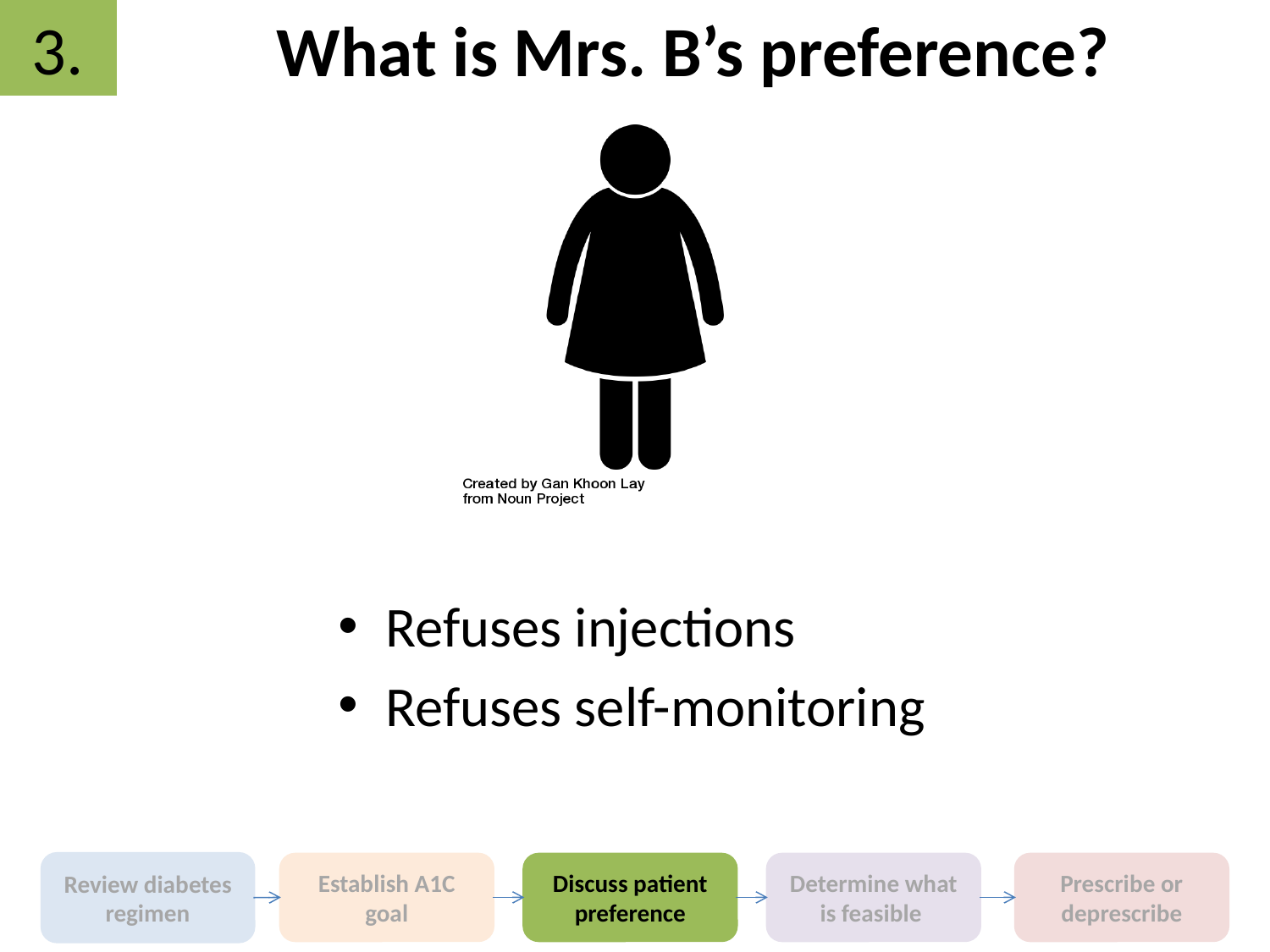

What is Mrs. B’s preference?
3.
Refuses injections
Refuses self-monitoring
Review diabetes regimen
Establish A1C goal
Discuss patient preference
Determine what is feasible
Prescribe or deprescribe

## Slide 28
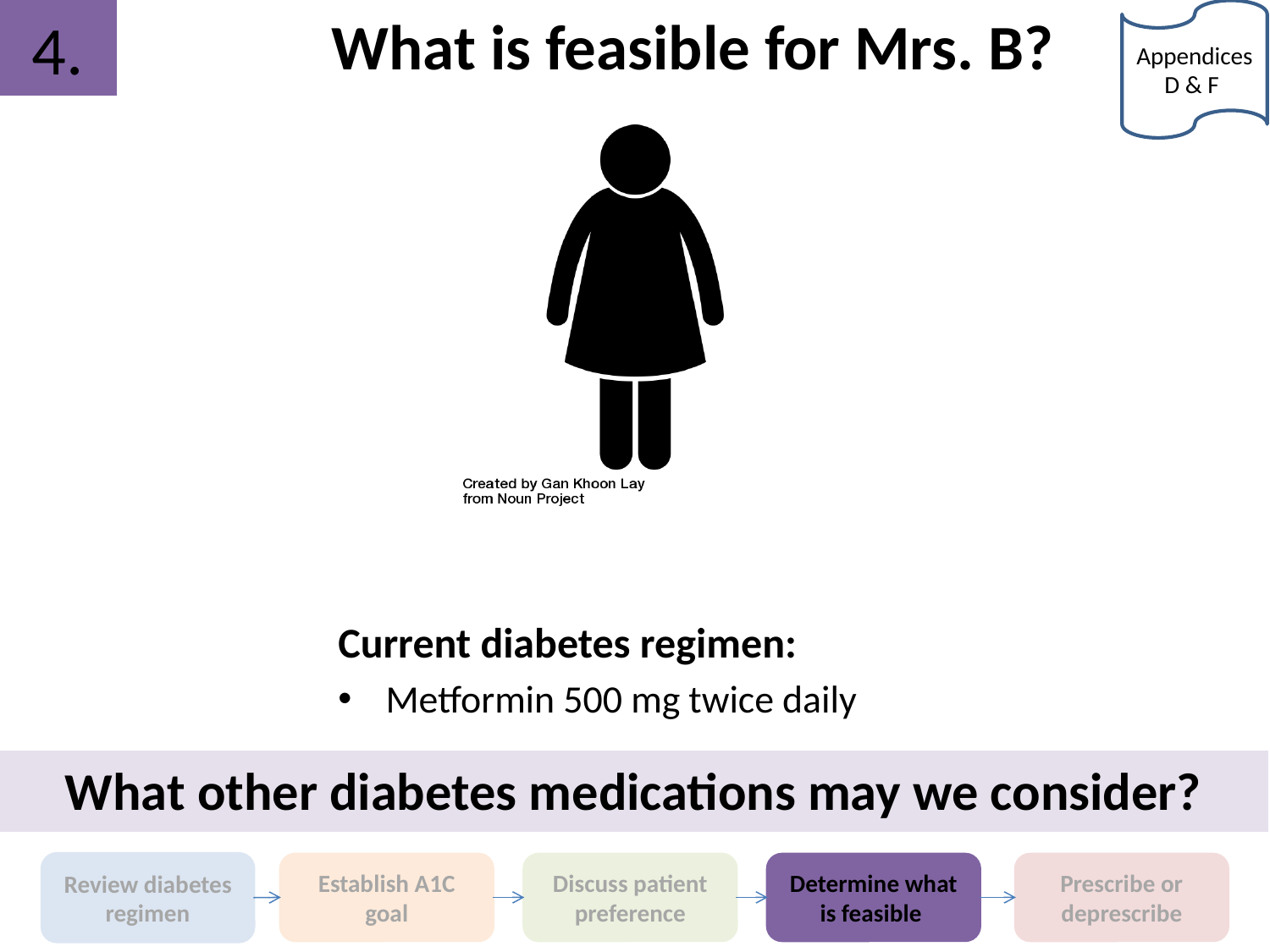

What is feasible for Mrs. B?
4.
Appendices D & F
Current diabetes regimen:
Metformin 500 mg twice daily
What other diabetes medications may we consider?
Review diabetes regimen
Establish A1C goal
Discuss patient preference
Determine what is feasible
Prescribe or deprescribe

## Slide 29
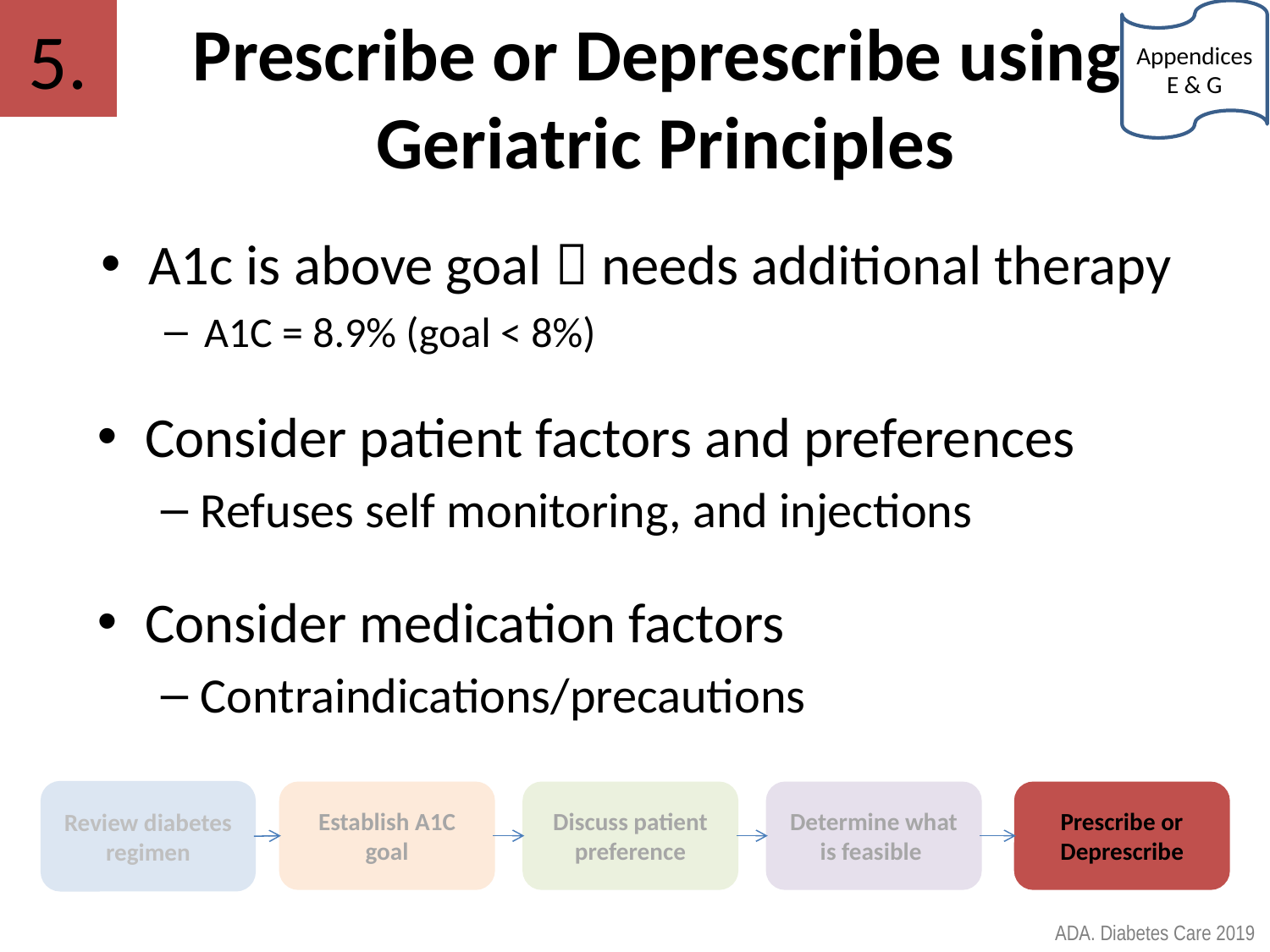

5.
# Prescribe or Deprescribe using Geriatric Principles
Appendices E & G
A1c is above goal  needs additional therapy
A1C = 8.9% (goal < 8%)
Consider patient factors and preferences
Refuses self monitoring, and injections
Consider medication factors
Contraindications/precautions
Review diabetes regimen
Establish A1C goal
Discuss patient preference
Determine what is feasible
Prescribe or Deprescribe
ADA. Diabetes Care 2019

## Slide 30
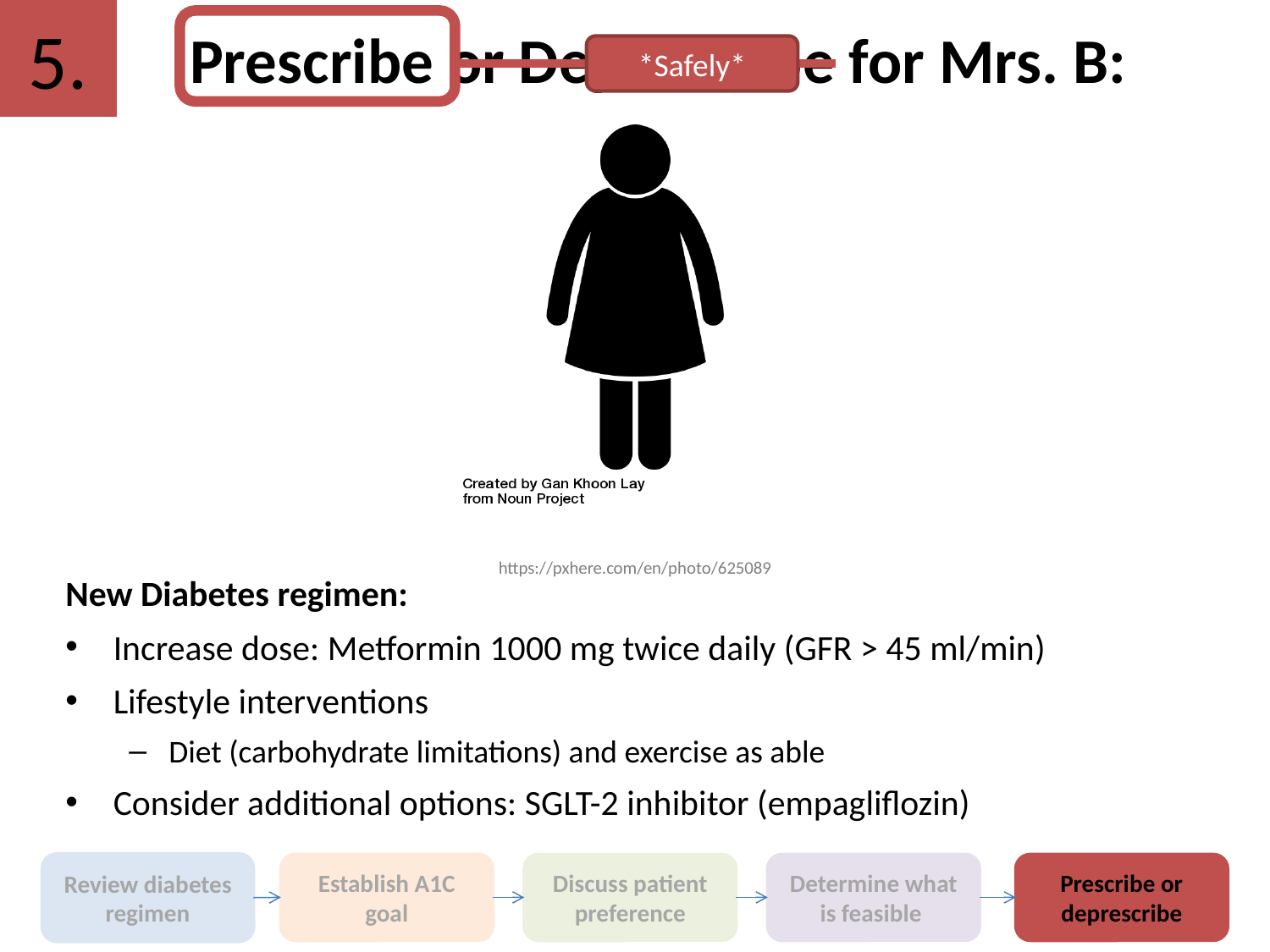

5.
# Prescribe or Deprescribe for Mrs. B:
*Safely*
https://pxhere.com/en/photo/625089
New Diabetes regimen:
Increase dose: Metformin 1000 mg twice daily (GFR > 45 ml/min)
Lifestyle interventions
Diet (carbohydrate limitations) and exercise as able
Consider additional options: SGLT-2 inhibitor (empagliflozin)
Review diabetes regimen
Establish A1C goal
Discuss patient preference
Determine what is feasible
Prescribe or deprescribe

## Slide 31
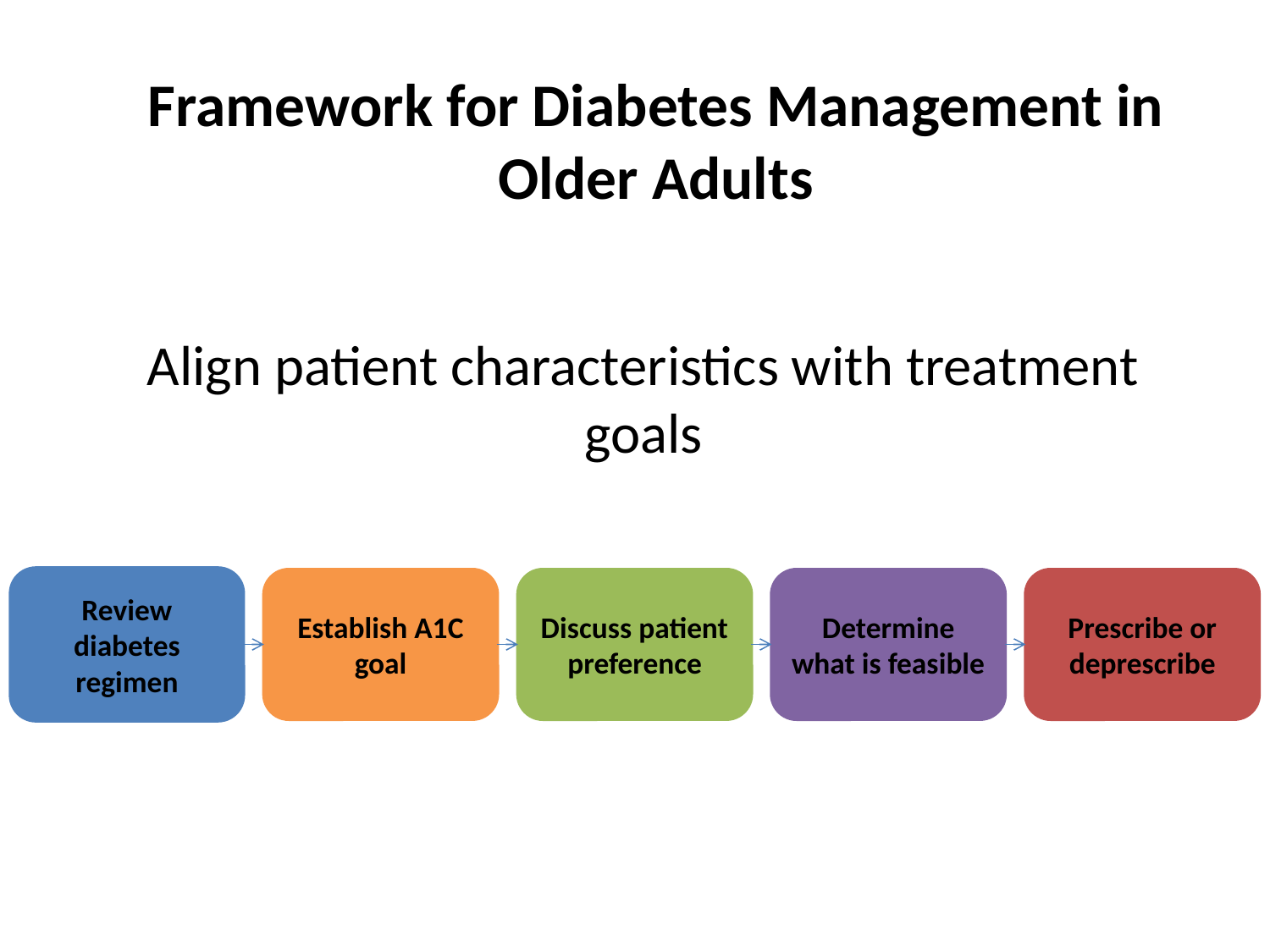

Framework for Diabetes Management in Older Adults
Align patient characteristics with treatment goals
Review diabetes regimen
Establish A1C goal
Discuss patient preference
Determine what is feasible
Prescribe or deprescribe

## Slide 32
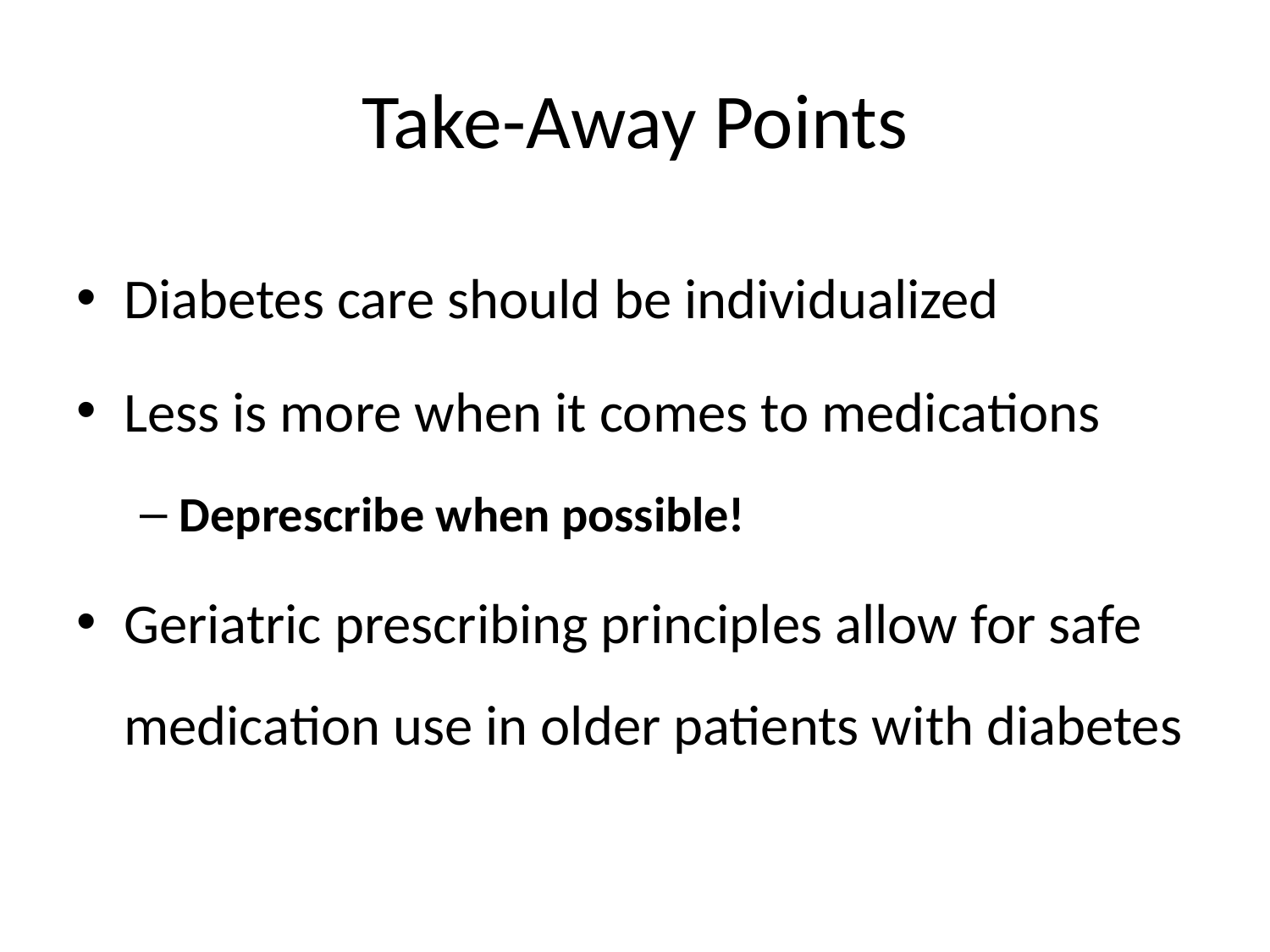

# Take-Away Points
Diabetes care should be individualized
Less is more when it comes to medications
Deprescribe when possible!
Geriatric prescribing principles allow for safe medication use in older patients with diabetes

## Slide 33
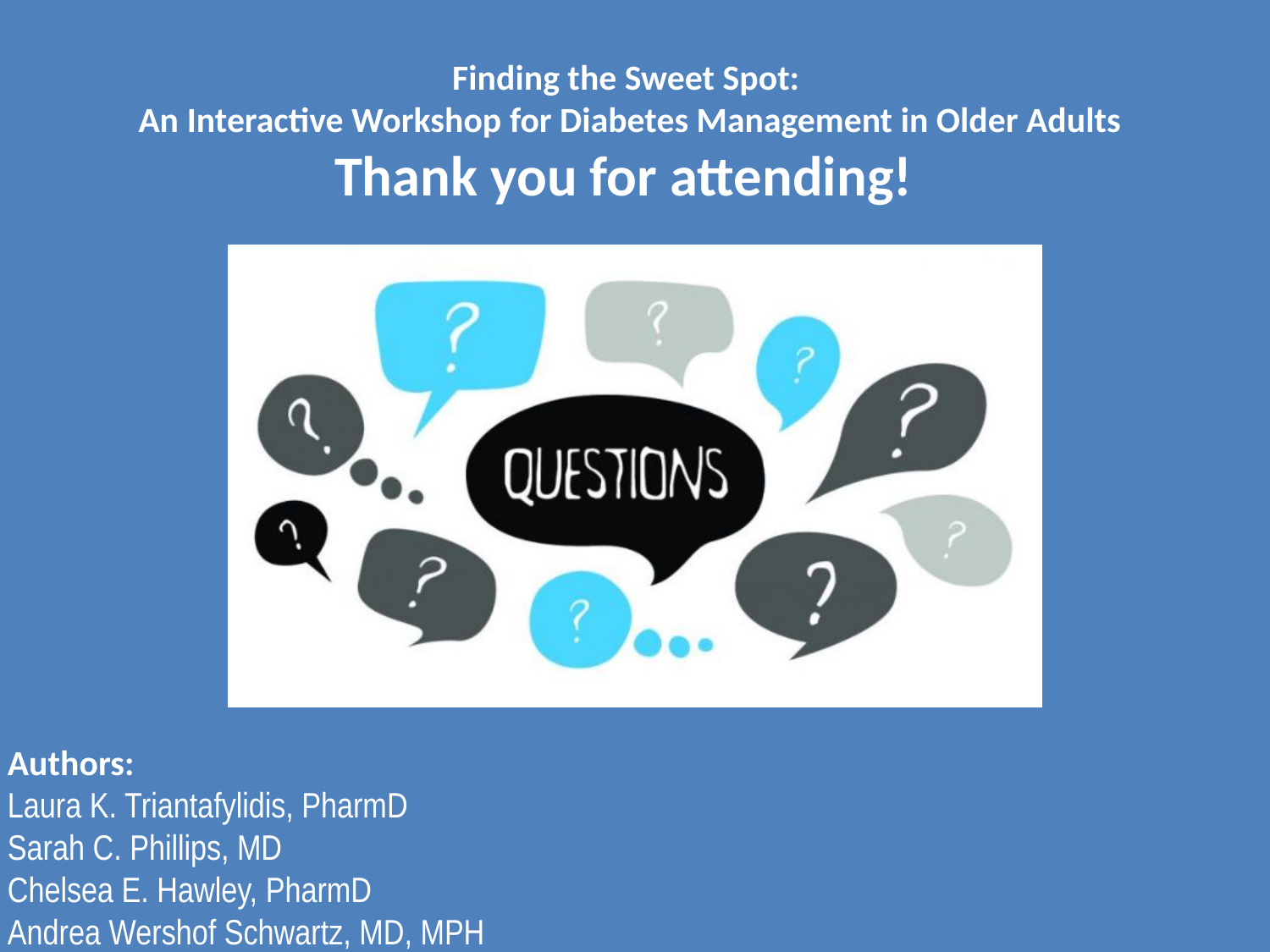

# Finding the Sweet Spot: An Interactive Workshop for Diabetes Management in Older Adults Thank you for attending!
Authors:
Laura K. Triantafylidis, PharmD
Sarah C. Phillips, MD
Chelsea E. Hawley, PharmD
Andrea Wershof Schwartz, MD, MPH

## Slide 34
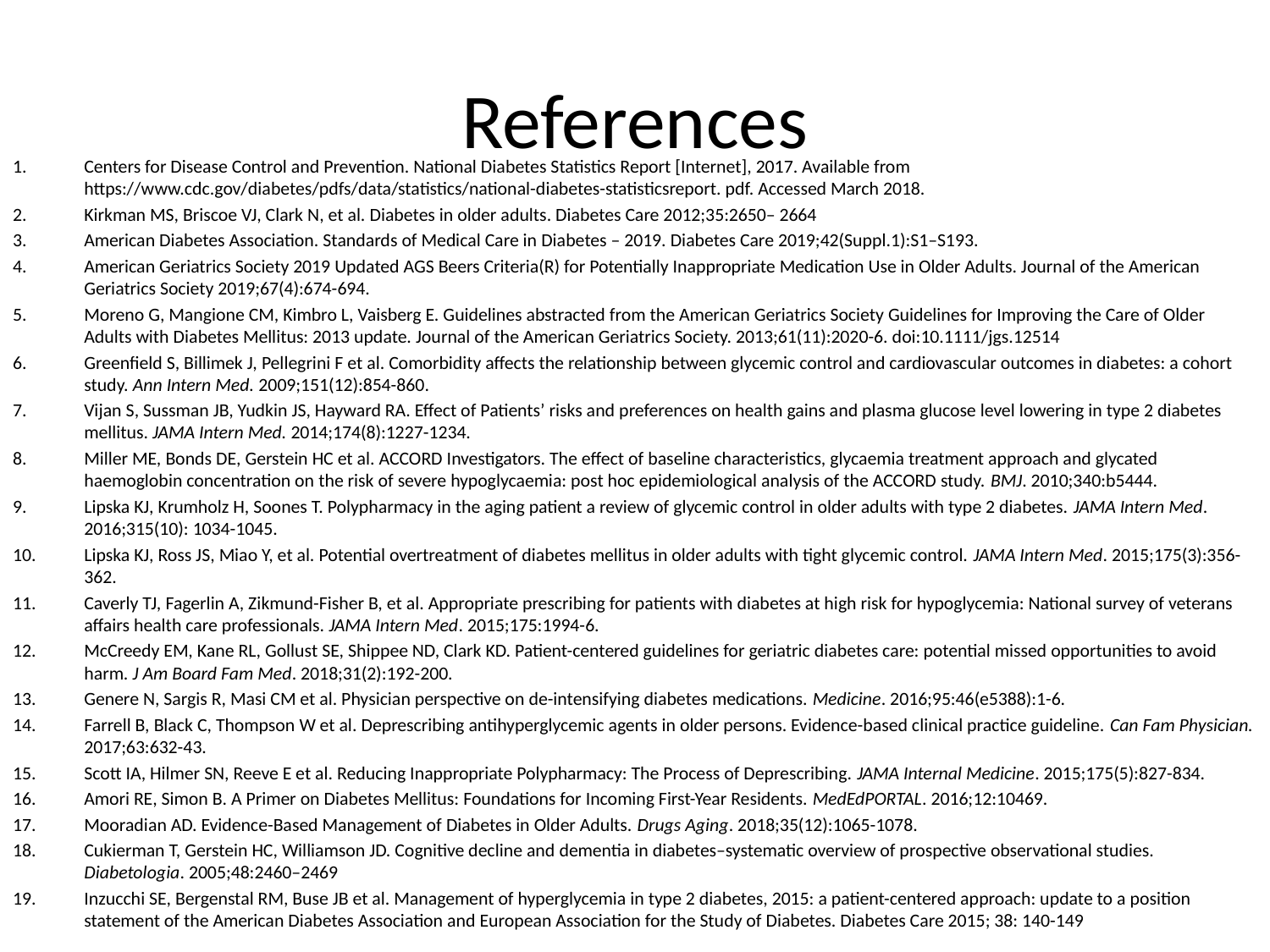

# References
Centers for Disease Control and Prevention. National Diabetes Statistics Report [Internet], 2017. Available from https://www.cdc.gov/diabetes/pdfs/data/statistics/national-diabetes-statisticsreport. pdf. Accessed March 2018.
Kirkman MS, Briscoe VJ, Clark N, et al. Diabetes in older adults. Diabetes Care 2012;35:2650– 2664
American Diabetes Association. Standards of Medical Care in Diabetes – 2019. Diabetes Care 2019;42(Suppl.1):S1–S193.
American Geriatrics Society 2019 Updated AGS Beers Criteria(R) for Potentially Inappropriate Medication Use in Older Adults. Journal of the American Geriatrics Society 2019;67(4):674-694.
Moreno G, Mangione CM, Kimbro L, Vaisberg E. Guidelines abstracted from the American Geriatrics Society Guidelines for Improving the Care of Older Adults with Diabetes Mellitus: 2013 update. Journal of the American Geriatrics Society. 2013;61(11):2020-6. doi:10.1111/jgs.12514
Greenfield S, Billimek J, Pellegrini F et al. Comorbidity affects the relationship between glycemic control and cardiovascular outcomes in diabetes: a cohort study. Ann Intern Med. 2009;151(12):854-860.
Vijan S, Sussman JB, Yudkin JS, Hayward RA. Effect of Patients’ risks and preferences on health gains and plasma glucose level lowering in type 2 diabetes mellitus. JAMA Intern Med. 2014;174(8):1227-1234.
Miller ME, Bonds DE, Gerstein HC et al. ACCORD Investigators. The effect of baseline characteristics, glycaemia treatment approach and glycated haemoglobin concentration on the risk of severe hypoglycaemia: post hoc epidemiological analysis of the ACCORD study. BMJ. 2010;340:b5444.
Lipska KJ, Krumholz H, Soones T. Polypharmacy in the aging patient a review of glycemic control in older adults with type 2 diabetes. JAMA Intern Med. 2016;315(10): 1034-1045.
Lipska KJ, Ross JS, Miao Y, et al. Potential overtreatment of diabetes mellitus in older adults with tight glycemic control. JAMA Intern Med. 2015;175(3):356-362.
Caverly TJ, Fagerlin A, Zikmund-Fisher B, et al. Appropriate prescribing for patients with diabetes at high risk for hypoglycemia: National survey of veterans affairs health care professionals. JAMA Intern Med. 2015;175:1994-6.
McCreedy EM, Kane RL, Gollust SE, Shippee ND, Clark KD. Patient-centered guidelines for geriatric diabetes care: potential missed opportunities to avoid harm. J Am Board Fam Med. 2018;31(2):192-200.
Genere N, Sargis R, Masi CM et al. Physician perspective on de-intensifying diabetes medications. Medicine. 2016;95:46(e5388):1-6.
Farrell B, Black C, Thompson W et al. Deprescribing antihyperglycemic agents in older persons. Evidence-based clinical practice guideline. Can Fam Physician. 2017;63:632-43.
Scott IA, Hilmer SN, Reeve E et al. Reducing Inappropriate Polypharmacy: The Process of Deprescribing. JAMA Internal Medicine. 2015;175(5):827-834.
Amori RE, Simon B. A Primer on Diabetes Mellitus: Foundations for Incoming First-Year Residents. MedEdPORTAL. 2016;12:10469.
Mooradian AD. Evidence-Based Management of Diabetes in Older Adults. Drugs Aging. 2018;35(12):1065-1078.
Cukierman T, Gerstein HC, Williamson JD. Cognitive decline and dementia in diabetes–systematic overview of prospective observational studies. Diabetologia. 2005;48:2460–2469
Inzucchi SE, Bergenstal RM, Buse JB et al. Management of hyperglycemia in type 2 diabetes, 2015: a patient-centered approach: update to a position statement of the American Diabetes Association and European Association for the Study of Diabetes. Diabetes Care 2015; 38: 140-149
