# Supplementary material for: Finding the Sweet Spot: An Interactive Workshop on Diabetes Management in Older Adults
Source: MedEdPORTAL. 2019 Oct 18;15:10845. doi: 10.15766/mep_2374-8265.10845 (PMC6944249; doi:10.15766/mep_2374-8265.10845)
Supplement: Supplementary file 1 — A. Presurvey.docx B. Finding the Sweet Spot Slides.pptx C. Finding the Sweet Spot Activity.docx D. Considerations for A1c Targets.pptx E. Noninsulin Pharmacologic Options.pptx F. Insulin Pharmacologic Options.pptx G. Approach to Prescribing and Deprescribing.pptx H. Postsurvey.docx I. Pre- and Postsurvey Answer Guide.docx [file mep-15-10845-s001.zip › C. Finding the Sweet Spot Activity.docx]

**Determine what is feasible**

**Discuss patient preferences**

**Establish A1C goal**

**Review diabetes regimen**

**Prescribe or deprescribe**

**Case #1**

Mr. A is an 87 year old male

| **Past Medical History** | **Diabetes Regimen** |
| --- | --- |
| - Type 2 diabetes (A1C = 8.2%) - Hypertension - Hyperlipidemia - Heart failure stage 3 - Urinary incontinence - eGFR = 48 mL/min | - Insulin glargine 20 units at bedtime - Insulin aspart sliding scale (2-5 units) before meals |

**Case #2**

Mrs. B is a 66 year old female

| **Past Medical History** | **Diabetes Regimen** |
| --- | --- |
| - Type 2 diabetes (since age 30, A1C = 8.9%)   - Retinopathy, Neuropathy - Hypertension - Hyperlipidemia - Depression - Coronary artery disease - STEMI (age 64) - Obese - eGFR = 58 mL/min | - Metformin 500 mg twice daily |
