# Supplementary material for: Finding the Sweet Spot: An Interactive Workshop on Diabetes Management in Older Adults
Source: MedEdPORTAL. 2019 Oct 18;15:10845. doi: 10.15766/mep_2374-8265.10845 (PMC6944249; doi:10.15766/mep_2374-8265.10845)
Supplement: Supplementary file 1 — A. Presurvey.docx B. Finding the Sweet Spot Slides.pptx C. Finding the Sweet Spot Activity.docx D. Considerations for A1c Targets.pptx E. Noninsulin Pharmacologic Options.pptx F. Insulin Pharmacologic Options.pptx G. Approach to Prescribing and Deprescribing.pptx H. Postsurvey.docx I. Pre- and Postsurvey Answer Guide.docx [file mep-15-10845-s001.zip › D. Considerations for A1c Targets.pptx]

## Slide 1
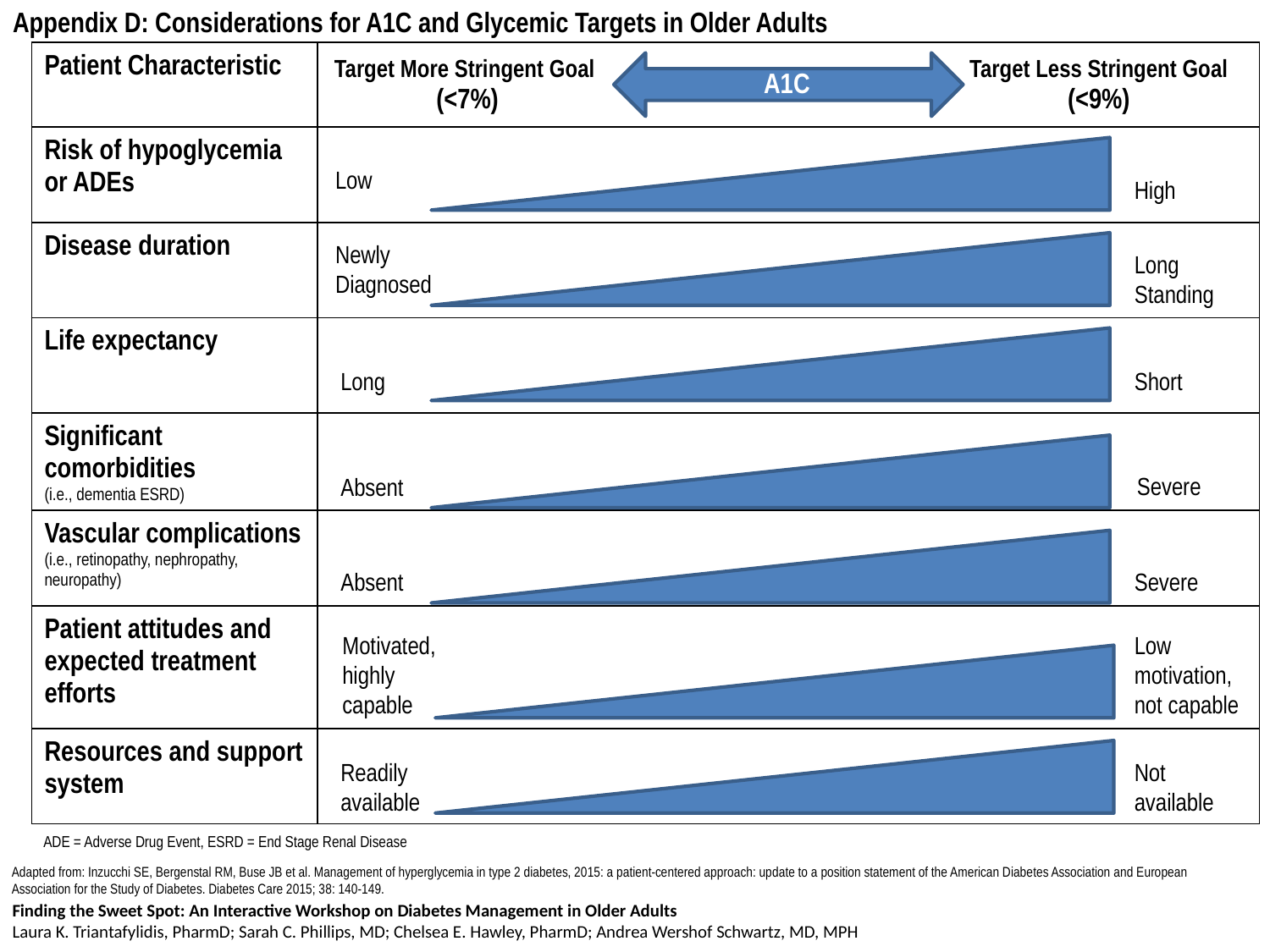

# Appendix D: Considerations for A1C and Glycemic Targets in Older Adults
| Patient Characteristic | Target More Stringent Goal (<7%) | | | Target Less Stringent Goal (<9%) |
| --- | --- | --- | --- | --- |
| Risk of hypoglycemia or ADEs | | | | |
| Disease duration | | | | |
| Life expectancy | | | | |
| Significant comorbidities (i.e., dementia ESRD) | | | | |
| Vascular complications (i.e., retinopathy, nephropathy, neuropathy) | | | | |
| Patient attitudes and expected treatment efforts | | | | |
| Resources and support system | | | | |
A1C
Low
High
Newly
Diagnosed
Long
Standing
Long
Short
Severe
Absent
Absent
Severe
Motivated,
highly
capable
Low motivation, not capable
Readily available
Not
available
ADE = Adverse Drug Event, ESRD = End Stage Renal Disease
Adapted from: Inzucchi SE, Bergenstal RM, Buse JB et al. Management of hyperglycemia in type 2 diabetes, 2015: a patient-centered approach: update to a position statement of the American Diabetes Association and European Association for the Study of Diabetes. Diabetes Care 2015; 38: 140-149.
Finding the Sweet Spot: An Interactive Workshop on Diabetes Management in Older Adults
Laura K. Triantafylidis, PharmD; Sarah C. Phillips, MD; Chelsea E. Hawley, PharmD; Andrea Wershof Schwartz, MD, MPH

## Slide 2
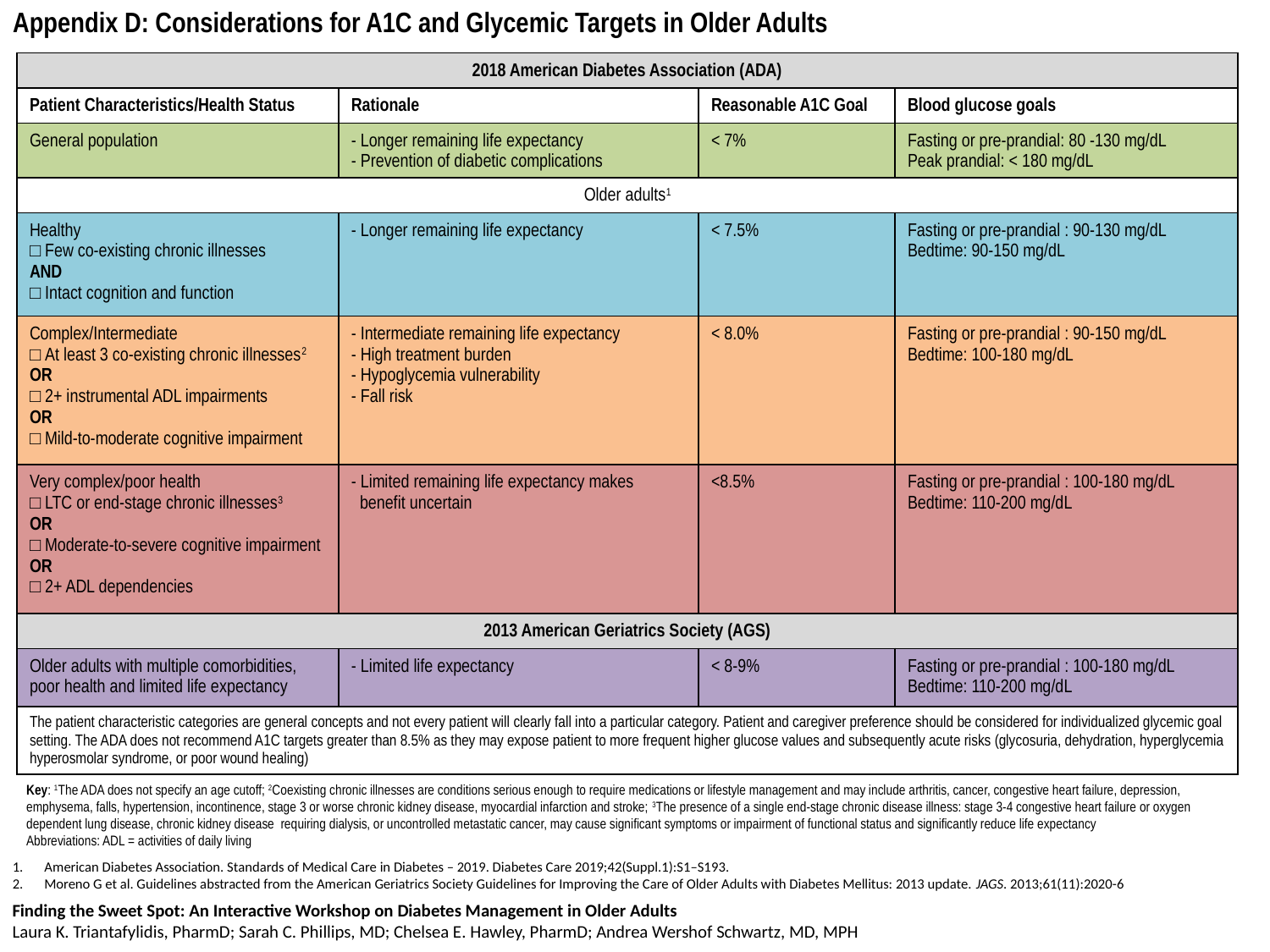

Appendix D: Considerations for A1C and Glycemic Targets in Older Adults
| 2018 American Diabetes Association (ADA) | | | |
| --- | --- | --- | --- |
| Patient Characteristics/Health Status | Rationale | Reasonable A1C Goal | Blood glucose goals |
| General population | - Longer remaining life expectancy - Prevention of diabetic complications | < 7% | Fasting or pre-prandial: 80 -130 mg/dL Peak prandial: < 180 mg/dL |
| Older adults1 | | | |
| Healthy □ Few co-existing chronic illnesses AND □ Intact cognition and function | - Longer remaining life expectancy | < 7.5% | Fasting or pre-prandial : 90-130 mg/dL Bedtime: 90-150 mg/dL |
| Complex/Intermediate □ At least 3 co-existing chronic illnesses2 OR □ 2+ instrumental ADL impairments OR □ Mild-to-moderate cognitive impairment | - Intermediate remaining life expectancy - High treatment burden - Hypoglycemia vulnerability - Fall risk | < 8.0% | Fasting or pre-prandial : 90-150 mg/dL Bedtime: 100-180 mg/dL |
| Very complex/poor health □ LTC or end-stage chronic illnesses3 OR □ Moderate-to-severe cognitive impairment OR □ 2+ ADL dependencies | - Limited remaining life expectancy makes benefit uncertain | <8.5% | Fasting or pre-prandial : 100-180 mg/dL Bedtime: 110-200 mg/dL |
| 2013 American Geriatrics Society (AGS) | | | |
| Older adults with multiple comorbidities, poor health and limited life expectancy | - Limited life expectancy | < 8-9% | Fasting or pre-prandial : 100-180 mg/dL Bedtime: 110-200 mg/dL |
| The patient characteristic categories are general concepts and not every patient will clearly fall into a particular category. Patient and caregiver preference should be considered for individualized glycemic goal setting. The ADA does not recommend A1C targets greater than 8.5% as they may expose patient to more frequent higher glucose values and subsequently acute risks (glycosuria, dehydration, hyperglycemia hyperosmolar syndrome, or poor wound healing) | | | |
Key: 1The ADA does not specify an age cutoff; 2Coexisting chronic illnesses are conditions serious enough to require medications or lifestyle management and may include arthritis, cancer, congestive heart failure, depression, emphysema, falls, hypertension, incontinence, stage 3 or worse chronic kidney disease, myocardial infarction and stroke; 3The presence of a single end-stage chronic disease illness: stage 3-4 congestive heart failure or oxygen dependent lung disease, chronic kidney disease requiring dialysis, or uncontrolled metastatic cancer, may cause significant symptoms or impairment of functional status and significantly reduce life expectancy
Abbreviations: ADL = activities of daily living
American Diabetes Association. Standards of Medical Care in Diabetes – 2019. Diabetes Care 2019;42(Suppl.1):S1–S193.
Moreno G et al. Guidelines abstracted from the American Geriatrics Society Guidelines for Improving the Care of Older Adults with Diabetes Mellitus: 2013 update. JAGS. 2013;61(11):2020-6
Finding the Sweet Spot: An Interactive Workshop on Diabetes Management in Older Adults
Laura K. Triantafylidis, PharmD; Sarah C. Phillips, MD; Chelsea E. Hawley, PharmD; Andrea Wershof Schwartz, MD, MPH
