# Supplementary material for: Finding the Sweet Spot: An Interactive Workshop on Diabetes Management in Older Adults
Source: MedEdPORTAL. 2019 Oct 18;15:10845. doi: 10.15766/mep_2374-8265.10845 (PMC6944249; doi:10.15766/mep_2374-8265.10845)
Supplement: Supplementary file 1 — A. Presurvey.docx B. Finding the Sweet Spot Slides.pptx C. Finding the Sweet Spot Activity.docx D. Considerations for A1c Targets.pptx E. Noninsulin Pharmacologic Options.pptx F. Insulin Pharmacologic Options.pptx G. Approach to Prescribing and Deprescribing.pptx H. Postsurvey.docx I. Pre- and Postsurvey Answer Guide.docx [file mep-15-10845-s001.zip › E. Noninsulin Pharmacologic Options.pptx]

## Slide 1
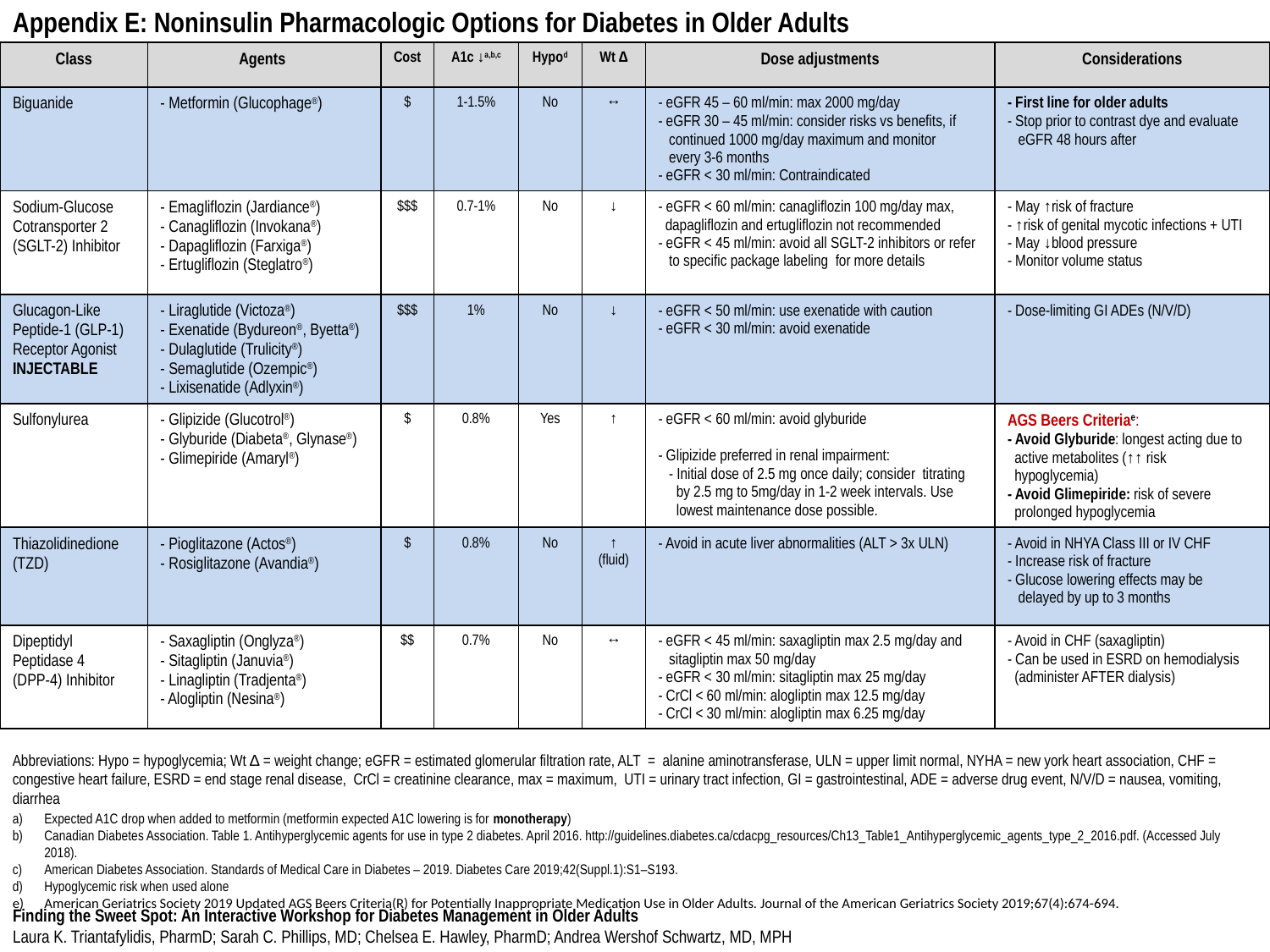

Appendix E: Noninsulin Pharmacologic Options for Diabetes in Older Adults
| Class | Agents | Cost | A1c ↓a,b,c | Hypod | Wt ∆ | Dose adjustments | Considerations |
| --- | --- | --- | --- | --- | --- | --- | --- |
| Biguanide | - Metformin (Glucophage®) | $ | 1-1.5% | No | ↔ | - eGFR 45 – 60 ml/min: max 2000 mg/day - eGFR 30 – 45 ml/min: consider risks vs benefits, if continued 1000 mg/day maximum and monitor every 3-6 months - eGFR < 30 ml/min: Contraindicated | - First line for older adults - Stop prior to contrast dye and evaluate eGFR 48 hours after |
| Sodium-Glucose Cotransporter 2 (SGLT-2) Inhibitor | - Emagliflozin (Jardiance®) - Canagliflozin (Invokana®) - Dapagliflozin (Farxiga®) - Ertugliflozin (Steglatro®) | $$$ | 0.7-1% | No | ↓ | - eGFR < 60 ml/min: canagliflozin 100 mg/day max, dapagliflozin and ertugliflozin not recommended - eGFR < 45 ml/min: avoid all SGLT-2 inhibitors or refer to specific package labeling for more details | - May ↑risk of fracture - ↑risk of genital mycotic infections + UTI - May ↓blood pressure - Monitor volume status |
| Glucagon-Like Peptide-1 (GLP-1) Receptor Agonist INJECTABLE | - Liraglutide (Victoza®) - Exenatide (Bydureon®, Byetta®) - Dulaglutide (Trulicity®) - Semaglutide (Ozempic®) - Lixisenatide (Adlyxin®) | $$$ | 1% | No | ↓ | - eGFR < 50 ml/min: use exenatide with caution - eGFR < 30 ml/min: avoid exenatide | - Dose-limiting GI ADEs (N/V/D) |
| Sulfonylurea | - Glipizide (Glucotrol®) - Glyburide (Diabeta®, Glynase®) - Glimepiride (Amaryl®) | $ | 0.8% | Yes | ↑ | - eGFR < 60 ml/min: avoid glyburide - Glipizide preferred in renal impairment: - Initial dose of 2.5 mg once daily; consider titrating by 2.5 mg to 5mg/day in 1-2 week intervals. Use lowest maintenance dose possible. | AGS Beers Criteriae: - Avoid Glyburide: longest acting due to active metabolites (↑↑ risk hypoglycemia) - Avoid Glimepiride: risk of severe prolonged hypoglycemia |
| Thiazolidinedione (TZD) | - Pioglitazone (Actos®) - Rosiglitazone (Avandia®) | $ | 0.8% | No | ↑ (fluid) | - Avoid in acute liver abnormalities (ALT > 3x ULN) | - Avoid in NHYA Class III or IV CHF - Increase risk of fracture - Glucose lowering effects may be delayed by up to 3 months |
| Dipeptidyl Peptidase 4 (DPP-4) Inhibitor | - Saxagliptin (Onglyza®) - Sitagliptin (Januvia®) - Linagliptin (Tradjenta®) - Alogliptin (Nesina®) | $$ | 0.7% | No | ↔ | - eGFR < 45 ml/min: saxagliptin max 2.5 mg/day and sitagliptin max 50 mg/day - eGFR < 30 ml/min: sitagliptin max 25 mg/day - CrCl < 60 ml/min: alogliptin max 12.5 mg/day - CrCl < 30 ml/min: alogliptin max 6.25 mg/day | - Avoid in CHF (saxagliptin) - Can be used in ESRD on hemodialysis (administer AFTER dialysis) |
Abbreviations: Hypo = hypoglycemia; Wt ∆ = weight change; eGFR = estimated glomerular filtration rate, ALT = alanine aminotransferase, ULN = upper limit normal, NYHA = new york heart association, CHF = congestive heart failure, ESRD = end stage renal disease, CrCl = creatinine clearance, max = maximum, UTI = urinary tract infection, GI = gastrointestinal, ADE = adverse drug event, N/V/D = nausea, vomiting, diarrhea
Expected A1C drop when added to metformin (metformin expected A1C lowering is for monotherapy)
Canadian Diabetes Association. Table 1. Antihyperglycemic agents for use in type 2 diabetes. April 2016. http://guidelines.diabetes.ca/cdacpg_resources/Ch13_Table1_Antihyperglycemic_agents_type_2_2016.pdf. (Accessed July 2018).
American Diabetes Association. Standards of Medical Care in Diabetes – 2019. Diabetes Care 2019;42(Suppl.1):S1–S193.
Hypoglycemic risk when used alone
American Geriatrics Society 2019 Updated AGS Beers Criteria(R) for Potentially Inappropriate Medication Use in Older Adults. Journal of the American Geriatrics Society 2019;67(4):674-694.
Finding the Sweet Spot: An Interactive Workshop for Diabetes Management in Older Adults
Laura K. Triantafylidis, PharmD; Sarah C. Phillips, MD; Chelsea E. Hawley, PharmD; Andrea Wershof Schwartz, MD, MPH

## Slide 2
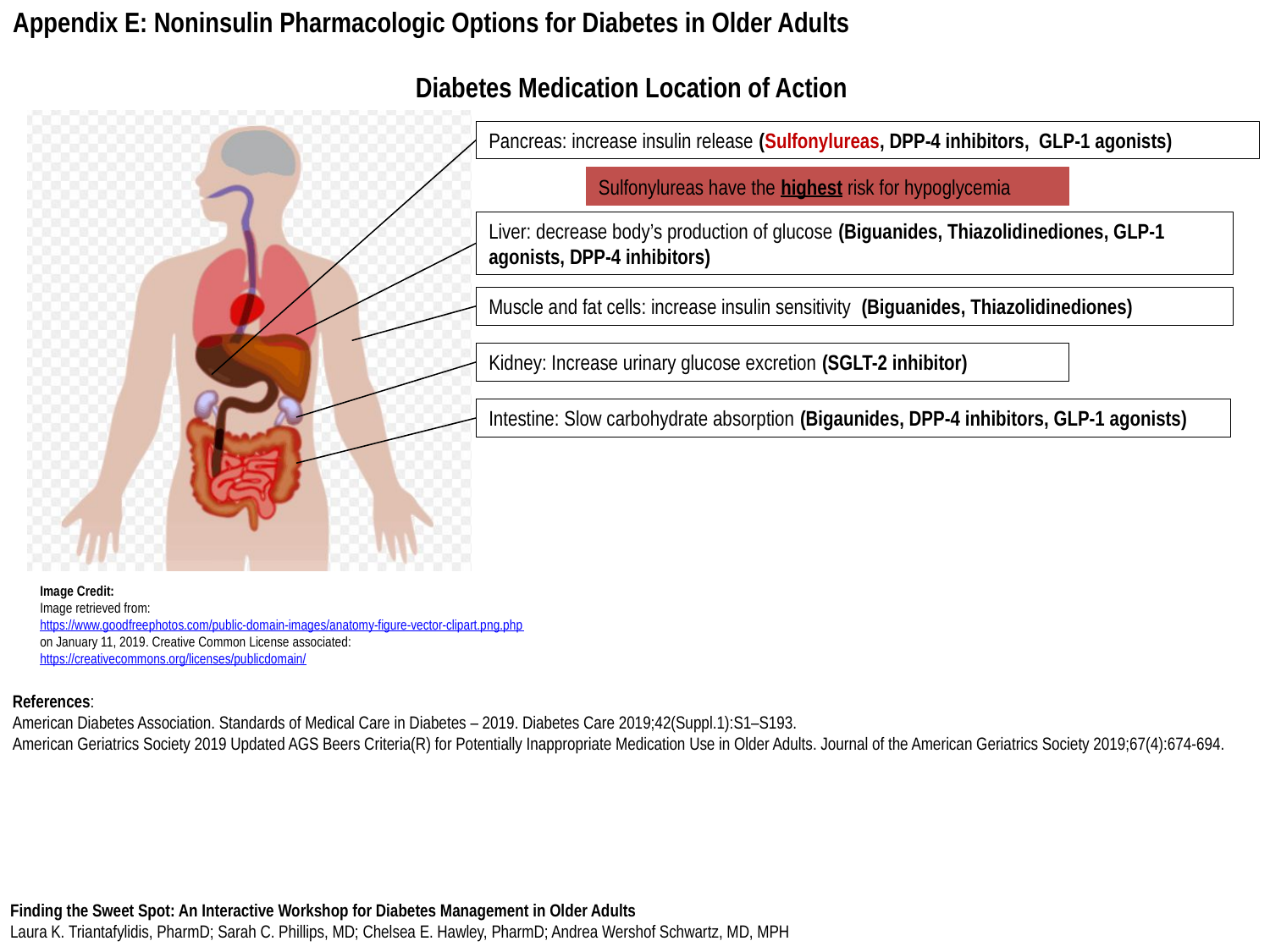

Appendix E: Noninsulin Pharmacologic Options for Diabetes in Older Adults
Diabetes Medication Location of Action
Pancreas: increase insulin release (Sulfonylureas, DPP-4 inhibitors, GLP-1 agonists)
Sulfonylureas have the highest risk for hypoglycemia
Liver: decrease body’s production of glucose (Biguanides, Thiazolidinediones, GLP-1 agonists, DPP-4 inhibitors)
Muscle and fat cells: increase insulin sensitivity (Biguanides, Thiazolidinediones)
Kidney: Increase urinary glucose excretion (SGLT-2 inhibitor)
Intestine: Slow carbohydrate absorption (Bigaunides, DPP-4 inhibitors, GLP-1 agonists)
Image Credit:
Image retrieved from: https://www.goodfreephotos.com/public-domain-images/anatomy-figure-vector-clipart.png.php on January 11, 2019. Creative Common License associated: https://creativecommons.org/licenses/publicdomain/
References:
American Diabetes Association. Standards of Medical Care in Diabetes – 2019. Diabetes Care 2019;42(Suppl.1):S1–S193.
American Geriatrics Society 2019 Updated AGS Beers Criteria(R) for Potentially Inappropriate Medication Use in Older Adults. Journal of the American Geriatrics Society 2019;67(4):674-694.
Finding the Sweet Spot: An Interactive Workshop for Diabetes Management in Older Adults
Laura K. Triantafylidis, PharmD; Sarah C. Phillips, MD; Chelsea E. Hawley, PharmD; Andrea Wershof Schwartz, MD, MPH
