# Supplementary material for: Finding the Sweet Spot: An Interactive Workshop on Diabetes Management in Older Adults
Source: MedEdPORTAL. 2019 Oct 18;15:10845. doi: 10.15766/mep_2374-8265.10845 (PMC6944249; doi:10.15766/mep_2374-8265.10845)
Supplement: Supplementary file 1 — A. Presurvey.docx B. Finding the Sweet Spot Slides.pptx C. Finding the Sweet Spot Activity.docx D. Considerations for A1c Targets.pptx E. Noninsulin Pharmacologic Options.pptx F. Insulin Pharmacologic Options.pptx G. Approach to Prescribing and Deprescribing.pptx H. Postsurvey.docx I. Pre- and Postsurvey Answer Guide.docx [file mep-15-10845-s001.zip › F. Insulin Pharmacologic Options.pptx]

## Slide 1
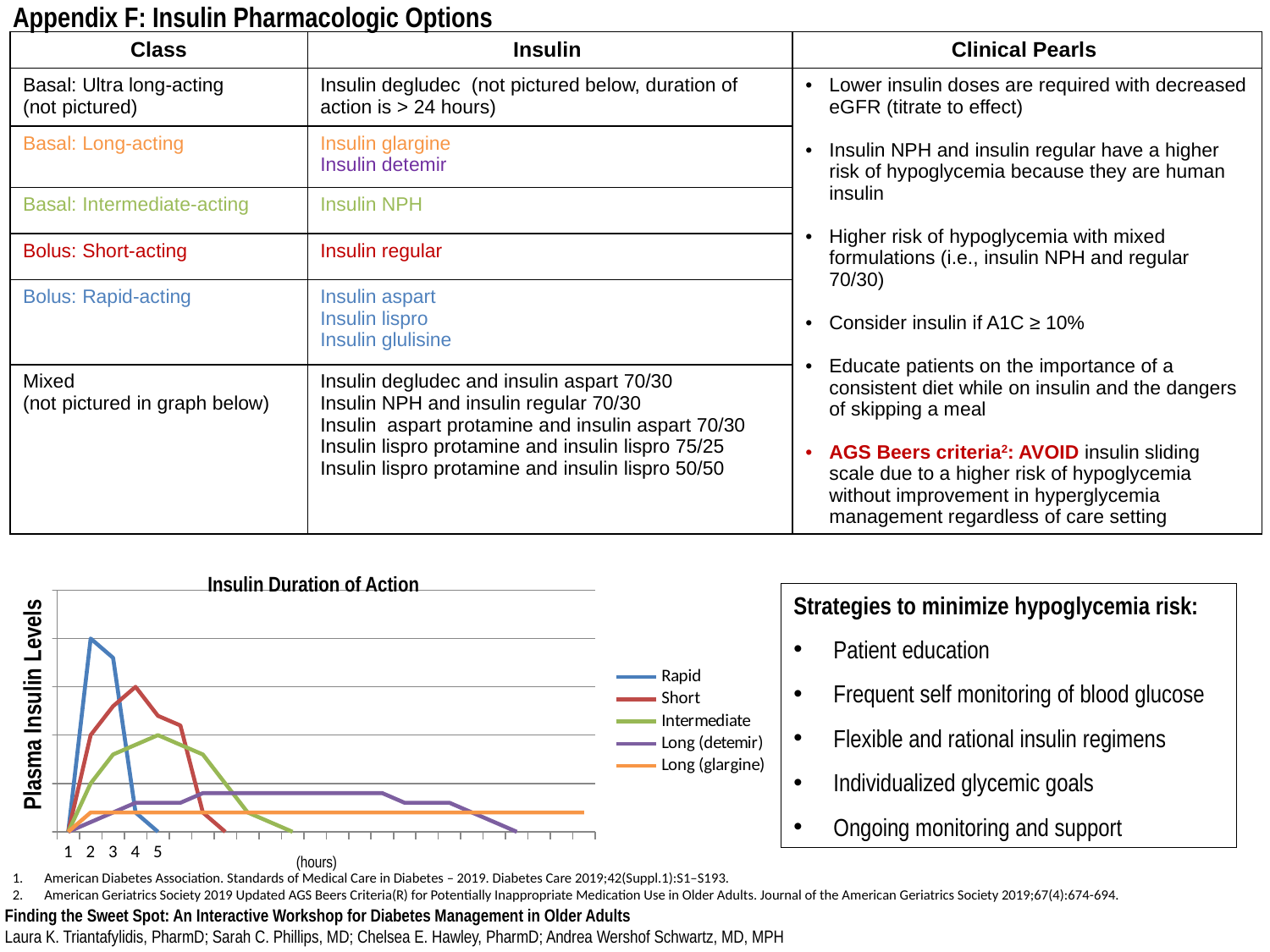

# Appendix F: Insulin Pharmacologic Options
| Class | Insulin | Clinical Pearls |
| --- | --- | --- |
| Basal: Ultra long-acting (not pictured) | Insulin degludec (not pictured below, duration of action is > 24 hours) | Lower insulin doses are required with decreased eGFR (titrate to effect) Insulin NPH and insulin regular have a higher risk of hypoglycemia because they are human insulin Higher risk of hypoglycemia with mixed formulations (i.e., insulin NPH and regular 70/30) Consider insulin if A1C ≥ 10% Educate patients on the importance of a consistent diet while on insulin and the dangers of skipping a meal AGS Beers criteria2: AVOID insulin sliding scale due to a higher risk of hypoglycemia without improvement in hyperglycemia management regardless of care setting |
| Basal: Long-acting | Insulin glargine Insulin detemir | |
| Basal: Intermediate-acting | Insulin NPH | |
| Bolus: Short-acting | Insulin regular | |
| Bolus: Rapid-acting | Insulin aspart Insulin lispro Insulin glulisine | |
| Mixed (not pictured in graph below) | Insulin degludec and insulin aspart 70/30 Insulin NPH and insulin regular 70/30 Insulin aspart protamine and insulin aspart 70/30 Insulin lispro protamine and insulin lispro 75/25 Insulin lispro protamine and insulin lispro 50/50 | |
Insulin Duration of Action
### Chart
| Category | Rapid | Short | Intermediate | Long (detemir) | Long (glargine) |
|---|---|---|---|---|---|Strategies to minimize hypoglycemia risk:
Patient education
Frequent self monitoring of blood glucose
Flexible and rational insulin regimens
Individualized glycemic goals
Ongoing monitoring and support
Plasma Insulin Levels
(hours)
American Diabetes Association. Standards of Medical Care in Diabetes – 2019. Diabetes Care 2019;42(Suppl.1):S1–S193.
American Geriatrics Society 2019 Updated AGS Beers Criteria(R) for Potentially Inappropriate Medication Use in Older Adults. Journal of the American Geriatrics Society 2019;67(4):674-694.
Finding the Sweet Spot: An Interactive Workshop for Diabetes Management in Older Adults
Laura K. Triantafylidis, PharmD; Sarah C. Phillips, MD; Chelsea E. Hawley, PharmD; Andrea Wershof Schwartz, MD, MPH
