# Supplementary material for: Finding the Sweet Spot: An Interactive Workshop on Diabetes Management in Older Adults
Source: MedEdPORTAL. 2019 Oct 18;15:10845. doi: 10.15766/mep_2374-8265.10845 (PMC6944249; doi:10.15766/mep_2374-8265.10845)
Supplement: Supplementary file 1 — A. Presurvey.docx B. Finding the Sweet Spot Slides.pptx C. Finding the Sweet Spot Activity.docx D. Considerations for A1c Targets.pptx E. Noninsulin Pharmacologic Options.pptx F. Insulin Pharmacologic Options.pptx G. Approach to Prescribing and Deprescribing.pptx H. Postsurvey.docx I. Pre- and Postsurvey Answer Guide.docx [file mep-15-10845-s001.zip › G. Approach to Prescribing and Deprescribing.pptx]

## Slide 1
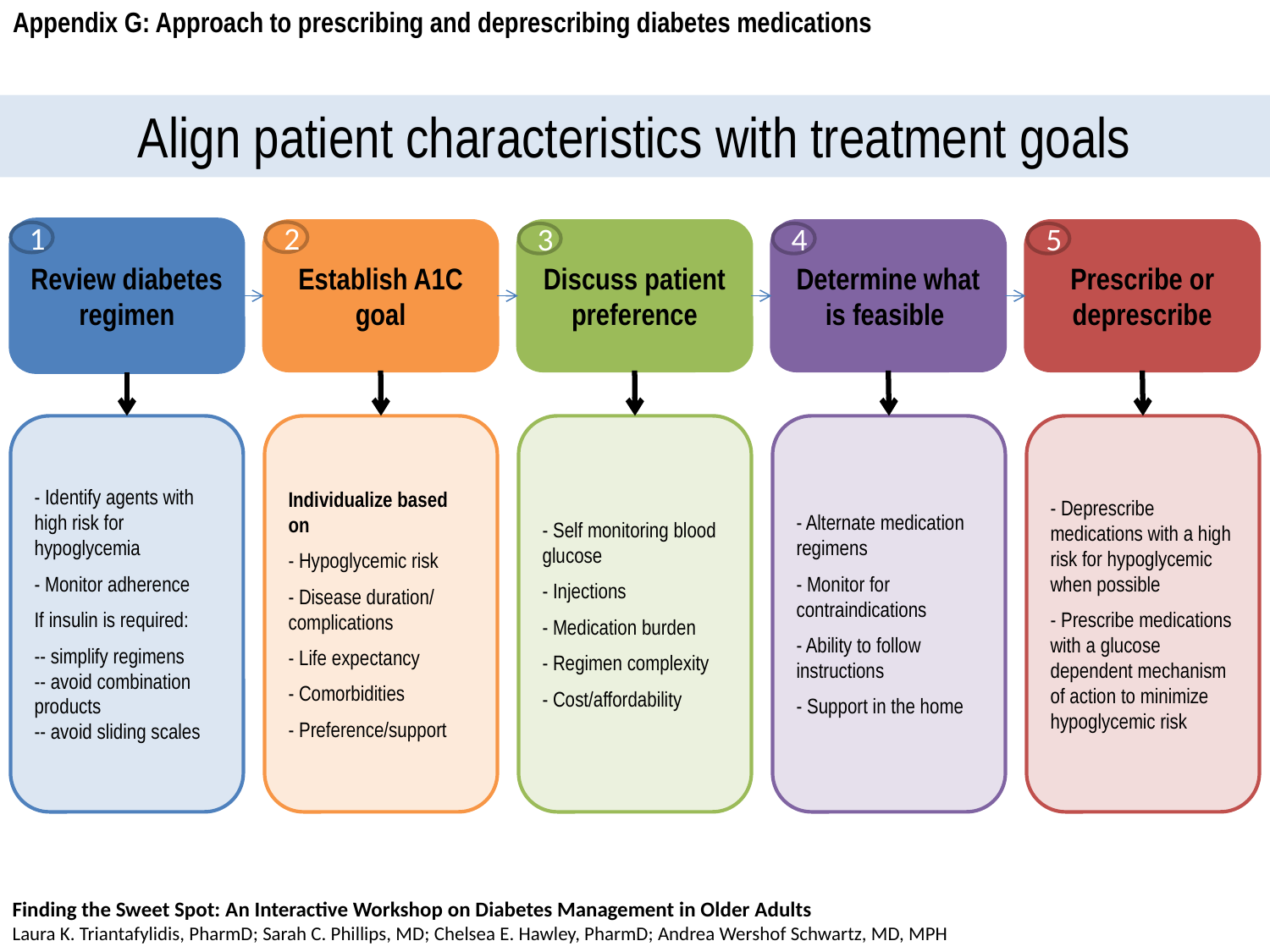

Appendix G: Approach to prescribing and deprescribing diabetes medications
Align patient characteristics with treatment goals
Review diabetes regimen
Establish A1C goal
Discuss patient preference
Determine what is feasible
Prescribe or deprescribe
2
1
3
4
5
- Identify agents with high risk for hypoglycemia
- Monitor adherence
If insulin is required:
-- simplify regimens -- avoid combination products -- avoid sliding scales
Individualize based on
- Hypoglycemic risk
- Disease duration/ complications
- Life expectancy
- Comorbidities
- Preference/support
- Self monitoring blood glucose
- Injections
- Medication burden
- Regimen complexity
- Cost/affordability
- Alternate medication regimens
- Monitor for contraindications
- Ability to follow instructions
- Support in the home
- Deprescribe medications with a high risk for hypoglycemic when possible
- Prescribe medications with a glucose dependent mechanism of action to minimize hypoglycemic risk
Finding the Sweet Spot: An Interactive Workshop on Diabetes Management in Older Adults
Laura K. Triantafylidis, PharmD; Sarah C. Phillips, MD; Chelsea E. Hawley, PharmD; Andrea Wershof Schwartz, MD, MPH

## Slide 2
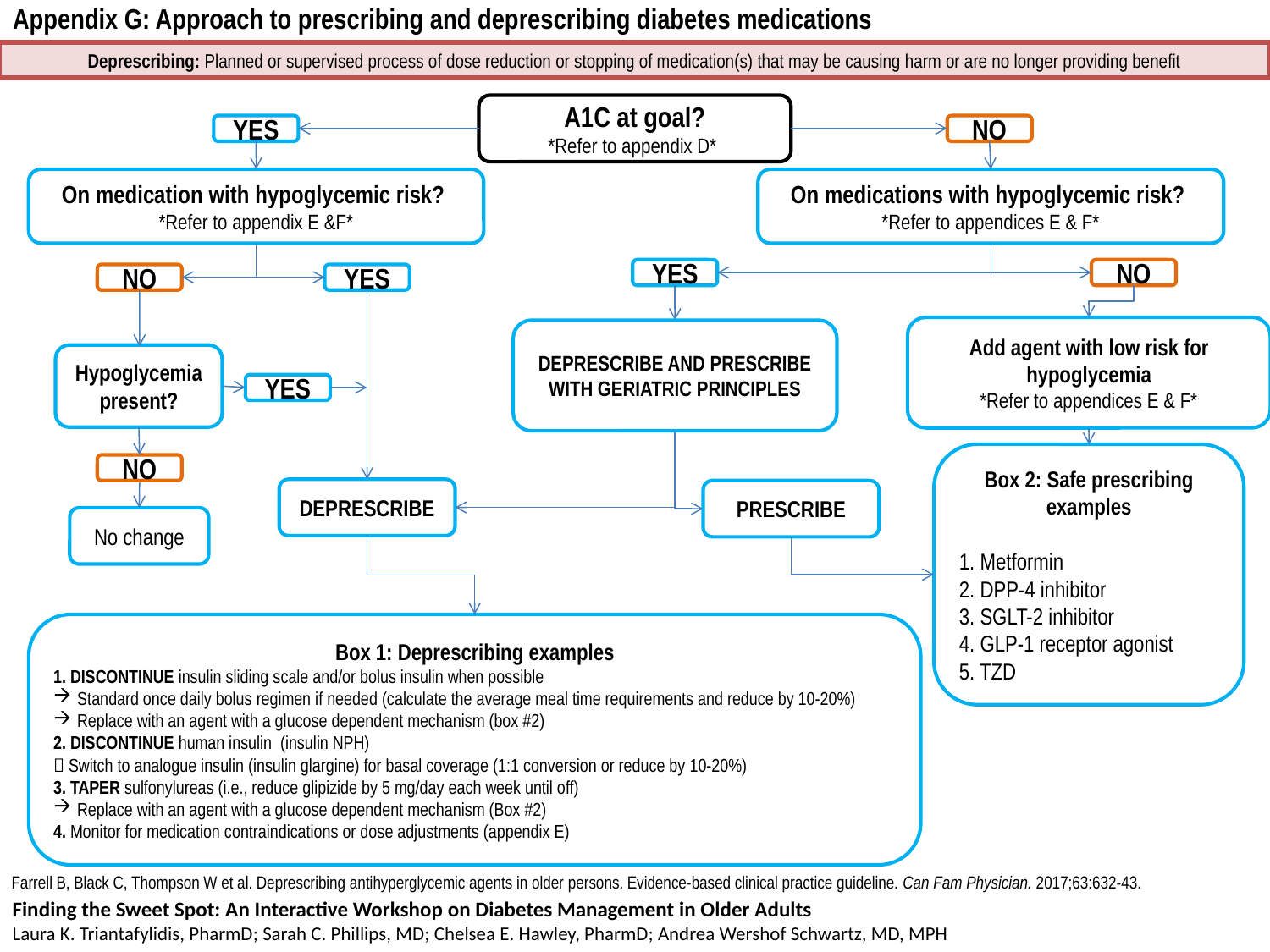

Appendix G: Approach to prescribing and deprescribing diabetes medications
Deprescribing: Planned or supervised process of dose reduction or stopping of medication(s) that may be causing harm or are no longer providing benefit
A1C at goal?
*Refer to appendix D*
YES
NO
On medication with hypoglycemic risk?
*Refer to appendix E &F*
On medications with hypoglycemic risk?
*Refer to appendices E & F*
YES
NO
NO
YES
Add agent with low risk for hypoglycemia
*Refer to appendices E & F*
DEPRESCRIBE AND PRESCRIBE WITH GERIATRIC PRINCIPLES
Hypoglycemia present?
YES
Box 2: Safe prescribing examples
1. Metformin
2. DPP-4 inhibitor
3. SGLT-2 inhibitor
4. GLP-1 receptor agonist
5. TZD
NO
DEPRESCRIBE
PRESCRIBE
No change
Box 1: Deprescribing examples
1. DISCONTINUE insulin sliding scale and/or bolus insulin when possible
Standard once daily bolus regimen if needed (calculate the average meal time requirements and reduce by 10-20%)
Replace with an agent with a glucose dependent mechanism (box #2)
2. DISCONTINUE human insulin (insulin NPH)
 Switch to analogue insulin (insulin glargine) for basal coverage (1:1 conversion or reduce by 10-20%)
3. TAPER sulfonylureas (i.e., reduce glipizide by 5 mg/day each week until off)
Replace with an agent with a glucose dependent mechanism (Box #2)
4. Monitor for medication contraindications or dose adjustments (appendix E)
Farrell B, Black C, Thompson W et al. Deprescribing antihyperglycemic agents in older persons. Evidence-based clinical practice guideline. Can Fam Physician. 2017;63:632-43.
Finding the Sweet Spot: An Interactive Workshop on Diabetes Management in Older Adults
Laura K. Triantafylidis, PharmD; Sarah C. Phillips, MD; Chelsea E. Hawley, PharmD; Andrea Wershof Schwartz, MD, MPH
