# Supplementary material for: Finding the Sweet Spot: An Interactive Workshop on Diabetes Management in Older Adults
Source: MedEdPORTAL. 2019 Oct 18;15:10845. doi: 10.15766/mep_2374-8265.10845 (PMC6944249; doi:10.15766/mep_2374-8265.10845)
Supplement: Supplementary file 1 — A. Presurvey.docx B. Finding the Sweet Spot Slides.pptx C. Finding the Sweet Spot Activity.docx D. Considerations for A1c Targets.pptx E. Noninsulin Pharmacologic Options.pptx F. Insulin Pharmacologic Options.pptx G. Approach to Prescribing and Deprescribing.pptx H. Postsurvey.docx I. Pre- and Postsurvey Answer Guide.docx [file mep-15-10845-s001.zip › I. Pre- and Postsurvey Answer Guide.docx]

Pre- and Post-survey answer matching:

| **Pre-survey question #** | **Post-survey question #** | **Correct Answer** |
| --- | --- | --- |
| 1 | 1 | B |
| 2 | 4 | B |
| 3 | 2 | D |
| 4 | 3 | D |
| 5 | 5 | C |

Questions and answers with rationale:

**Please answer the following questions to the best of your ability (select the best answer choice):**

1. **Which of the following oral diabetes medication has the greatest risk of hypoglycemia when used alone?**
2. Glipizide
3. Glyburide – this sulfonylurea has the longest half-life due to its active metabolites and therefore the highest risk for hypoglycemia; it is included in the AGS Beers Criteria as an agent to avoid in older adults due to hypoglycemic risk. Glipizide also has an increased risk for hypoglycemia compared to metformin and saxagliptin but glyburide has the **greatest** risk.
4. Metformin
5. Saxagliptin
6. **An 81 year old female with type 2 diabetes (A1C 9.7%), hypertension, hyperlipidemia, Parkinson’s disease, heart failure, acid reflux, arthritis and depression is seen in primary care. She is managed with glipizide 10 mg twice daily before meals and is unable to tolerate metformin. Recent labs show normal renal function. What is the next step in diabetes management?**
   1. Start a TZD (pioglitazone) and follow-up with weight check in 1 month
   2. Start an SGLT-2 inhibitor (empagliflozin) and follow-up with BP and BMP in 1 month – The first part of this question is identifying that this patients A1C (9.7%) is above goal, a reasonable goal for this patient is less than 8% given the presence of at least 3 co-existing chronic illnesses (for example: heart failure, arthritis and depression). The goal may be even less stringent depending on the progression of these conditions (i.e., if end-stage, for example stage 3-4 congestive heart failure). Once the A1C goal is determined it may be recognized that this patient requires additional therapy, the next step is to identify the best agent to achieve the determined A1C goal. Pioglitazone should be avoided in heart failure therefore that answer is incorrect. An increase to the glipizide dose would increase this elderly patients risk for hypoglycemia and likely provide minimal additional blood sugar lowering. Starting insulin will likely improve this patients’ blood glucose but will increase the risk for hypoglycemia, another reason to avoid insulin may be the patients Parkinson’s disease which would make injections difficult. An SGLT-2 inhibitor is an attractable option, there is minimal risk for hypoglycemia, it is oral and this patient may also benefit from the cardiovascular benefits from this class.
   3. Increase glipizide to 20 mg twice daily before meals
   4. Start insulin NPH 10 units twice daily
7. **Which insulin has the greatest risk for hypoglycemia when used alone?**
8. Insulin detemir
9. Insulin glargine
10. Insulin NPH
11. Insulin NPH and insulin regular 70/30 – This is the best answer. all insulins increase the risk for hypoglycemia, the greatest risk is human insulins and also with pre-mixed insulins which need to be taken with relation to meals and may be especially dangerous in patients with irregular dietary habits.
12. **An otherwise healthy 88 year old with type 2 diabetes (A1C 7%) is currently managed with metformin 1000 mg twice daily and glipizide 2.5 mg twice daily. He does not check his blood sugar and does endorse occasional dizziness. What change should you make to his regimen?**
13. Advise blood sugar checks to see if dizziness is due to hypoglycemia
14. Reduce the metformin as it is causing hypoglycemia and dizziness
15. Increase metformin to 2000 mg twice daily and stop glipizide
16. Stop glipizide and follow-up in 3 months to see if his A1C is at goal. – This is the best answer. Answer B is not correct as metformin has a glucose-dependent mechanism of action and therefore minimal risk for hypoglycemia. Answer C is incorrect because this exceeds the maximum dose of metformin. While answer A, is not incorrect, it leaves the patient at an increased risk for hypoglycemia, which based on symptom reports, this patient is likely experiencing. The goal of this question is for the learner to recognize that this 88-year old’s A1C is likely well below goal (even without knowing the specifics of their past medical history). While there is limited information provided related to the patient’s comorbid conditions, it is likely an appropriate individualized A1C goal for this patient is at least < 8%. Given this, it is reasonable to stop the glipizide to alleviate the risk of hypoglycemia and recheck the A1Cin 3 months, at that time, if the A1C has risen to an unacceptable level, alternate agents with a lower risk may be initiated. Additionally, this patient is on a relatively lower dose of glipizide, knowing the dose dependent potential A1C lowering of the various diabetes agents, it is expected that removing this medicine will have little impact on the patients A1C.
17. **A 75 year old nursing-home patient with type 2 diabetes is noted to have frequent hypoglycemia. She currently receives insulin NPH 20 units twice daily. Other relevant medical history includes severe cognitive impairment, oxygen-dependent chronic obstructive pulmonary disease, hypertension, and depression. What is your next step for management?**
18. Reduce insulin NPH to 20 units once daily
19. Stop insulin NPH, start a sliding scale correctional insulin
20. Change insulin NPH to a once daily long acting basal insulin (insulin glargine) –. Human insulins (insulin NPH) have a higher risk for hypoglycemia compared to insulin analogues (insulin glargine), therefore changing to insulin glargine, may help minimize the risk for hypoglycemia. Additionally, based on the duration/onset of these insulins (refer to the chart in appendix F), insulin glargine does not have a peak and rather has a sustained steady effect over a 24-hour period, where-as the NPH insulin (intermediate insulin) has a peak which may result in an acute low blood sugar. Answer A is also incorrect because insulin NPH does not provide 24-hour coverage. Answer B is also incorrect because sliding scales are listed in the AGS Beers Criteria as regimens to avoid given they have demonstrated increased risk with no additional benefits. Answer D is incorrect because the patient would remain on a human insulin (insulin NPH) which still poses a risk for hypoglycemia compared to insulin analogues (insulin glargine).
21. Reduce insulin NPH to 10 units twice daily
